# Mobile apps to reduce depressive symptoms and alcohol use in youth: A systematic review and meta‐analysis: A systematic review

**DOI:** 10.1002/cl2.1398

**Published:** 2024-04-26

**Authors:** Olivia Magwood, Ammar Saad, Dominique Ranger, Kate Volpini, Franklin Rukikamirera, Rinila Haridas, Shahab Sayfi, Jeremie Alexander, Yvonne Tan, Kevin Pottie

**Affiliations:** ^1^ Bruyère Research Institute Ottawa Ontario Canada; ^2^ Interdisciplinary School of Health Sciences, Faculty of Health Sciences University of Ottawa Ottawa Ontario Canada; ^3^ School of Epidemiology and Public Health University of Ottawa Ottawa Ontario Canada; ^4^ Department of Family Medicine University of Ottawa Ottawa Ontario Canada; ^5^ Faculty of Medicine University of British Columbia Vancouver British Columbia Canada; ^6^ School of Nursing University of British Columbia Vancouver British Columbia Canada; ^7^ Department of Biology, Faculty of Science University of Ottawa Ottawa Ontario Canada; ^8^ Department of Biochemistry and Biomedical Science McMaster University Hamilton Ontario Canada; ^9^ Department of Biomedical and Molecular Sciences, Faculty of Arts and Science Queen's University Kingston Ontario Canada; ^10^ Departments of Family Medicine and Epidemiology and Biostatistics Western University London Ontario Canada; ^11^ Department of Family Medicine Dalhousie University Halifax Nova Scotia Canada

## Abstract

**Background:**

Among youth, symptoms of depression, anxiety, and alcohol use are associated with considerable illness and disability. Youth face many personal and health system barriers in accessing mental health care. Mobile applications (apps) offer youth potentially accessible, scalable, and anonymous therapy and other support. Recent systematic reviews on apps to reduce mental health symptoms among youth have reported uncertain effectiveness, but analyses based on the type of app‐delivered therapy are limited.

**Objectives:**

We conducted this systematic review with youth co‐researchers to ensure that this review addressed the questions that were most important to them. The objective of this review is to synthesize the best available evidence on the effectiveness of mobile apps for the reduction of depressive symptoms (depression, generalized anxiety, psychological distress) and alcohol use among youth.

**Search Methods:**

We conducted electronic searches of the following bibliographic databases for studies published between January 1, 2008, and July 1, 2022: MEDLINE (via Ovid), Embase (via Ovid), PsycINFO (via Ovid), CINAHL (via EBSCOHost), and CENTRAL (via the Cochrane Library). The search used a combination of indexed terms, free text words, and MeSH headings. We manually screened the references of relevant systematic reviews and included randomized controlled trials (RCTs) for additional eligible studies, and contacted authors for full reports of identified trial registries or protocols.

**Selection Criteria:**

We included RCTs conducted among youth aged 15–24 years from any setting. We did not exclude populations on the basis of gender, socioeconomic status, geographic location or other personal characteristics. We included studies which assessed the effectiveness of app‐delivered mental health support or therapy interventions that targeted the management of depressive disorders and/or alcohol use disorders. We excluded apps that targeted general wellness, apps which focused on prevention of psychological disorders and apps that targeted bipolar disorder, psychosis, post‐traumatic stress disorder, attention‐deficit hyperactivity disorder, substance use disorders (aside from alcohol), and sleep disorders. Eligible comparisons included usual care, no intervention, wait‐list control, alternative or controlled mobile applications. We included studies which reported outcomes on depressive symptoms, anxiety symptoms, alcohol use and psychological distress over any follow‐up period.

**Data Collection and Analysis:**

We standardized the PICO definitions (population, intervention, comparison, and outcome) of each included study and grouped studies by the type of therapy or support offered by the app. Whenever app design and clinical homogeneity allowed, we meta‐analyzed outcomes using a random‐effects model. Outcome data measured using categorical scales were synthesized using odds ratios. Outcome data measured using continuous scales were synthesized as the standardized mean difference. We assessed the methodological quality of each included study using the Cochrane Risk of Bias 2.0 tool and we assessed certainty of the evidence using the GRADE approach.

**Main Results:**

From 5280 unique citations, we included 36 RCTs published in 37 reports and conducted in 15 different countries (7984 participants). Among the 36 included trials, we assessed two with an overall low risk of bias, 8 trials with some concern regarding risk of bias, and 26 trials with a high risk of bias. Interventions varied in the type of therapy or supports offered. The most common intervention designs employed mindfulness training, cognitive behavioral therapy (CBT), or a combination of the two (mindfulness + CBT). However, other interventions also included self‐monitoring, medication reminders, cognitive bias modification or positive stimulation, dialectical behavioral therapy, gamified health promotion, or social skill building. Mindfulness apps led to short term improvements in depressive symptoms when compared to a withheld control (SMD = −0.36; 95% CI [−0.63, −0.10]; *p* = 0.007, *n* = 3 RCTs, GRADE: very low certainty) and when compared to an active control (SMD = −0.27; 95% CI [−0.53, −0.01]; *p* = 0.04, *n* = 2 RCTs, GRADE: very low). Apps delivering this type of support also significantly improved symptoms of anxiety when compared to a withheld control (SMD = −0.35; 95% CI [−0.60, −0.09]; *p* = 0.008, *n* = 3 RCTs, GRADE: very low) but not when compared to an active control (SMD = −0.24; 95% CI [−0.50, 0.02]; *p* = 0.07, *n* = 2 RCTs, GRADE: very low). Mindfulness apps showed improvements in psychological stress that approached statistical significance among participants receiving the mindfulness mobile apps compared to those in the withheld control (SMD = −0.27; 95% CI [−0.56, 0.03]; *p* = .07, *n* = 4 RCTs, GRADE: very low). CBT apps also led to short‐term improvements in depressive symptoms when compared to a withheld control (SMD = −0.40; 95% CI [−0.80, 0.01]; *p* = 0.05, *n* = 2 RCTs, GRADE: very low) and when compared to an active control (SMD = −0.59; 95% CI [−0.98, −0.19]; *p* = 0.003, *n* = 2 RCTs, GRADE: very low). CBT‐based apps also improved symptoms of anxiety compared to a withheld control (SMD = −0.51; 95% CI [−0.94, −0.09]; *p* = 0.02, *n* = 3 RCTs, GRADE: very low) but not when compared to an active control (SMD = −0.26; 95% CI [−1.11, 0.59]; *p* = 0.55, *n* = 3 RCTs, GRADE: very low). Apps which combined mindfulness and CBT did not significantly improve symptoms of depression (SMD = −0.20; 95% CI [−0.42, 0.02]; *p* = 0.07, *n* = 2 RCTs, GRADE: very low) or anxiety (SMD = −0.21; 95% CI [−0.49, 0.07]; *p* = 0.14, *n* = 2 RCTs, GRADE: very low). However, these apps did improve psychological distress (SMD = −0.43; 95% CI [−0.74, −0.12]; *p* = 0.006, *n* = 2 RCTs, GRADE: very low). The results of trials on apps to reduce alcohol use were inconsistent. We did not identify any harms associated with the use of apps to manage mental health concerns. All effectiveness results had a very low certainty of evidence rating using the GRADE approach, meaning that apps which deliver therapy or other mental health support may reduce symptoms of depression, anxiety and psychological distress but the evidence is very uncertain.

**Authors' Conclusions:**

We reviewed evidence from 36 trials conducted among youth. According to our meta‐analyses, the evidence is very uncertain about the effect of apps on depression, anxiety, psychological distress, and alcohol use. Very few effects were interpreted to be of clinical importance. Most of the RCTs were small studies focusing on efficacy for youth at risk for depressive symptoms. Larger trials are needed to evaluate effectiveness and allow for further analysis of subgroup differences. Longer trials are also needed to better estimate the clinical importance of these apps over the long term.

## 1 PLAIN LANGUAGE SUMMARY

### 1.1 Apps that use Cognitive Behavior Therapy (CBT) and mindfulness reduce mental health symptoms among young people

Mobile applications (apps) available on smartphones can be used to manage mental health symptoms. Among young people, we found that apps which deliver mindfulness‐based training or CBT may reduce symptoms of depression and anxiety, but the evidence is very uncertain.

#### 1.1.1 What is this review about?

Mental health conditions among young people aged 15–24 years are of increased concern. Young people face many limitations in accessing mental health care in the community, including a lack of access to and awareness among primary care physicians, teachers, and parents. Smartphone applications (apps) offer young people the opportunity to manage their mental health symptoms and help them overcome barriers to accessing care. This review considers how apps are designed and whether they improve mental health symptoms (depression, anxiety, distress, alcohol use) among young people. We include young people as equal members of the research team to guide our approach.

#### 1.1.2 What is the aim of this review?

This review aims to summarize the evidence on smartphone apps and determine whether these apps improve symptoms of depression, anxiety, psychological distress and alcohol use among young people aged 15–24 years old.

#### 1.1.3 What are the main findings of this review?

We identified 36 trials that included 7984 young people from 15 different countries. We categorized these trials based on the type of support or therapy delivered by the app: (i) mindfulness‐based apps, (ii) apps which delivered CBT, (iii) apps that delivered CBT and mindfulness together, and (iv) other app designs. These other designs included gamification, motivational strategies, and medication reminders.

We found that apps which delivered mindfulness training or CBT significantly improved short term symptoms of depression and anxiety, however this evidence is very uncertain. Apps which combined these two design elements reduced psychological distress. While many apps aimed to reduce alcohol use, the results were inconsistent and more research is needed. Importantly, we did not identify any harms in using these apps to manage mental health symptoms.

#### 1.1.4 What do the findings of this review mean?

Mobile apps that deliver CBT or mindfulness training may reduce symptoms of depression and anxiety, but the evidence is uncertain. We have very limited information about whether these apps reduce alcohol use. This uncertainty is due to the small sample sizes of the trials and concerns about how the trials were conducted. More research is needed to determine whether or not these designs are effective and should be recommended for young people. Future research should consider conducting trials over longer periods of time with more participants.

#### 1.1.5 How up‐to‐date is this review?

This review includes evidence published up to July 1, 2022.

## 2 SUMMARY OF FINDINGS

**Table:** 

**Summary of findings 1**			
Mindfulness‐based mobile apps compared to withheld control (no intervention or wait listing) for improving mental health outcomes			

Outcome no. of participants (studies)	Relative effect (95% CI)	Anticipated absolute effects (95% CI) – Difference	Certainty
Severity of depression no. of participants: 251 (3 RCTs)	–	SMD 0.36 SD lower (0.63 lower to 0.1 lower)	⨁◯◯◯ Very low^a,b^
Severity of anxiety symptoms № of participants: 240 (3 RCTs)	–	SMD 0.35 SD lower (0.6 lower to 0.09 lower)	⨁◯◯◯ Very low^a,b^
Psychological stress no. of participants: 500 (4 RCTs)	–	SMD 0.27 SD lower (0.56 lower to 0.03 higher)	⨁◯◯◯ Very low^b,c^
**Explanations**: a. Three studies had high concerns of risk of bias with randomization process, deviation from intended interventions, missing outcome data, and measurement of the outcome, and one study had some concerns of risk of bias due to deviation from intended interventions and missing outcome data. b. The optimal information size was not reached. c. Four studies had high concerns of risk of bias with randomization process, deviation from intended interventions, missing outcome data, and measurement of the outcome, and one study had some concerns of risk of bias due to deviation from intended interventions and missing outcome data.			

**Table:** 

**Summary of findings 2**			
Mindfulness‐based mobile apps compared to an active control (i.e., no intervention, waitlisting) for improving mental health outcomes			

**Outcome no. of participants (studies)**	**Relative effect (95% CI)**	**Anticipated absolute effects (95% CI) – Difference**	**Certainty**
Severity of depression № of participants: 233 (2 RCTs)	–	SMD 0.27 SD lower (0.53 lower to 0.01 lower)	⨁◯◯◯ Very low^a,b^
Severity of anxiety № of participants: 233 (2 RCTs)	–	SMD 0.24 SD lower (0.5 lower to 0.02 higher)	⨁◯◯◯ Very low^a,^
**Explanations**: a. Two studies had high concerns of risk of bias with randomization process, deviation from intended interventions, missing outcome data, and measurement of the outcome, and one study had some concerns of risk of bias due to deviation from intended interventions and missing outcome data. b. The optimal information size was not reached.			

**Table:** 

**Summary of findings 3**			
CBT‐based mobile apps compared to a withheld control (i.e., no intervention, waitlisting) for improving mental health outcomes			

**Outcome no. of participants (studies)**	**Relative effect (95% CI)**	**Anticipated absolute effects (95% CI) – Difference**	**Certainty**
Severity of depressive outcomes no. of participants: 180 (2 RCTs)	–	SMD 0.4 SD lower (0.8 lower to 0.01 higher)	⨁◯◯◯ Very low^a,b^
Severity of anxiety outcomes № of participants: 210 (3 RCTs)	–	SMD 0.51 SD lower (0.94 lower to 0.09 lower)	⨁◯◯◯ Very low^b,c^
**Explanations** a. Two studies had high concerns of risk of bias with randomization process, deviation from intended interventions, missing outcome data, and measurement of the outcome, and one study had some concerns of risk of bias due to deviation from intended interventions and missing outcome data. b. The optimal information size was not reached. c. Three studies had high concerns of risk of bias with randomization process, deviation from intended interventions, missing outcome data, and measurement of the outcome, and one study had some concerns of risk of bias due to deviation from intended interventions and missing outcome data.			

**Table:** 

**Summary of findings 4**			
CBT‐based mobile apps compared to an active control (i.e., controlled mobile app) for improving mental health outcomes			

**Outcome no. of participants (studies)**	**Relative effect (95% CI)**	**Anticipated absolute effects (95% CI) – Difference**	**Certainty**
Severity of depression outcomes no. of participants: 104 (2 RCTs)	–	SMD 0.59 SD lower (0.98 lower to 0.19 lower)	⨁◯◯◯ Very low^a,b^
Severity of anxiety outcomes № of participants: 104 (2 RCTs)	–	SMD 0.26 SD lower (1.11 lower to 0.59 higher)	⨁◯◯◯ Very low^a,b,c^
**Explanations**: a. Two studies had high concerns of risk of bias with randomization process, deviation from intended interventions, missing outcome data, and measurement of the outcome, and one study had some concerns of risk of bias due to deviation from intended interventions and missing outcome data. b. The optimal information size was not reached. c. The variation of effect sizes, large *I* ^2^ size, *p*‐value smaller than 0.05, and limited overlap between 95% CI led to very serious inconsistency across the two studies.			

**Table:** 

**Summary of findings 5**			
Combination therapy (CBT and mindfulness) compared to a withheld control (i.e., no intervention, waitlisting) for improving mental health outcomes			

**Outcome no. of participants (studies)**	**Relative effect (95% CI)**	**Anticipated absolute effects (95% CI) – Difference**	**Certainty**
Severity of depression outcomes no. of participants: 341 (2 RCTs)	–	SMD 0.2 SD lower (0.42 lower to 0.02 higher)	⨁◯◯◯ Very low^a,b^
Severity of anxiety outcomes no. of participants: 193 (2 RCTs)	–	SMD 0.21 SD lower (0.49 lower to 0.07 higher)	⨁◯◯◯ Very low^b,c^
Psychological stress no. of participants: 164 (2 RCTs)	–	SMD 0.43 SD lower (0.74 lower to 0.12 lower)	⨁◯◯◯ Very low^b,c^
**Explanations**: a. Two studies had high concerns of risk of bias with randomization process, deviation from intended interventions, missing outcome data, and measurement of the outcome, and one study had some concerns of risk of bias due to deviation from intended interventions and missing outcome data. b. The optimal information size was not reached. c. One study had high concerns of risk of bias with randomization process, deviation from intended interventions, missing outcome data, and measurement of the outcome, and one study had some concerns of risk of bias due to deviation from intended interventions and missing outcome data.			

## 3 BACKGROUND

### 3.1 The problem, condition or issue

Symptoms of depression, anxiety, and behavioral disorders are associated with considerable illness and disability among youth and have worsened during the COVID‐19 pandemic (WHO, 2021; Racine, 2021). Depressive symptoms among youth doubled in the first year of the pandemic, suggesting that 1 in 4 youth globally are experiencing clinically elevated depression symptoms (Racine, 2021). As a consequence, an increase in demand for mental health care utilization among youth is expected. However, youth face many personal, societal, and health system‐related barriers that reduce access to services for mental health and/or substance use disorders. The COVID‐19 pandemic created sustained disruption in society and schools. Public health restrictions caused isolation and reduced social and emotional support (Glover, 2020). Together, these factors have exacerbated depressive symptoms in a pre‐existing youth global public health emergency (Racine, 2021).

Failing to address youth mental health conditions means conditions will often extend to adulthood, impairing both physical and mental health and limiting opportunities to lead fulfilling lives (WHO, 2021). Even before the pandemic, 10%–20% of youth worldwide suffered from a mental health disorder, with half of all mental health conditions starting by 14 years of age (WHO, 2018). Physical, emotional, and social changes such as exposure to poverty, abuse, or violence can increase their risk of depressive disorders and alcohol use (Saluja, 2004). Post‐secondary students, for example, are faced with stress from increased and on‐line academic demands, adjusting to a new environment, and developing a new support system. Under such conditions, depressive disorder diagnoses are common among students, with one in four students treated for a mental disorder, one in five reporting thoughts about suicide, 9% reporting having attempted suicide and nearly 20% reporting self‐injury (Liu, 2019).

Several evidence‐based therapies and supports are available to assist youth to manage mental health concerns, including two psychotherapies recommended by the APA clinical practice guideline for treatment of adolescents with depression: CBT and interpersonal therapy (American Psychological Association [APA], 2023). CBT is the most widely researched intervention of youth depression (Freire, 2014). It is a manual‐based, time‐limited intervention that aims to understand and modify maladaptive cognitions and behaviors and improve mood. For more severe depression, dialectical behavior therapy (DBT) was adapted from CBT to help people who who have trouble managing painful emotions, who engage in risky behavior, self‐harm, or who have suicidal thoughts to assist them to manage intense emotions (Child Mind Institute, 2023). Mindfulness‐based cognitive therapy, which combines CBT methods with mindfulness, has also been shown to help youth with depression (Child Mind Institute, 2023). Despite the existence of these therapies, they often exist behind significant barriers to accessing care.

Youth with depressive disorders or substance use disorders rarely access mental health or primary care services due to personal and systemic barriers (Ross, 2018). The social stigma of asking for help, limitations in community services, and lack of awareness amongst family physicians, teachers, and parents often leave youth alone and untreated (Cash, 2018; Malla, 2019). However, failure to receive proper mental healthcare has grave consequences for youth, including disabling depression, suicide, expulsion in early education, later unemployment and higher incarceration rates (Cash, 2018). Furthermore, not only do youth not learn about the etiology of their mental health symptoms, many conditions may persist into adulthood although studies suggest symptoms of depressive disorders are more easily treated and prevented during adolescence (Pedrelli, 2015; NIMH, 2021).

Given the stigma associated with accessing mental health services, mobile applications (apps) offer a unique opportunity to provide mental health support in a non‐judgemental, readily accessible, and large‐scale manner. With greater than 8.93 million apps available for mobile download (Koetsier, 2020), applying a digital treatment approach to depressive disorder and alcohol use may offer a means to help manage the accentuated youth mental health needs. Moreover, youth are uniquely positioned to benefit from digital health resources given their increasing acceptance and usage of technology (Hyden, 2011). Mobile apps may also offer a solution to youth who would otherwise lack the independence to access treatment on their own or would prefer the discretion and anonymity that digital platforms can offer (Grist, 2017).

Apps are available in many designs with different features, but they often aren't “reinventing the wheel” – they build on decades of psychological research on the foundational principles to mental health treatment. For example, the delivery of CBT builds on decades of empirical work understanding the psychological construct that individuals' interpretations of situations influence their reaction (emotional, behavioral, physiological). However, one important continuing challenge for researchers and clinicians is to develop ways to deliver quality CBT treatment to the individuals who need it most. This involves both adapting treatment for diverse populations and creating effective and efficient treatment delivery models, including the expansion of digital methods of delivery (Beck, 2021). Indeed, examining the effectiveness of digital mental health interventions is a current research priority (Hollis, 2018). With so many apps to choose from, youth, clinicians, parents and other trusted support persons need more information about the type of app‐delivered therapy that, if any, work best for addressing their mental health concerns. Recent systematic reviews on standalone mobile apps for mental illness symptoms report promising results (Wang, 2018; Lecomte, 2020; Miralles, 2020; Leech, 2021; Weisel, 2019), but analyses based on the type of therapy of supports they deliver are limited and certainty of evidence remains low.

### 3.2 Description of the condition

The COVID‐19 pandemic had a grave effect on depressive symptoms and alcohol use in adolescents and youth. Depressive symptoms in youth doubled in the first year of the COVID‐19 pandemic and pooled estimates suggest that 1 in 4 youth globally are experiencing clinically elevated depression symptoms and that an increase in mental health care utilization is expected (Racine, 2021).

Heavy use of alcohol has increased 43% in youth, comparing 2012 with 2020 data (Pollard, 2020). The COVID‐19 pandemic created sustained disruption in society, schools, and universities, including increasing poverty and discrimination and public health restrictions have caused isolation and reduced social and emotional support (Glover, 2020). Together, these factors make depressive symptoms in youth a global public health emergency (Racine, 2021).

### 3.3 Description of the intervention

This systematic review focuses on standalone mental health mobile apps. Mobile apps are applications that are available on a mobile device (smartphone or tablet), which can be used independently by the public for assessment, management, and treatment. They are intended to support users at any time or place in the absence of direct interaction with a healthcare provider or specialist.

Mobile mental health apps can target a broad range of psychological disorders and vary in design and functionality. Some, for example, use CBT (e.g., Woebot®), mindfulness training (Mindspace®, Calm®), mood monitoring, cognitive skills training or other designs to treat depressive symptoms. Others use gamification (Boyle, 2017; Earle, 2018) to support the reduction of alcohol use. This review focuses on the effectiveness of various app‐delivered therapy and support for the management of depressive disorders and alcohol use in youth.

Standalone mental health apps can span all stages of clinical care provision, including immediate crisis intervention, prevention, diagnosis, primary treatment, supplement to in‐person therapy, and post‐treatment condition management (Chandrashekar, 2018). Self‐management apps, for example, allow the user to catalog symptoms, set up medication reminders, provide tools or help develop skills such as mindfulness for managing stress, anxiety, or sleep problems. Other apps may help improve thinking skills, illness management, and connect patients with online support groups or healthcare professionals.

Mobile apps also provide users with the opportunity to increase their awareness about their symptoms or daily habits which contribute to depressive disorders. With increased awareness comes more opportunities to intervene (Prentice, 2014). Furthermore, for youth in particular, there is growing evidence addressing depressive disorders and alcohol use may be a positive step toward developing social and emotional capabilities that could influence educational attainment, employment, and health (McNeil, 2012). Multipurpose or treatment apps may also reduce symptoms and the need for in‐person appointments with clinicians, alleviating their workloads (Van Ameringen, 2017).

### 3.4 How the intervention might work

Mobile apps can be used almost anytime and anywhere, making them ideal for youth facing assessment or treatment barriers. With the cost of mobile apps being significantly less than that of traditional care, they could also provide care to where help may not be affordable or available. Additionally, given the stigma surrounding mental health, mobile apps offer a unique anonymous and non‐judgemental first step for those who may have avoided services for mental health concerns in the past. Anonymity may also be beneficial for youth who would otherwise lack the autonomy to access treatment on their own or simply do not know where to start.

**Figure 1:**
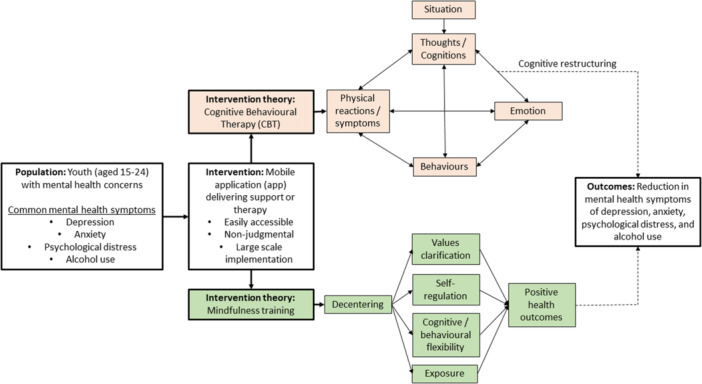
Conceptual framework for Cognitive Behavior Therapy (CBT) and mindfulness apps which address common mental health symptoms among youth.

The precise mechanisms through which apps can assist youth in addressing mental health concerns depends on the design of the app itself – that is, the type of psychological theory, “treatment type” or supports offered to youth by the app. Given the current recommendations for CBT and its derivative treatment types for youth, it is sensible that apps could also apply these treatment modalities through a digital approach. Figure 1 summarizes the potential conceptual pathways for these digitally provided treatments for CBT and mindfulness therapy (Figure 1). We acknowledge that other types of therapies, or combinations of these therapies, could be available through apps. We provide this figure as an example of the potential conceptual pathways, but recognize that many other pathways could exist and are in need of further exploration.

In summary, youth experiencing common mental health symptoms may seek out accessible and non‐judgemental apps to address these concerns. An app delivering CBT, for example, hypothesizes that youth's emotions and behaviors are influenced by their perceptions of events (Fenn, 2013). The app can assist youth to teach them to be their own therapist, by helping them to understand their current ways of thinking and behaving, and by equipping them with the tools to change their maladaptive cognitive and behavioral patterns (Fenn, 2013). Apps can apply a wide range of psychological theories. Another alternative is mindfulness training. This model describes mindfulness as the ability to reperceive (also known as decentering). Decentering allows youth to stand back and witness the drama of their life without being personally immersed and engaging with it (Shapiro, 2006). Therefore, decentering through mindfulness allows youth to clarify their values and thereby better meet their needs and interests, which produces more guided attention and facilitates the achievement of various goals that may bring about greater health and wellbeing (Halliday, 2018).

However, as with any health intervention or advancement in technology, there is the potential for harm. Mobile mental health apps are not generally distributed through healthcare providers or settings (Leigh, 2015), rather, users are generally left to evaluate their app choices based on user ratings (Chiauzzi, 2019). Unfortunately, these ratings are based on subjective experiences of users, typically from a usability or visual standpoint, and are not always reflective of an app's quality in terms of improving health outcomes (Bidargaddi, 2017) or adherence to best‐practice treatment guidelines (Nicholas, 2017). In fact, there are no industry‐wide standards to help consumers know if an app has been proven to be effective. As such, there is concern that if a mobile app promises more than it delivers, and provides no clinical oversight, consumers may turn away from other, more effective mental health interventions. Data security for mental health apps is another widespread concern (Powell, 2014). These apps deal with very sensitive personal information; mobile app product makers must be able to guarantee privacy. Furthermore, many available mental health mobile apps target specific disorders and label their users with a diagnosis which can be harmful and stigmatizing (Moses, 2009). Finally, there is increasing concern for digital dependency, especially among youth. The relationship between app exposure and health in adolescents may follow an “inverted U” pattern, that is, very high exposure and very low exposure might both be associated with poorer mental health outcomes than moderate amounts of usage (Christakis, 2019).

### 3.5 Why it is important to do this review

Standalone mobile apps could become scalable interventions for youth facing mental health symptoms and alcohol use around the world. The design and functionality of mobile apps continues to improve (Lecomte, 2020) and more than 8.93 million apps have emerged on the public market (Koetsier, 2020). At the same time, the COVID‐19 pandemic has created isolation, lockdowns and unemployment has increased depressive disorders and alcohol use in youth (Racine, 2021). Systematic reviews on specific app design and approaches are very rare and much needed to better understand the emerging app evidence base.

The prevalence of youth experiencing depressive symptoms and alcohol use disorders has more than doubled during the COVID‐19 pandemic (Racine, 2021). Despite this high prevalence, youth with mental health and/or substance use disorders rarely access mental health services due to personal and systemic barriers (Ross, 2018). As adolescents are still developing, this period in their lives is critical for their physical and mental well‐being (Kessler, 2007). At the same time, accessible services to match the unique needs of youth populations are limited (Malla, 2018). Health inequities put populations who are already socially disadvantaged (e.g., by virtue of being poor, female, Indigenous or members of a disenfranchised racial, ethnic, or religious group) at further disadvantage with respect to their health (Braveman, 2003). Recent user evidence suggests that mobile apps are most commonly designed for, and used by, white youth, predominantly women, and there is a need to extend their development for culturally and linguistically diverse populations.

Community clinicians need to know more about the effectiveness of mobile apps for depressive disorders and alcohol in youth. Clinicians find it difficult to recommend apps since ratings and reviews are not standardized or available as reliable sources (Marshall, 2020). In addition, there may be future intervention opportunities such as combining medication, existing talk therapies and mobile apps to complement treatment programs (Dellabella, 2018; Truschel, 2021).

Five systematic reviews have been published on the use of standalone mobile apps for common mental illness symptoms and alcohol use in the past few years (Lecomte, 2020; Leech, 2021; Miralles, 2020; Wang, 2018; Weisel, 2019). Lecomte (2020) presented a meta review of 7 meta‐analyses and showed results focusing on apps for depressive symptoms and anxiety were of higher quality and showed small but favorable effect sizes, while apps focusing on stress, emotional health had no significant effects. Only Leech (2021) reviewed apps with a focus on adolescents and youth and they reported similarly some favorable small effects on depression and anxiety and importantly, they did not find age to be a significant effect size modifier. Leech (2021) discussed how apps are able to teach mindfulness techniques to youth. In general, these reviews often pooled many app designs together, thus creating conditions of high design heterogeneity. Viewed as a group of mixed‐designs, robust systematic studies have been limited, results have been uncertain, and long‐term outcomes (12+ weeks) were rarely measured. In addition, results on population sociocultural diversity and possible harms of digital interventions are very rare.

In summary, very few reviews have focused on the effectiveness of specific mobile app designs for depressive symptoms and alcohol use in youth. New mobile apps and app designs continue to emerge and be released to the public, but without clear indication of effectiveness in culturally diverse communities, harms, and privacy concerns (Grist, 2017). The heterogeneity in the definition of the intervention, dosage, duration of interventions, classification of diseases, and selection of populations may lead to a distorted portrait of the added value of mobile apps. Independent evaluation of mobile apps is also lacking (Marshall, 2020).

Several countries, such as the England and Australia have led in the implementation of nation‐wide strategies to prioritize the use of digital technologies in healthcare with policies such as Future in Mind, Five Year Forward, and the UK government's new health education system as of 2020 (Department, 2019; NHS, 2015). Furthermore, the World Health Organization's “QualityRights” pushes for access to mental health services for people around the world (WHO, 2021). Lastly, the World Bank Group launched *Mental Health for Sustainable Development* which promotes change in policy to bring mental health at the center of global health and development agendas (The Lancet, 2014; Dutta, 2020). Although the COVID‐19 pandemic has slowed or interrupted these programs (CAMH, 2020), there is a need to renew efforts to support youth suffering with depressive and anxiety symptoms and alcohol use.

## 4 OBJECTIVES

The objective of this review is to synthesize the best available evidence on the effectiveness of standalone mobile apps for the reduction of depressive symptoms (depression, generalized anxiety, psychological distress) and alcohol use among youth.

## 5 METHODS

### 5.1 Stakeholder engagement in this review

 

### 5.2 Criteria for considering studies for this review

#### 5.2.1 Types of studies

The methods outlined below are further described in our published protocol (Magwood, 2022). We developed these methods by engaging youth with lived experience of mental health concerns or substance use. Youth were engaged as co‐researchers throughout the review and were critical in the development of the research question, selection of outcomes, and determining the analytic approach. Youth participated in article selection, data extraction, interpretation of findings, and writing the final report. Youth who met authorship criteria are included as co‐authors in this report.

We included randomized control trials (RCTs), including any cluster‐randomized trials or crossover trials. We included only RCTs because randomization is the only way to prevent systematic differences between baseline characteristics of participants in different intervention groups in terms of both known and unknown confounders, and claims about cause and effect can be based on their findings with far more confidence than almost any other type of study (McKenzie, 2023). Based on initial scoping exercises and review of other published systematic reviews, we determined that sufficient evidence from RCTs was available on our topic of interest.

#### 5.2.2 Types of participants

We included studies focusing on youth aged 15–24 years with heightened symptoms of depressive disorders, including depression and anxiety spectrum disorders or problematic alcohol use. We focused our efforts on depression, generalized anxiety and alcohol use, and excluded studies that focused specifically on bipolar disorder, psychotic disorders, eating disorders, and other substance use disorders aside from alcohol. Our age range was selected to coincide with the United Nations definition of youth (UN, n.d.). We did not exclude populations on the basis of gender, socioeconomic status, geographic location or other personal characteristics. If we identified studies with participants with variable age ranges (e.g., high school students aged 12–15 years and young adults aged 25–30 years) we included the study if (1) the mean age of the study participants was between 15 and 24 years, or (2) age‐disaggregated data were available from the authors.

#### 5.2.3 Types of interventions

This systematic review focused on mobile apps that targeted the management of depressive disorders and/or alcohol use disorders. Mental health mobile apps are apps that are available on a mobile device (smartphone, tablet, or phablet), which can be used by the participant without direct intervention from the health care provider. We excluded mental health apps that targeted wellness and prevention of psychological disorders and apps that targeted bipolar disorder, psychosis, post‐traumatic stress disorder, attention‐deficit hyperactivity disorder, substance use disorders (aside from alcohol), and sleep disorders that would vary in design and functionality.

We focused on mobile apps for management of depressive disorders and alcohol use that delivered interventions for youth at any time or place in the absence of a direct interaction with a healthcare provider or specialist. Therefore, we excluded any platforms or social media approach that focussed on prevention or uniquely connected patients with their healthcare provider via video‐conferencing or voice calls, such as telemedicine.

Eligible comparisons included usual care, no intervention, wait‐list control, alternative or controlled mobile applications.

Interventions not eligible for inclusion included:

- Mobile phones for sending Short Message Service (SMS) messages
- Web‐based interventions
- Interventions delivered through social media platforms such as Facebook, Twitter and Instagram
- Interventions delivered through email
- Telemedicine services that only provide direct interaction between patients and their remote healthcare provider via teleconferencing

#### 5.2.4 Types of outcome measures

##### Primary outcomes

We included studies which reported on the following outcomes:

- Symptoms of depression
- Symptoms of anxiety
- Psychological distress
- Alcohol use

These outcomes were selected based on consultations with youth aged 15–24. Youth were provided with a list of possible outcomes, identified by scoping the available published literature. A convenience sample of youth were asked to rank outcomes of interest and then came to consensus about the most important outcomes for analysis. To be eligible for inclusion, studies could measure at least one of these outcomes using validated mental health (depression, anxiety, psychological distress) or alcohol use scales. However, we considered self‐report data if no validated measures were available. Examples of these validated scales include:

- Depression: Beck Depression Inventory (BDI), Depression, Anxiety, Stress Scale (DASS), PHQ‐9, Center for Epidemiological Studies Depression Scale (CES‐D), Depressive Symptomatology Self‐Report (QIDS‐SR)
- Anxiety: General Anxiety Disorder‐7, State‐Trait Anxiety Inventory, Spielberger State‐Trait Anxiety Inventory (STAI), Beck Anxiety Inventory (BAI), Penn State Worry Questionnaire (PSWQ)
- Psychological distress: Perceived stress scale (PSS), Kessler Psychological Distress Scale, Psychological Well‐Being Scale (PWBS)
- Alcohol use: Daily Drinking Questionnaire (DDQ), Problem‐Oriented Screening Instrument for Teenagers (POSIT) Substance Use/Abuse scale, Alcohol Use Disorders Identification Test (AUDIT)

##### Duration of follow‐up

All durations of follow‐up will be eligible for inclusion. We will categorize follow‐up as short, medium or long term based on previous literature as follows:

- Short term: 3 months or less
- Medium term: Between 6 and 12 months follow‐up
- Long term: Longer than 12 months follow up

##### Types of settings

We included studies from any setting.

### 5.3 Search methods for identification of studies

#### 5.3.1 Electronic searches

We developed a search strategy and had it peer‐reviewed by a librarian with expertise in systematic review searches. We searched the following bibliographic databases: MEDLINE (via Ovid), Embase (via Ovid), PsycINFO (via Ovid), CINAHL (via EBSCO), and CENTRAL (via the Cochrane Library). The search was restricted from January 1, 2008, to July 1, 2022. The 2008 start date was selected to coincide with the release of Google Play and Apple's App Store (Apple, 2008). There were no language restrictions. The search used a combination of indexed terms, free text words, and MeSH headings. Complete search strategies for all databases are included in Supporting Information: Appendix 1. Additionally, we screened the included studies of relevant systematic reviews and the reference lists of included RCTs to identify additional RCTs for inclusion. We also searched clinicaltrials.gov and the WHO trial registry for any records of ongoing or published studies not captured by our database search. We also contacted the authors of RCTs with incomplete data and trial registrations to determine if complete published reports were available (Supporting Information: Appendix 2).

#### 5.3.2 Searching other resources

Not applicable.

### 5.4 Data collection and analysis

#### 5.4.1 Selection of studies

Two review authors independently assessed all potential records identified by our search strategy. They screened titles and abstracts and then the full text of relevant records. Before initiating the screening process, two reviewers undertook a screening exercise with a random sample of *n* = 100 records to ensure inter‐reviewer agreement and calibrate the screening strategy through training, as necessary. Inter‐rater agreement was measured using the kappa coefficient (Cohen, 1960), and a kappa statistic of 0.81 or higher was was set as the cut‐off for adequate inter‐reviewer screening reliability (Landis, 1977). The kappa statistic of our initial screening exercise was 0.70, which required calibrating the screening process through holding recurrent training sessions for our team members (*n* = 12) and reaching consensus on records for which disagreements arose. Thereafter, we resolved any disagreements through discussion or, if required, we consulted a third review author.

#### 5.4.2 Data extraction and management

We developed a standardized data extraction sheet. This extraction framework was piloted with a random sample of *n* = 3 included records and revised accordingly in order to ensure the validity of the data extraction form. Two reviewers extracted data in duplicate and independently and compared results afterwards. Any discrepancies in data extraction were resolved by discussion or with the help of a third reviewer.

Reviewers extracted the following variables: (1) Context of the study: geographical, epidemiological, gender, socio‐economic status contextual data; (2) Study methodology: objective, study design, methodological details such as processes for randomization, allocation and blinding, target population, recruitment and sampling procedures, setting, participant eligibility criteria, and participant baseline characteristics; (3) Intervention: name, description, components (e.g., timing, frequency, route of delivery), and details of the comparison intervention; (4) Outcomes: Definitions, instrument and scale interpretation, timing of outcome measures, adverse events, and usability; (5) Results: Participant follow up, binary (dichotomous) data, continuous data, between‐group estimates, and qualitative key findings; and (6) Author conclusions, funding and conflict of interest.

#### 5.4.3 Assessment of risk of bias in included studies

We assessed the risk of bias of all included studies using the Cochrane Risk of Bias 2.0 (RoB 2.0) tool (Sterne, 2019). Two reviewers independently conducted Risk of Bias assessments and disagreements were adjudicated by a third reviewer, independently. We followed the detailed guidance for using the Cochrane Risk of Bias tool available from Cochrane (https://methods.cochrane.org/risk-bias-2) and from the developers via the Risk of Bias tools website (www.riskofbias.info). These guidance documents provided clear algorithms for determining whether assessments should be rated as “low risk,” “some concerns” and “high risk.”

#### 5.4.4 Measures of treatment effect

We categorized effectiveness data based on intervention purpose and selected data based on our specified outcomes. If a study reported multiple measures for the same outcome, we selected the outcome that was most common across studies (e.g., using the same mental health scale) to allow for pooling in meta‐analyses. Outcome data measured using categorical scales were synthesized using odds ratios. Outcome data measured using continuous scales were synthesized as the standardized mean difference at follow‐up. All effect estimates were accompanied by measures of variance (standard deviations) and statistical significance estimates (confidence intervals or *p*‐values) when possible at the a 0.05 level of significance, unless stated otherwise. We calculated measures of variance and statistical significance whenever the authors do not provide them (Borenstein, 2009).

#### 5.4.5 Unit of analysis issues

We assessed the unit of analysis of all the trials to individuals.

#### 5.4.6 Dealing with missing data

We contacted the authors of the included RCTs to identify any additional data. When data was not reported in a manner that allowed synthesis, we highlighted in our results narratively and as reported by authors.

#### 5.4.7 Assessment of heterogeneity

To facilitate the process of assessing for clinical heterogeneity between studies, we standardized the PICO definitions (population, intervention, comparison, and outcome) of each included study with definitions that align with the scope of our review (McKenzie, 2023). One reviewer followed this standardization process for each included study and a second reviewer verified these decisions. Any discrepancies were resolved by reaching a consensus or consulting a third reviewer. The categories we will use to standardize the PICO definitions are presented below.

Studies that measured the same outcome (e.g., severity of depression symptoms) were then assessed for clinical heterogeneity by examining whether their standardized PICO definitions aligned with each other. We categorized interventions as follows: (1) Apps which deliver CBT, (2) apps which deliver mindfulness therapy, (3) apps which deliver a combination of CBT and mindfulness, and apps which deliver other therapy designs. We also identified two types of comparison groups: The first is withheld comparisons where a participant does not receive any intervention (e.g., they may continue with “usual care” or are waitlisted to receive the intervention). In this situation, the participant is not blinded to their intervention assignment. In contrast, “active controls” (or “controlled interventions”) maintain blinding of the participant. In these circumstances, participants are provided with a placebo app which does not deliver a therapy and this is compared to an app which does deliver therapy. In this situation, the findings from these trials offer a lower risk of bias estimate on whether or not apps can deliver a mental health therapy to improve youth mental health.

**Table:** 

PICO	PICO definitions	Standardized PICO categories
Population	Youth selected to have the condition	Youth with mental health condition
	Youth had the condition at baseline	
Intervention	Smartphone application to manage an existing or assumed to exist condition using a specific mental health therapy design (e.g., Cognitive Behavioral Therapy, Mindfulness, self monitoring of symptoms)	Management‐based intervention for each mental health therapy design (i.e., aimed to manage a certain mental health condition using a certain mental health therapy design)
Comparison	Sham intervention	Controlled intervention
	Placebo intervention	
	No intervention	Withheld comparison
	Waitlisting	
	Usual/standard care	
Outcome	Depression symptoms	
	Anxiety symptoms	
	Psychological stress	
	Alcohol use	

Furthermore, we assessed the statistical heterogeneity of meta‐analyzed results by examining the *I* ^2^ and *χ* ^2^ estimates calculated using RevMan 5.3. When we detected significant statistical heterogeneity (Higgins, 2003) we examined and reported its source by conducting sensitivity analyses of studies included in the meta‐analysis.

Study populations were not large enough to allow us to investigate heterogeneity with subgroup analysis considering gender and socioeconomic status.

#### 5.4.8 Assessment of reporting biases

We used the Cochrane ROB 2.0 criteria related to selective outcome reporting to assess for reporting bias. We reported all protocols from our search that did not have a published study. We aimed to assess publication bias with funnel plots with all meta analyses with *n* > 10 studies, but no meta‐analyses met these criteria (Boutron, 2022).

#### 5.4.9 Data synthesis

Whenever clinical homogeneity allowed, we meta‐analyzed results and created forest plots using RevMan 5.4 software using a random‐effects model (Borenstein, 2009). We chose a random‐effects model to account for the inherent heterogeneity between the characteristics of study cohorts, intervention design, and implementation context. Results that were not pooled together were reported narratively (Popay, 2006; Campbell, 2020). All results are accompanied by a GRADE certainty assessment. Furthermore, we ascertained whether our pooled results reached a minimal clinically‐important thresholds of outcome measures using evidence from the scientific literature on outcome measurement tools (Kounali, 2020; Button, 2015; Kroenke, 2001), whenever possible, and interpreting the results with our youth patient partners with lived experience (Weinfurt, 2019).

#### 5.4.10 Subgroup analysis and investigation of heterogeneity

We were unable to conduct any subgroup analysis due to insufficient data.

#### 5.4.11 Sensitivity analysis

We conducted sensitivity analyses when necessary, exploring the clinical variability of studies that contributed to heterogeneity.

#### 5.4.12 Summary of findings and assessment of the certainty of the evidence

We assessed the certainty of the evidence using the GRADE approach.

## 6 RESULTS

### 6.1 Description of studies

#### 6.1.1 Results of the search

**Figure 2:**
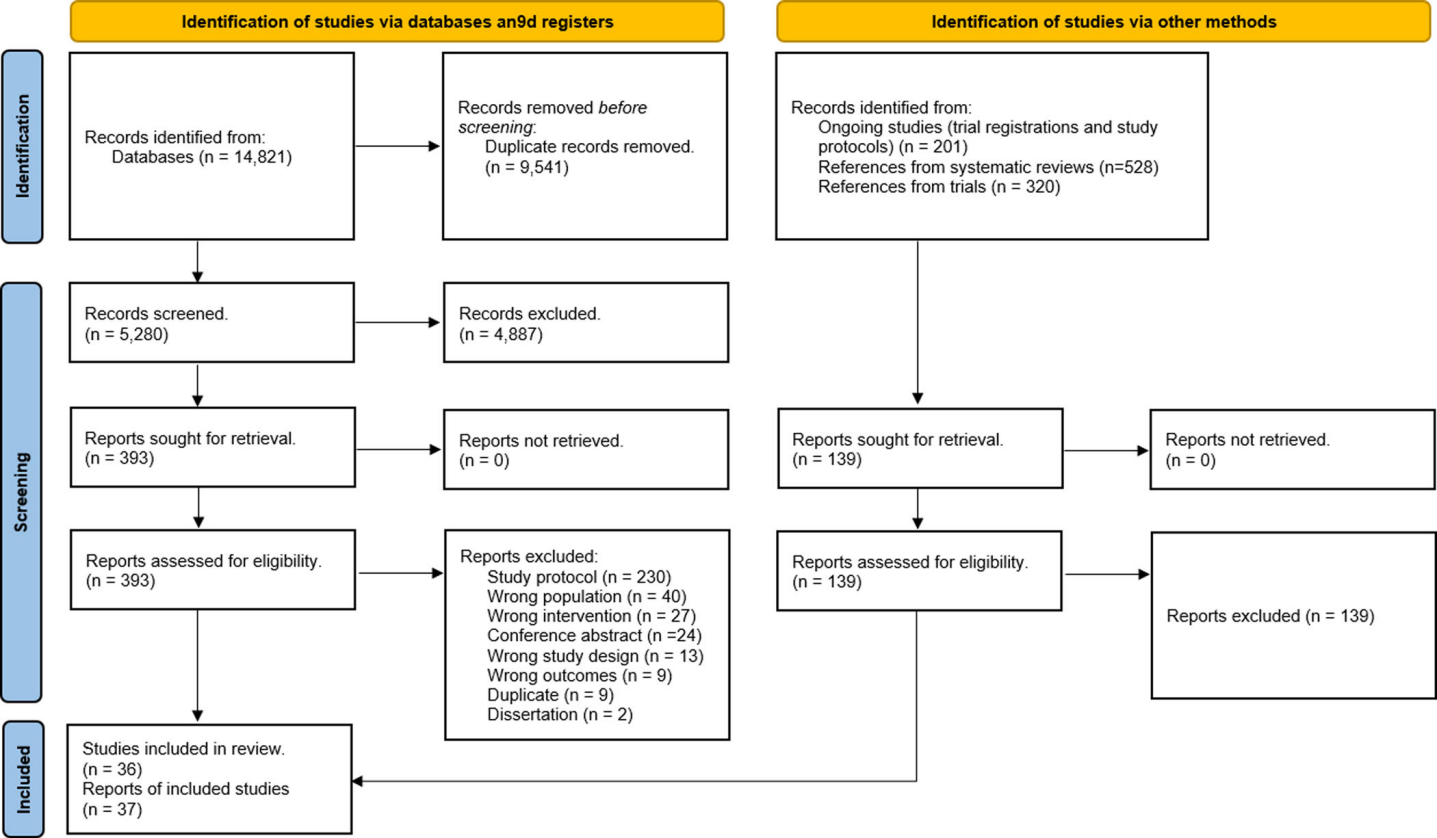
PRISMA flow diagram.

Our search identified 5280 citations. After screening titles and abstracts, 393 citations were retained for full text review. Of these, 355 citations did not meet eligibility criteria and 36 trials reported in 37 publications were included in the final review (Figure 2).

#### 6.1.2 Included studies

We included 36 RCTs (7984 participants) published in English and conducted in 15 different countries, as follows: USA (*n* = 13), Australia (*n* = 5), New Zealand (*n* = 3), China (*n* = 2), South Korea (*n* = 2), UK (*n* = 2) and one RCT from each of Canada, Finland, Germany, Iceland, Ireland, Japan, the Netherlands, Spain, Sweden, and Taiwan. The majority of included studies were conducted among male and female students in university settings. Only one study focused exclusively on women (Levin, 2020). Three studies included participants of diverse gender identity (Cordova, 2020; Bruhns, 2021; Thabrew, 2022) and one study reported a majority of participants identifying as LGBTQI (Torok, 2022). One study focused specifically on youth experiencing homelessness (Thompson, 2020). Interventions varied in mental health therapy design and functionality. The most common therapy designs employed mindfulness, CBT, or a combination of the two. However, other interventions also included self‐monitoring (Reid, 2011), medication reminders (Hammonds, 2015), cognitive bias modification or positive stimulation (Teng, 2019; Visser, 2020; Kageyama, 2021), DBT (Torok, 2022), gamified health promotion (Egilsson, 2021), or social skill building to address loneliness (Bruehlman‐Senecal, 2020). A summary of the characteristics of all included studies is available in Table 1.

**Table 1:** Characteristics of included studies.

Study ID	Design	Aim	Setting	Participants	Intervention	Comparison	Outcome assessment
Boyle (2017)	Randomized Controlled Trial	(1) To evaluate the ability of a gamified personalized normative feedback (PNF) alcohol intervention, compared to a non‐gamified PNF in reducing short‐term alcohol use.	Psychology Department at Loyola Marymount University, California, USA	*n* = 237 Gender: 75.5% female, 24.% male Ethnicity: 48% Caucasian, 18.5% Asian; 16% Hispanic/Latino Other: College Students	CampusGANDR Application: The app delivers the same components of PNF, but in a more engaging way. Session topics focused on alcohol, social media, and TV. The app is informed by both gamification and alcohol intervention research.	Control group: received a survey asking participants to answer questions about their perceptions of the drinking behaviors of typical students of their same sex and class year and then reported their own alcohol use.	Perceived Descriptive Drinking Norms and Drinking Behavior: self report of the maximum drinks consumed on a single night so far during the semester, number of times participants had partied during the past week and number of drinks consumed during the previous weekend
Bruehlman‐Senecal (2020)	Randomized Controlled Trial	(1) To examine the initial efficacy, feasibility, and desirability of the smartphone app, Nod, designed to deliver cognitive and behavioral skill‐building exercises to reduce loneliness during the transition to college.	Public universities in Northwestern, USA	*n* = 221 Mean Age: 18.68 Gender: 59.3% female, 36.7% male Ethnicity: 52.9% White, 13.6% Latino, 9.5% Asian, 3.6% Black, 0.9% Native American, 0.9% Hawaiian or Pacific Islander, 18.6% two or more races/ethnicities).	Nod Application: a. Mobile app that addresses loneliness in first year college students by using positive psychology, mindfulness‐based self‐compassion, and cognitive behavioral skill‐building exercises.	Control group: delayed access to the app	Loneliness: 8‐item version of the UCLA loneliness 182 questionnaire Anxiety and Depression symptoms: measured with 7‐item Generalized Anxiety Disorder Scale and the Patient Health Questionnaire Social Anxiety Symptoms: measured with 3‐item Mini Social Phobia Inventory Sleep Quality: measured with Pittsburgh sleep quality index Perceived Social Support: measured with 3‐item support subscale of the Comprehensive Inventory for Thriving
Bruhns (2021)	Randomized Controlled Trial	(1) To investigate the effectiveness, acceptance, and side effects of a self‐help smartphone app. (2) To examine the influence of treatment expectations and attitudes toward internet‐ and mobile‐based interventions on treatment adherence and effectiveness.	Universities in Germany	*n* = 423 Mean age: 22.98. Gender: 10.8% men, 88.5% women, 0.8% diverse gender Other: University students with depressive symptoms (PHQ‐9 > 0). Students with suicidal tendencies or current/past bipolar or psychotic disorder were excluded.	“MCT & More”: a smartphone app, primarily intended for individuals with depressive symptoms. The basic package comprises 57 short exercises based on group metacognitive training (MCT). Topics include: cognitive strategies, communication and interaction, positive activities, mindfulness and imagination, gambling, and metacognitive training. In addition, the app contains gamification elements. Depending on the number of exercises completed, users can collect bronze, silver, or gold medals and obtain an open umbrella as a symbol for long‐term protection.	Control group: Wait list control	Depression Module: (PHQ‐9) 9‐items on a 4‐point rating scale. Total score between 0 and 27. Self‐esteem: (RSE) Rated on a 4‐point rating scale, total score ranges from 10 to 40 points. Quality of Life: (WHOQOL‐BREF) 26 items (4 domains) Rated on a 5‐point scale. Only the first question was used. Online Intervention Attitude: (APOI) Consists of 4 dimensions, 16 items and can be rated on a 5‐point rating scale. Total ranges from 16 to 80. Therapy Expectation: (PATHEV) 10 Items, cover 3 subscale, 5‐point rating scale, total scale ranges from 11 to 55. Patient Satisfaction: (ZUF‐8) 8 items, rated on a 4‐point scale, a total score of 8–32. Negative Effects of Psychotherapy: (INEP) 21 items, consists of 2 scales. on a 7‐point scale, items 16–21 were excluded from the assessment.
Cordova (2020)	Randomized Controlled Trial	(1) To determine the feasibility of Storytelling 4 Empowerment (S4E), compared to enhanced usual practices in effecting clinician‐youth risk communication, prevention knowledge, and substance use and sexual risk refusal self‐efficacy.	Youth centered community health clinic located in Southeast Michigan, USA	*n* = 50 Mean Age: 18.82 Gender: 82% female, 8% males, 8% transmales, and 1% non‐disclosed Ethnicity: 46% Non‐Hispanic White, 42% Black, 2% Native American, 8% ascribing to more than one race, and 2% selecting Other.	S4E Application: Youth would receive targeted, tailored prevention content based on their responses to the S4E risk behavior assessment. This assessment is intended to identify the youths' specific risk behaviors based on the past year and lifetime reports of substance use, sexual risk behaviors, and past 6‐month STI/HIV testing practices.	Control group: enhanced Usual Practice Group: consists of the clinic's usual servcices including primary care, mental health, sexual and reproductive health, substance use prevention, support, and education.	Substance Use Refusal Skills: assessed through 2 separate items on a 4‐point scale Substance Use Prevention Knowledge: assessed through 2 separate items. Responses were on a 4‐point agreement scale Substance Use Behaviors: assessed using items adapted from the Monitoring the Future study (dichotomous yes/no)
Earle (2018)	Randomized Controlled Trial	(1) To assess the feasibility of enrolling students in a gamified intervention outside of the controlled laboratory setting (2) Examine the degree to which the two types of drinking feedback delivered within the game were effective in reducing normative beliefs and alcohol use.	An unidentified private, mid‐sized university on the West coast of the USA	*n* = 276 Age: >18 years old Gender: 55% female, 45% male Ethnicity: 47% Caucasian, 20% Hispanic/Latino, 14% Asian, 12% African American, and 7% were Multiracial or Other. Other: University Student	CampusGANDR (v1) Application: a gamified app that tests students' perceptions of various college life topics with a system of points. Each question contained two parts. First, participants estimated how the average same‐sex student in their class would answer. Next, students reported their own answer. Participants would later have access to a feedback module, that would allow participants' misperceptions to be corrected and participants were shown how their behavior compared to that of their peers.	Control group: participated in the same gamified app as those in the intervention group however, they only received non‐alcohol related questions and did not receive any feedback on questions related to alcohol related issues.	Self‐Report questions on: (1) the maximum drinks consumed on a single night so far during the semester (2) number of times participants had partied during the past week (3) number of drinks consumed during the previous weekend.
Egilsson (2021)	Randomized Controlled Trial	(1) To assess time‐specific attrition rates among adolescents in an mHealth intervention, and to describe the intervention's usage and feasibility in relation to adolescent self‐efficacy levels, and emotional and physical health.	Elementary schools in Reykjavík, Iceland	*n* = 41 Mean Age: 15.6 Gender: 41.5% female, 58.5% male Other Criteria: attending public school	SidekickHealth Application: an app centered on helping the user set goals and create health‐related missions (gamification of tasks in three main categories: food and drink, physical activity, and mental health.	Control group: waitlist control	Self‐efficacy: measured with 10‐item General Self Efficacy Scale BMI: measured with BMI index reference values for Swedish children adjusted for age and sex Depressive symptoms: measured with Children's Depression Inventory Anxiety Symptoms: measured with Multidimensional Anxiety Scale Sleep Problems: measured wtih BEARS sleep screening algorithm The amount, frequency, and time of daily physical activity: measured through in‐app activity Self‐Reported Stress Levels
Fish (2019)	Randomized Controlled Trial	(1) To assess if the prescription of a gamified mindfulness meditation application would decrease college students' reported symptoms of depression.	Universities in North Carolina, USA	n = 91 Mean Age: 21 Gender: 96% female, 4% male Other Criteria: University student	Headspace Application: an app that educates users how to perform mindfulness meditation, provides guided meditations, and provides free access to 10 min meditation sessions over 14 days.	Control: continue with their usual routine and do not participate in any meditation practices during the 2‐week period of the study.	Depression Severity: 9‐item 4‐point scale Patient Health Questionnaire
Fitzpatrick (2017)	Randomized Controlled Trial	(1) To assess the feasibility of delivering CBT in a conversational interface via an automated bot in reducing symptoms of depression and anxiety.	An unidentified University in New York, USA	*n* = 70 Mean Age: 22.05 Gender: 81% female, 19% male Ethnicity: 79% Caucasian, 7% Asian, 9% more than one race, 2% African American, 2% Native American/Alaskan Native Other: College students and young adults who self‐identify as having symptoms of anxiety and depression	Woebot Application: an automated conversational agent designed to deliver CBT in the format of brief, daily conversations and mood tracking. Woebot is used within an instant messenger app that is platform agnostic and can be used either on a desktop or mobile device.	Control group: participants were directed to the NIMH resources section and specifically, a free publication entitled “Depression in College Students. The eBook provided comprehensive evidence‐based information on depression among college students including sections on signs and symptoms, different types of treatments, answers to frequently asked questions, and a list of resources including further reading, helpline numbers, and other resources.	Frequency and severity of depressive symptomatology: The Patient Health Questionnaire PHQ‐9 (score from 0 to 20 with higher scores indicating more severity) Frequency and severity of anxious thoughts and behaviors: The Generalized Anxiety Disorder GAD‐7 (score from 0 to 21 with higher scores indicating more severity) Positive and negative effects (emotions): The Positive and Negative Affect Schedule PANAS (score from 10 to 50 with higher scores indicating more extreme affects) Acceptability (mixed methods): a 5‐point Likert scale assessing overall satisfaction, satisfaction with content, extent of emotional awareness, and learning experience, as well as comments about experiences with the application
Flett (2020)	Two‐Arm Randomized Controlled Trial	(1) To examine whether access to a mindfulness meditation app would be associated with improvements in psychological distress. (2) To examine whether there would be a dose‐response relationship between app use and psychological distress.	The University of Otago, Dunedin, New Zealand	*n* = 250 Mean Age: 17.87 Gender: 73.6% female, 26.4% male Ethnicity: 78.6% New Zealand European descent	Headspace Application; Headspace is a mindfulness meditation application that provides guided and unguided mindfulness meditations. Headspace uses a variety of formal meditation practices such as, mindful breathing, body scan, sitting meditation, practice of non‐judgment of thoughts and emotions, and other guided meditations that vary in orientation.	Control group: waitlist control – to receive Headspace during the second semester.	Psychological distress: assessed using the 10‐item Kessler Psychological Distress Scale College adjustment; assessed using the 19‐item College Adjustment Test
Flett (2019)	Three‐Arm Randomized Controlled Trial	(1) To test the effect of two mobile mindfulness apps on changes in mental health, relative to a control app.	The University of Otago, Dunedin, New Zealand	*n* = 210 Mean Age: 20.08 Ethnicity: 73.6% New Zealand European descent, 12.0% Asian; 5.8% Māori or Pacific Islander; 8.6% other Other criteria: Undergraduate students	(1) Headspace Application; Headspace is a mindfulness meditation application that provides guided and unguided mindfulness meditations. Headspace uses a variety of formal meditation practices such as, mindful breathing, body scan, sitting meditation, practice of non‐judgment of thoughts and emotions, and other guided meditations that vary in orientation. (2) Smiling Mind Application: app offers guided and unguided mindfulness meditation practices	Control group: Evernote (the attention placebo control application) is an app that has a note‐taking function to “jot down all the things you can remember doing on this day last week” for 10 min every day.	Depressive symptoms: assessed using the 20‐item Center for Epidemiological Studies Depression Scale Anxiety: assessed using the Hospital Anxiety and Depression Scale–Anxiety Subscale Stress: assessed using the 10‐item Perceived Stress Scale
Franklin (2016)	Randomized Controlled Trial	(1) To test whether using a Therapeutic Evaluative Conditioning (TEC) app would display reductions in self‐cutting and overall non‐suicidal self injury. (2) To examine the effect of active TEC dosage on self‐injurious thoughts/behaviors and whether any treatment effects persisted during the month after TEC access ended.	Recruitment, allocation, and assessment took place online, USA	*n* = 408 Mean Age: 22.91 Ethnicity: 83.21% Caucasian, 5.34% Asian, 3.82% Hispanic, 1.53% Native American, 6.12% other Other criteria: Individuals with a recent history of frequent non‐suicidal self injury and severe histories of self‐injurious thoughts and behaviors	TEC Application: a brief, game‐like treatment that could be accessed by any device with an Internet connection. It takes 1 to 2 min to complete a single instance of TEC and TEC becomes more challenging as the trials progress.	Control group: received access to the control TEC	Presence, frequency, and characteristics of self‐injurious thoughts and behaviors: measured using the Self‐Injurious Thoughts and Behaviors Interview Emotional reactivity: measured using the Emotion Reactivity Scale Psychological distress: measured using Brief Symptom Inventory (BSI) Engagement in dysregulated behaviors: measured using the Index of Dysregulated Behaviors Implicit aversion to Non‐suicidal self injury behaviors: measured using the Affect Misattribution Procedure
Gajecki (2014)	Three‐Arm Randomized Controlled Trial	(1) To investigate the effects of two smartphone apps that use real‐time estimated blood alcohol concentration in targeting drinking choices on party occasions and reducing problematic alcohol intake.	Stockholm University and the Royal Institute of Technology in Stockholm, Sweden	*n* = 1932 Mean Age: 24.72 Gender: 51.7% female, 48.3% male Other: University students with risky and harmful alcohol drinking behaviors	(1) Promillekoll Application: The user can register his/her alcohol consumption in real time, where the app displays the user's current eBAC. The application also offers a number of specific strategies to maintain alcohol consumption at a level that is not harmful—in this case, 0.06 percent BAC. The application warns the user if the drink entered will result in an eBAC over 0.06 percent and only displays values up to 0.08 percent. It also provides information texts on alcohol and BAC. (2) Party planner Application; This app has a functionality of simulating or planning a drinking event beforehand and then comparing the simulation to the real‐time event afterwards. The app user would then be able to pace his or her drinking based on a more realistic view of the amount of alcohol actually corresponding to a certain eBAC level.	Control group: did not receive any intervention or feedback on risky drinking.	Quantity and frequency of alcohol consumption: assessed using the Daily Drinking Questionnaire (DDQ) Estimated blood alcohol concentration (eBAC): calculated based on the values from the Daily Drinking Questionnaire in conjunction with weight and gender for each individual. Alcohol consumption and signs of harm and dependence in relation to alcohol: measured using the AUDIT scale
Hammonds (2015)	Randomized Controlled Trial	(1) To determine if the use of an electronic medication reminder app is effective for increasing adherence to antidepressant medications in depressed college students.	Unidentified state‐funded institution in Ohio, USA	*n* = 57 Mean Age: 20.6 Gender: 86% female, 14% male Ethnicity: 98.2% Caucasian Other Criteria: College students who were prescribed an antidepressant medication	Electronic medication reminder Application: Participants were required to use the medication reminder app to indicate when they had taken their medication by responding to the message received.	Control group: participants were instructed to continue taking their medications as prescribed by their physician, but did not use the reminder app.	Depressive symptoms: assessed with the Beck Depression Inventory Perceived Stress: assessed with the Perceived Stress Scale
Hides (2018)	Randomized Controlled Trial	(1) To determine the 1‐month efficacy and 2, 3 and 6 months outcomes of a mobile app called Ray's Night Out app, in increasing alcohol knowledge and reducing alcohol use in young people.	Unidentified university in Australia	*n* = 197 Mean Age: 20.4 Gender: 77.7% female, 22.3% male Other Criteria: Australian residents who drank alcohol at least monthly	Ray's Night Out Application: the app invites young people to take Ray, a red panda avatar, on a “relaxed” “fun” or “crazy” virtual night out. It aims to provide users with the information, motivation and behavioral skills to set a drinking goal for the night by keeping Ray below a line for drinking. To increase motivation to take good care of Ray, users receive “good vibe” points, which unlock photo booth rewards for using protective behavioral strategies.	Control group: Delayed access‐participants received a 1‐month delayed access to the *Ray's Night Out* app.	Alcohol knowledge: measured using items adapted from CLIMATE Schools studies and the School Health and Alcohol Harm Reduction Project “Patterns of Alcohol” index Frequency of risky single occasion drinking: measured using a standard questionnaire Typical number of standard drinking units consumed on one occasion: measured using a standard questionnaire Maximum quantity of standard drinking units consumed on one occasion: measured using a standard questionnaire Frequency of alcohol‐related problems: measured using the Rutgers Alcohol Problem Index Harmful or problematic alcohol use: measured using the Alcohol Use Disorders Identification Test
Hides (2019)	Randomized Controlled Trial	(1) To evaluate an app called Music eScape, in assisting young people with identifying, expressing, and managing emotions.	Two large unidentified universities in Australia	*n* = 169 Mean Age: 19.9 Gender: 79.3% females, 20.7% males Other Criteria: Australian residents, who reported at least mild distress in the past month	Music eScape Application: the app allows users to create a moodmap of the music stored on a user's phone by tagging each song with a mood. Users then have the opportunity to review the tags assigned by the app. Music eScape then creates playlists for different moods and situations, such as “cheer up,” “wake up,” and “focus.” After completing their mood journey, users are asked to reflect on their current mood and rate the effectiveness of the playlist they just experienced	Control group: Waitlist control – 1 month delayed access to App; Comparison therefore results prior to using app.	Emotion regulation: measured with the short‐form of the Difficulties in Emotion Regulation Scale Psychological distress: measured with The K10 scale Mental well‐being: measured with the Mental Health Continuum‐Short Form
Huberty (2019)	Randomized Controlled Trial	(1) To test the efficacy of mindfulness meditation intervention delivered via a mobile app on stress levels in college students.	Arizona State University, in Southwestern USA	*n* = 109 Mean Age: 20.41 Gender: 88% female, 12% male Ethnicity: 59% Caucasian, 41% Non‐Caucasian Other Criteria: Full‐time undergraduate students who self‐reported stress	Calm mobile Application: mindfulness meditation mobile app that offers a range of mindfulness meditation practice guide modules that vary in length, instruction, and content. Calm also integrates some CBT techniques into the meditation sessions on occasion.	Control group: Wait‐list control received access to the Calm app after 12 weeks. They were also asked not to participate in any mindfulness activities (eg, yoga, meditation, and qigong) during this time.	Perceived stress: measured with PSS scale Mindfulness: measured with Five Factor Mindfulness Questionnaire. Self compassion: measured with Self‐Compassion Survey Short‐Form
Hur (2018)	Randomized Controlled Trial	(1) To examine the effect of an app‐based CBT program in reducing clinical symptoms. (2) To assess whether this scenario‐based platform results in modifying the users' dysfunctional beliefs through reappraisal and re‐constructing.	Two unidentified universities in Seoul, Korea	*n* = 48 Mean Age: 23.71 Gender: 88.3% female, 11.7% male Other Criteria: DSM‐5 diagnosis for Other Specified Depressive Disorder	Todac Todac mobile cognitive behavioral therapy (CBT) Application: The app was developed with focus on targeting dysfunctional beliefs in individuals at high risk of psychiatric illness or with psychiatric disorders. Users were presented with scenarios following 3 steps: Step I: identifying, Step II: “Decatastrophizing,” Step III: “Distancing.” After each step, users can assess others' responses and advice.	Control group: Mood chart, which required participants to complete a daily mood diary, recording their mood state (e.g., depressed, manic) and sleep quality/quantity.	Dysfunctional attitude scale: measured with Dysfunctional Attitude Scale Depressive symptoms: measured with Beck Depression Inventory Scale State‐trait anxiety: measured with STAI‐X2 (State‐Trait Anxiety Inventory) Self‐esteem: measured with Rosenberg Self‐esteem Scale
Kageyama (2021)	Randomized Controlled Trial	(1) To assess the preliminary efficacy of the Subliminal Priming with Supraliminal Reward Stimulation, a smart phone application intervention for people with subthreshold depression.	Kibi International University in Okayama Prefecture, Japan	*n* = 32 Mean Age: 20.1 Gender: 34.4% female, 65.6% male Other Criteria: subthreshold depression	SPSRS Application: an app designed to improve depressive symptoms in people with subthreshold depression by presenting positive word stimuli through videos. The app is also programmed to display videos that feature general confidence‐boosting words.	Control group: waitlist, non‐interventional and did not receive access to the SPSRS application during the study period.	Depressive Symptoms: measured with 20‐item Center for Epidemiologic Studies Depression Scale Psychological Distress: measured with 6‐item Kessler Screening Scale for Psychological Distress
Kazemi (2020)	Randomized Controlled Trial	(1) To examine the efficacy of a theoretically based mHealth app for alcohol intervention.	Large public University in the Southeastern USA	*n* = 379 Mean Age: 18.94 Gender: 40% female, 60% male Ethnicity: 79% Caucasian, 21% Non‐Caucasian Other Criteria: screen positive for alcohol use in the last month	BMI + SP Application: the app incorporated many features aimed at motivating users to change drinking habits via interactive tools. The app collected data, tracked behavior, provided education, and offered incentives for behavioral changes. Key features included: My Coach, Personalized Feedback, Strategies, Know Your BAC, Daily Log, Learn More, and Where to Go. The app also sent messages to participants aimed at behavior change, provided personalized normative feedback, and offered strategies to promote healthy drinking behavior.	Control group: received assessment only and no intervention	Alcohol consumption: measured with (1) alcohol use disorders identification test (AUDIT) 10‐item questionnaire (2) daily drinking questionnaire (3) young adult alcohol consequences questionnaire Motivation to Change: measured with readiness to change questionnaire
Kenny (2020)	Randomized Controlled Trial	(1) To test the effectiveness of CopeSmart in improving self‐management through emotional self‐monitoring and the use of positive coping strategies.	Elementary, secondary schools and individual homes in Dublin Mid‐Leinster Health Service Executive Region, Ireland	*n* = 560 Mean Age: 16.05 Gender: 62% female, 38% male Ethnicity: 96% Caucasian, 4% Other	CopeSmart Application: a mental health mobile app which promotes self‐management through emotional self‐monitoring and the use of positive coping strategies. Allows users to rate how happy, angry, sad, stressed or worried they have felt on a scale of 1–10.	Control group: no intervention	Emotional Self Awareness (ESA): measured with The Emotional Self‐Awareness Scale Psychological Distress: measured with the Depression Anxiety and Stress Scale‐21
Lee (2018)	Randomized Controlled Trial	(1) To evaluate a mindfulness‐based app called DeStressify, in reducing stress, anxiety, depressive symptomatology, sleep behavior, and quality of life among university students.	University of British Columbia in Kelowna, BC, Canada	*n* = 206 Mean Age: 20.3 Gender: 58% female, 42% male Ethnicity: 65% Caucasian, 16% Chinese 12% South Asian Other Criteria: Full‐time undergraduate student	DeStressify Application: The app contains a core plan that delivers mindfulness‐based exercises through audio, video, or text files.	Control group: waitlist, delayed access – no treatment and no intervention material until after the post intervention survey was completed.	Perceived Stress: measured with Perceived stress scale Anxiety: measured with State‐Trait Anxiety Inventory for adults Depressive Symptoms: measured with The Quick Inventory of Depressive Symptomatology Self‐Report Sleep Quality: measured with Pittsburgh Sleep Quality Index Work Productivity: measured with Work Productivity and Activity Impairment Questionnaire
Levin (2020)	Randomized Controlled Trial	(1) To evaluate the feasibility and acceptability of a popular mindfulness meditation app, called Stop, Breathe & Think for students on reducing psychological distress.	Unidentified University in the Mountain West Region of the USA	*n* = 23 Mean Age: 20.43 Gender: 100% female Ethnicity: 87% Non‐Hispanic, White, 9% Hispanic, White, 4% American Indian	Stop, Breathe & Think Application: The app provides guided meditations and mindfulness procedures.	Control group: waitlist – participants did not receive additional resources and did not have access to the application for the duration of study.	Mental Health Symptoms: measured with Counseling center assessment of psychological symptoms – 34 item version Mindfulness: measured with Five facet mindfulness questionnaire
Liu (2022)	Unblinded randomized controlled trial	To examine the superiority of a newly developed chatbot‐delivered self‐help depression intervention to a minimal level of bibliotherapy regarding (1) efficacy on depression symptoms reduction, (2) adherence, and (3) therapeutic alliance.	Universities in the cities of Harbin, Wuhan, and Guangzhou, China	*n* = 83 Mean age: 23.08, SD = 1.76, range 19–28 Gender: 55% (46/83) female Language: All native Chinese speakers Other: University students (undergraduate or postgraduate)	“XiaoNan” via WeChat: A therapy chatbot (XiaoNan) was developed and deployed through the smartphone app “WeChat.” XiaoNan is a pipeline‐based chatbot powered by the open‐source conversational AI “RASA.” Therapeutic content and conversations were based on machine learning models. Chatbot responses were created according to the principles of CBT.	Control group: Bibliotherapy, referring to the treatment method that uses literature to alleviate a patient's problems. In bibliotherapy, patients receive psychological intervention by reading literature following the advice of professionals.	Depression: (PHQ‐9) 9‐items on a 4‐point rating scale. Total score between 0 and 27. Anxiety: (GAD‐7) 7‐item on a 0 to 3 point scale. Total score between 0 and 21. Positive and Negative Affect Symptoms: (PANAS) Two 10‐item scales rated on a 5‐point scale
McCloud (2020)	Unblinded randomized controlled trial	(1) To evaluate the effectiveness of a self‐guided mobile app, Feel Stress Free, for the treatment of depression and anxiety symptoms in students.	Universities in England including: University College London, School of Oriental and African Studies University of London, University of Buckingham and University of Roehampton, UK	*n* = 168 Mean Age: 24.3 Gender: 82.7% female, 17.3% male Other Criteria: scored 8 or above on one or both subscales of the Hospital Anxiety and Depression Scale, indicating at least a possible case of depression and/or anxiety	The Feel Stress Free Application: the app, uses CBT‐based activities and comprises 4 behavioral relaxation activities: calm breathing, mindfulness‐style meditation, deep muscle relaxation, and self‐hypnosis; one cognitive activity, incorporating both mood tracking and thought challenging; a relaxing minigame; and a feature for positive messages in a bottle.	Control group: waitlist control	Depressive symptoms: HADS‐Depression Subscale Anxiety: HADS‐Anxiety Subscale
Newman (2021)	Randomized Controlled Trial	(1) To conduct a pilot test of a smartphone‐based guided self‐help intervention for Generalized Anxiety Disorder.	Penn State University and Stanford University in the USA	*n* = 100 Mean Age: 21.62 Gender: 82% female, 18% male Ethnicity: 68% Caucasian/White, 4% Arab/Middle Eastern/Arab American, 16% Hispanic/Latino, 20% Asian/Asian American, 2% Asian Indian, 2% Pacific Islander Other criteria: self‐reported generalized anxiety disorder	CBT intervention Application: the app had units that covered an introduction to anxiety, automatic thoughts, cognitive reframing, introduction to behavior change, imaginal exposure, situational exposure, mindfulness, and habit formation. Each session included psychoeducational lessons (e.g., information about logical errors), tools for skill practice (e.g., identifying one's own logical errors), and regular anxiety check‐ins. Participants also had access to coaches, whose role included supporting and enhancing user motivation, monitoring progress, facilitating goal setting and offering accountability, providing feedback on technique usage. Coach messaging was done via a web‐based “dashboard,” and delivered to users within the mobile application.	Control group: no treatment or access to application	Anxiety: Generalized Anxiety Disorder Questionnaire for DSM‐IV; The State‐Trait Anxiety Inventory‐Trait Version; Penn State Worry Questionnaire Depression, Anxiety and Stress: Depression, Anxiety and Stress Scales‐Short Form
O'Donnel (2019)	Randomized Control Trial	(1) To evaluate the benefits and feasibility of a personalized alcohol harm minimization intervention delivered via smartphones.	Large metropolitan university campus in Australia	*n* = 45 Mean Age: 21.36 Gender: 72% female, 28% male Other Criteria: Reported being motivated to reduce alcohol use, and consumed alcohol, on average, at least once a week.	“Minimise” Application: App delivers protective behavioral strategies tailored to the users' goals and drinking context	“InstantSurvey” Application: App comprises of alcohol self‐monitoring functions, but does not provide feedback or protective behavioral strategies	Alcohol consumption: measured with scales (1) examining consumption of alcohol (2) difficulties with work and/or study due to drinking (3) interpersonal difficulties due to drinking, and (4) physical health related to drinking
Orosa‐Duarte (2021)	Randomized Control Trial	(1) To compare the effect of a mindfulness‐based mobile application versus an in‐person mindfulness‐based training program in terms of reducing anxiety and increasing empathy, self‐compassion, and mindfulness in a population of healthcare students.	Autonomous University of Madrid, Madrid, Spain	*n* = 154 Mean Age: 23 Gender: 85% female, 15% male Other Criteria: Medicine, Psychology, Nursing, or Nutrition students and have no previous training in standardised mindfulness programs	“REM Volver a casa” (“Mindfulness‐Based Emotion Regulation‐Going Home”) Application: App is a training program with eight stages divided into three sections called Listening, Practicing, and Integration into everyday life. The app provides short videos with explanations about the fundamentals of mindfulness, self‐compassion, and the physiological stress reaction, as well as audio segments that guide practices of mindfulness.	IMBP group: in‐person mindfulness program with group sessions and teachers encouraging practice Control group: no intervention	Anxiety: State‐Trait Anxiety Inventory Empathy: Jefferson Scale of Physician Empathy Self‐Compassion: Self‐compassion scale Mindfulness: Five Facet Mindfulness Questionnaire
Ponzo (2020)	Randomized Control Trial	(1) To test the efficacy and sustained effects of using a mobile app (BioBase) and paired wearable device on anxiety and well‐being in university students with elevated levels of anxiety and stress.	Universities in the United Kingdom	*n* = 262 Mean Age: 19.8 Gender: 63% female, 37% male Other Criteria: scored >14 points on the Depression, Anxiety and Stress Scale or >7 points on the DASS‐21 anxiety subscale	The BioBase Application: an app comprising psychoeducational content on mental health and well‐being, mood tracking (via an ecological momentary assessment), and in‐the‐moment exercises (eg, deep breathing and relaxation techniques).	Control group: Waitlist control	Anxiety: State‐Trait Anxiety Inventory; Depression, Anxiety and Stress Scale‐21 items Well‐being and psychological functioning: Warwick‐Edinburgh Mental Well‐Being Scale Depression: DASS‐21, Patient Health Questionnaire
Kauer (2012); Reid (2011)	Randomized Controlled Trial	(1) To investigate the utility and effect of the mobiletype app on depression, anxiety and stress.	Primary care practices in greater Melbourne, Albury‐Wodonga and Goulburn, Australia	*n* = 188 Mean Age: 18.5 Gender: 78% female, 22% male Ethnicity: Other Criteria: diagnosed with mild‐severe emotional health issues as assessed by their GP or indicated by a score greater than 16 on the Kessler Psychological Distress Scale.	Mobiletype (V4) Application: the mobiletype app monitors a young person's mood, stress, coping strategies and daily activities a number of times per day, and their eating, sleeping, exercise patterns, and alcohol and cannabis use once per day. This information is then uploaded to General Physicians, via a secure website and displayed in summary reports for review.	Control group: monitored themselves using an abbreviated version of the mobiletype program that assessed only current activities, location, companions, quality and quantity of sleep, and quantity and type of exercise, and diet. The modules pertaining to mental health and substance use were removed.	Depression, Anxiety, and Stress: Measured with Depression, Anxiety, Stress Scale Emotional Self Awareness: measured by adapting the 20‐item self reflection and Insight Scale, the 10‐item Ruminative Response Scale, and the 12‐item Meta‐Evaluation Scale.
Sun (2022)	Randomized Controlled Trial	(1) To examine the effectiveness of a mindfulness‐based mHealth intervention in reducing symptoms of anxiety and depression for young adults in quarantine compared to a rigorous active control (social support mHealth).	Universities in China	*n* = 114 Mean Age: 22.21 Gender: 73.7% female Other Criteria: Chinese; university students; age 18 or older; can read, speak, and write Mandarin Chinese; in quarantine; have internet access; have access to a smartphone; experiencing depression or anxiety symptoms	A 4‐week mindfulness‐based mobile health intervention, “Mindfulness for Growth and Resilience,” was developed for this study	Rigorous, active, time‐ and attention‐matched social support‐based mHealth condition	Anxiety: measured with Generalized Anxiety Disorder‐7 (GAD‐7; Chinese version) Depression: Patient Health Questionnaire‐9 (PHQ‐9; Chinese version) Mindfulness: Mindful Attention Awareness Scale (MAAS; Chinese version) Perceived Social Support: Multidimensional Scale of Perceived Social Support (MSPSS; Chinese version)
Teng (2019)	Double‐Blind, Randomized Controlled Trial	(1) Assess the effect of home‐delivered attentional bias modification app on symptoms in patients with generalized anxiety disorder.	The National Chung‐Cheng University, in Chia‐Yi, Taiwan	*n* = 93 Mean Age: 21.47 Gender: 74% female, 26% male Other Criteria: Sub‐clinical generalized anxiety disorder	Home‐delivered attention bias modification Application: the app would display a fixation cross, after which a pair of stimulus words written in traditional Chinese characters (i.e., a threatening word and a natural word) were presented on the left and right sides of the now‐missing fixation. A target probe (displayed with an E) then replaced one of the word pairs after it disappeared. The probe was set to replace the neutral stimulus word. The participants were instructed to tap the location of the target probe (E) on the screen with their thumbs	Placebo group: the probe was set to randomly replace the neutral or threatening word only. Control group: waitlist – did not receive any intervention.	Depressive Symptoms: measured with Beck Depression Inventory Anxiety: measured with Beck Anxiety Inventory and Spielberger State‐Trait Anxiety Inventory Worry: measured with Penn State Worry Questionnaire
Thabrew (2022)	Randomized Controlled Trial	To evaluate the efficacy and acceptability of “Whitu: seven ways in seven days,” a well‐being application (app) for young people.	New Zealand residents aged between 16 and 30	*n* = 90 Mean age: 23.68 Female: 87.8% Male:10% Non‐Binary: 2.2% New‐Zealand European: 27.8% Maori: 43.3% Pacific: 12.2% Asian: 10% Other: 6.7%	Whitu: seven ways in 7 days is a free mobile application (app) that is currently available to New Zealand users. It contains seven positive psychology, CBT and psychoeducation‐based modules that can be completed within a week.	Control group: waitlist – did not receive any intervention.	Emotional well‐being was measured using the 5‐item WHO Well‐Being Index (WHO‐5) Mental well‐being was measured by the 7‐item Short Warwick‐Edinburgh Mental Well‐Being Scale. Depression was measured by the 20‐item Center for Epidemiological Studies Depres‐sion Scale (CES‐D). Anxiety was measured by the Generalized Anxiety Disorder 7‐item Scale.36 Self‐compassion was measured by the Self‐Compassion Scale‐Short Form. Stress was measured by the 10‐item Perceived Stress Scale (PSS‐10). Sleep quality was measured by the single‐item Sleep Quality Scale (SQS).
Thompson (2020)	Randomized Controlled Trial	(1) To determine the effect of the mobile app, called on OnTrack in decreasing alcohol and marijuana use and sexual risk behaviors.	Inner‐city crisis shelter for homeless young adults in Urban Northeastern USA	*n* = 60 Mean Age: 19.2 Gender: 25% female, 75% male Ethnicity: 66.7% Black, 10% Caucasian, 1.7% Hispanic, 23.3% Other Other Criteria: Homeless	On Track Application: the app allows for self‐monitoring of substance use and sexual risk behaviors, in addition to Brief Motivational Interviewing	Control group: treatment as usual, which included two components: (a) substance use treatment and referral and HIV testing (b) brief meetings with a research coordinator every 2 weeks, which involved completing measures for alcohol and marijuana use and risky sexual behaviors.	Number of Alcohol Drinks: measured by self‐report Use of Marjuana: measured by self‐report Unprotected Sex: measured by self‐report Alcohol or Drug Use before sex: measured by self‐report
Torok (2022)	2‐arm parallel, double‐blind, randomized controlled trial	To assess whether a therapeutic smartphone application (“LifeBuoy”) was superior to an attention‐matched control application at reducing the severity of suicidal ideation.	Australia	*n* = 455 Age: 21.5 Female: 84.6% Male/Other: 15.4% LGBTQI: 53.6%	7‐module, self‐guided CBT smart‐ phone application (“LifeBuoy”) designed to improve emotional regulation and increase distress tolerance skills.	Control group: sham app, LifeBuoy‐C, which was designed to match LifeBuoy on expectancy and time on task to control for digital placebo effects.	SIDAS is a validated, 5 item, 11‐point scale measure (Cronbach's *α* = 0.62), which assesses the frequency of ideation, controllability, severity, and impact. Recent depression symptoms (in past 2 weeks) were measured by the Patient Health Questionnaire‐9 (PHQ‐9; Anxiety symp‐toms were measured using the Generalized Anxiety Disorder‐7 (GAD‐7) Well‐being was measured using the 7‐item Short Warwick–Edinburgh Mental Well‐Being Scale Psychological distress was assessed using the Distress Questionnaire‐5 (DQ5)
Visser (2020)	Randomized Controlled Trial	(1) To assess the effect of a mobile app that uses memory bias modification on the emotional valence and strength of spontaneous autobiographical memories.	Radboud University in Nijmegen Social Sciences faculty, in the Netherlands	*n* = 153 Mean Age: 22.8 Gender: 76% female, 24% male Other Criteria: diagnosis of depression	Memory bias modification (MBM) Application: the app was designed to to habituate the process of autobiographical information retrieval with either a positive or negative emotional valence, depending on participant's group. Positive Group was prompted to think of the most pleasant or positive event since the previous prompt. How was the event experienced? Describe this event in three words Negative Group was prompted to think of the most unpleasant or negative event since the previous prompt. Describe this event in three words	Control group: Sham app required participants used the same MBM app but were prompted to think of neutral topics including study‐ or work‐related events. Sham Group: was prompted to think of an event related to work or study that occurred since the previous prompt. How was the event experience? Describe this event in three words	Depressive Symptoms: measured with The Beck Depression Inventory
Yang (2022)	Randomized Controlled Trial	To develop and examine the effectiveness of an app‐based CBT program, called HARU ASD, to alleviate anxiety among adolescents and young adults with ASD without ID	South Korea	*n* = 30 Mean Age: 20.97 Gender: 27/30 male, 3/30 female Other criteria: A documented medical record of ASD	The participants in the intervention group were given the HARU ASD3 program, an app‐based CBT program for reducing anxiety in persons with ASD	Waitlist control group	Anxiety: State‐Trait Anxiety Inventory (STAI) Dysfunctional automatic thoughts: Automatic Thought Questionnaire‐Negative (ATQ‐N) Emotional State: Positive and Negative Affect Schedule (PANAS) Aberrant behaviors: Aberrant Behavior Checklist (ABC) Anxiety‐related behaviors: Direct behavior observation

#### 6.1.3 Excluded studies

We excluded 393 studies after full‐text assessment. Among these, 230 citations were identified as study protocols or trial registrations. We excluded 40 studies because the population was not youth aged 15–24 and 27 studies due to wrong intervention. Twenty‐four conference abstracts were excluded, followed by 13 studies which were not RCTs and an additional 11 not reporting relevant mental health outcomes. Finally, we excluded 9 duplicates and 2 dissertations (Figure 2).

### 6.2 Risk of bias in included studies

**Figure 3:**
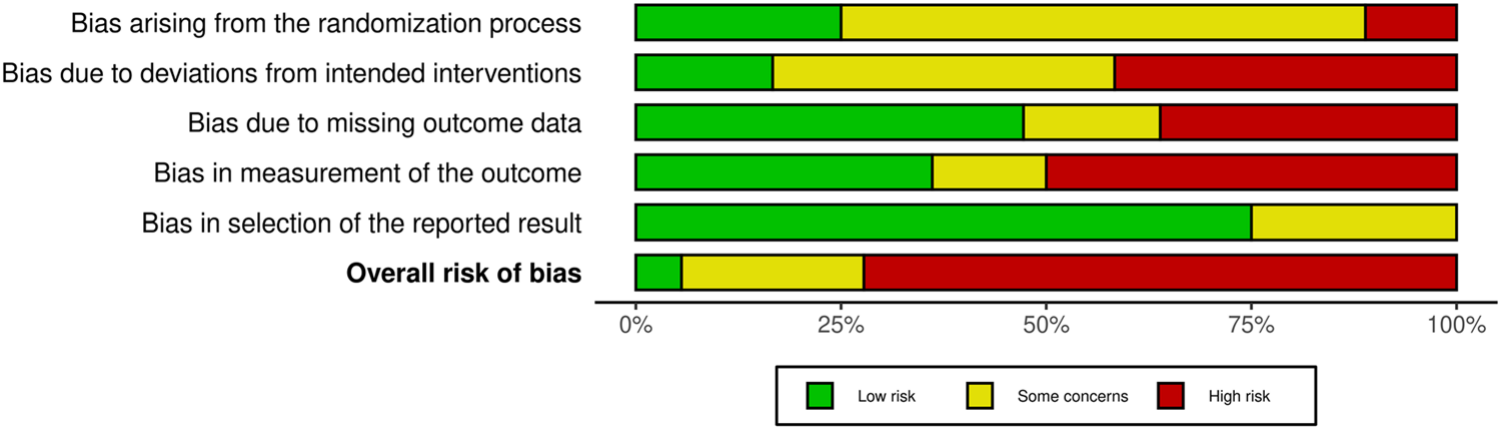
Risk of bias assessments using RoB 2.0.

**Figure 4:**
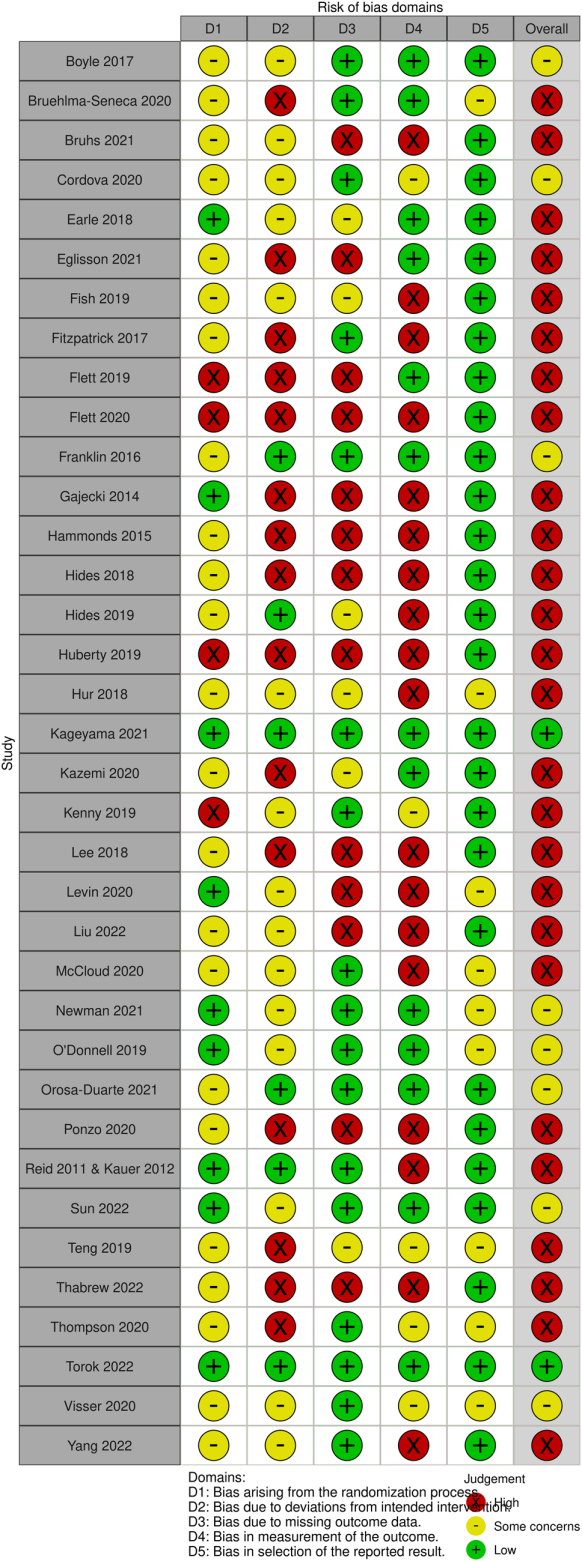
Risk of bias summary (RoB 2.0).

Among the 36 included trials, we assessed two with an overall low risk of bias, 8 trials with some concern regarding risk of bias, and 26 trials with a high risk of bias. The risk of bias assessments across 5 domains are summarized in Figure 3, and the individual study assessments are presented in Figure 4.

#### 6.2.1 Bias arising from the randomization process

Domain 1 examined risk of bias arising from the randomization process. Studies were classified as low risk if the allocation sequence was random, concealed to participants and that there were no baseline differences between groups to suggest a problem with the randomization process. Nine trials (25%) were rated as low risk of bias, 23 (64%) trials had some concerns, and the remaining 4 (11%) had high risk of bias. The most common issue across studies was a lack of information about allocation concealment.

#### 6.2.2 Bias due to deviations from intended interventions

Domain 2 evaluated risk of bias due to deviations from the intended interventions. This was assessed by examining participant's and assessor's blindness to the intervention and consequently if any deviations from the intended intervention and its potential effect on outcome. Domain 2 also evaluated the appropriateness the analysis used to estimate the effect of assignment to intervention. Six trials (16%) were rated as low risk of bias, 15 trials (42%) had some concerns, and 15 trials (42%) were rated as high risk of bias. In most studies, it was not possible to blind the participants nor the outcome assessor to the intervention.

#### 6.2.3 Bias due to missing outcome data

Domain 3 examined risk of bias due to missing outcome data. We evaluated whether studies reported that all outcome data was available for nearly all randomized participants, and if not what effect this could have on biassing the results and affecting the true values. Seventeen trials (47%) had a low risk of bias, 6 trials (17%) had some concerns, and 13 trials (36%) had a high risk of bias regarding missing outcome data. The primary concern in these studies was a high rate of attrition and/or unequal attrition across intervention and control groups.

#### 6.2.4 Bias in measurement of the outcome

Domain 4 evaluated risk of bias in measurement of outcomes. To assess Domain 4, studies were evaluated on the appropriateness of their method to measure outcomes and whether the outcome assessors were blinded. Further, there was ascertainment of whether measurement of the outcomes could have differed between groups, as well as if the outcomes could have been influenced by the assessor's knowledge of intervention received. We assessed 13 trials (36%) as low risk of bias, 5 trials (14%) with some concerns, and 18 trials (50%) with high risk of bias. Most often, outcome assessors and participants as self‐assessors were not blinded to condition assignment and their knowledge may have influenced the assessment of outcomes.

#### 6.2.5 Bias in selection of the reported result

Domain 5 focused on risk of bias in selection of the reported result. This was evaluated by whether a study's data was analyzed in accordance with a pre‐specified analysis plan and the likelihood that the numerical result being assessed was selected out of bias. Ratings for this domain were predominantly based on examination of a trial protocol or statistical analysis plan. Twenty‐seven trials (75%) had a low risk of bias and 9 trials (25%) had some concerns regarding risk of bias. No trials were assessed as having high risk of bias for this domain.

### 6.3 Effects of interventions

#### 6.3.1 Synthesis of results

##### Mean effect of mobile apps on depressive symptoms

A total of 20 studies (21 publications) reported the impact of mobile apps on the severity of depressive symptoms among youth (Bruehlman‐Senecal, 2020; Bruhns, 2021; Egilsson, 2021; Fish, 2019; Fitzpatrick, 2017; Flett, 2019; Hammonds, 2015; Hur, 2018; Kageyama, 2021; Kauer, 2012; Lee, 2018; Levin, 2020; Liu, 2022; McCloud, 2020; Ponzo, 2020; Reid, 2011; Sun, 2022; Teng, 2019; Thabrew, 2022; Torok, 2022; Visser, 2020).

###### Mobile apps compared to withheld controls (i.e., no intervention, waitlisting)

**Figure 5:**
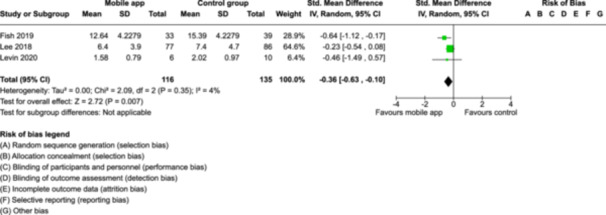
Analysis 1.1 for the outcome of depression; Mindfulness‐based mobile apps versus withheld controls.

Three studies provided youth with mindfulness‐based mobile apps and compared their impact to withheld controls (Fish, 2019; Lee, 2018; Levin, 2020). A meta‐analysis of the three studies (Analysis 1.1) found a statistically significant and potentially clinically important improvement in depressive symptoms among participants receiving mindfulness‐based apps in the short term (Pooled SMD = −0.36; 95% CI: −0.63, −0.10; *p* = 0.007) (Fish, 2019; Lee, 2018; Levin, 2020) (Figure 5). Pooled results were statistically homogeneous (*I* ^2^ = 4%; *χ* ^2^ *p* = 0.35; *τ* ^2^ = 0.00) and GRADE certainty of evidence was very low.

**Analysis 1.1:**
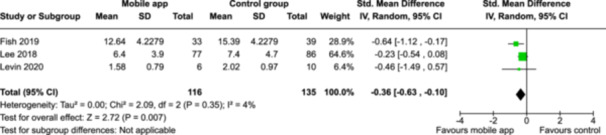
Comparison 1: Mean effect of mobile apps on depressive symptoms, Outcome 1: Depression: Mindfulness‐based mobile apps versus withheld controls.

**Figure 6:**
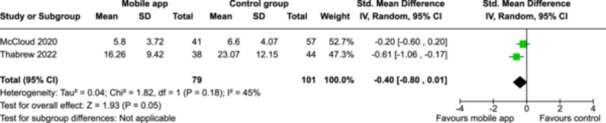
Analysis 1.2 for the outcome of depression; Cognitive Behavioral Therapy (CBT)‐based mobile apps versus withheld controls.

Two studies provided youth with CBT‐based mobile apps and compared their impact to withheld controls (McCloud, 2020; Thabrew, 2022). A meta‐analysis of the two studies (Analysis 1.2) found statistically significant and potentially clinically important improvements in depressive symptoms favouring the intervention group compared to the control group in the short term (Pooled SMD = −0.40; 95% CI: −0.80, 0.01; *p* = 0.05) (McCloud, 2020; Thabrew, 2022) (Figure 6). There was a moderate degree of statistical heterogeneity among pooled results (*I* ^2^ = 45%; *χ* ^2^ *p* = 0.18; *τ* ^2^ = 0.04) but the small number of studies in the meta‐analysis (*n* = 2) prevented conducting sensitivity analyses. GRADE certainty of evidence was very low.

**Analysis 1.2:**
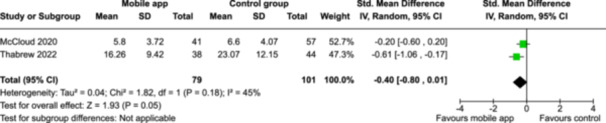
Comparison 1: Mean effect of mobile apps on depressive symptoms, Outcome 2: Depression: Cognitive Behavioral Therapy (CBT)‐based mobile apps versus withheld controls.

**Figure 7:**
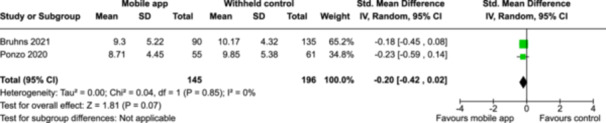
Analysis 1.3 for the outcome of depression; Combination therapy (CBT and mindfulness) mobile apps versus withheld controls.

Two additional studies used both CBT and mindfulness as framework for their intervention design (Bruhns, 2021; Ponzo, 2020). A meta‐analysis of the two studies (Analysis 1.3) showed improvements in depression symptoms that did not reach statistical significance among participants receiving the intervention versus those waitlisted to receive it in the short term (Pooled SMD = −0.20; 95% CI: −0.42, 0.02; *p* = 0.07) (Bruhns, 2021; Ponzo, 2020) (Figure 7). Pooled results were statistically homogeneous (*I* ^2^ = 0%; *χ* ^2^ *p* = 0.85; *τ* ^2^ = 0.00), with a very low GRADE certainty of evidence.

**Analysis 1.3:**
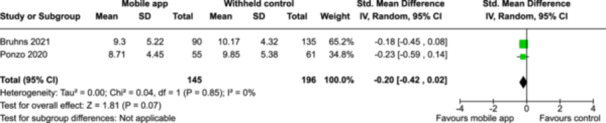
Comparison 1: Mean effect of mobile apps on depressive symptoms, Outcome 3: Depression: Combination therapy (CBT and mindfulness) mobile apps versus withheld controls.

Four additional studies compared the severity of depressive symptoms among participants receiving mobile apps to those receiving withheld controls (Bruehlman‐Senecal, 2020; Egilsson, 2021; Hammonds, 2015; Kageyama, 2021), but the clinical heterogeneity arising from the variability of mental health therapy designs prevented pooling their results. The mobile apps used different techniques to manage depressive symptoms, including antidepressant reminders (Hammonds, 2015), cognitive bias modification or positive stimulation (Kageyama, 2021), gamified health promotion (Egilsson, 2021), or social skill building to address loneliness (Bruehlman‐Senecal, 2020). The four studies reported no statistically significant differences in depressive symptoms at follow‐up between the intervention and the control groups (Bruehlman‐Senecal, 2020; Egilsson, 2021; Hammonds, 2015; Kageyama, 2021).

###### Mobile apps compared to active controls (i.e., controlled or sham mobile app)

**Figure 8:**
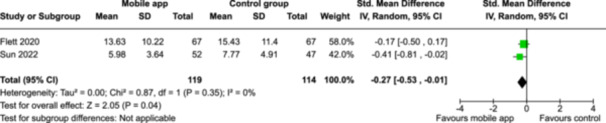
Analysis 1.4 for the outcome of depression; Mindfulness‐based mobile apps versus active controls.

Two studies compared mindfulness‐based apps to active controls (Flett, 2019; Sun, 2022). A meta‐analysis (Analysis 1.4) found a statistically significant and potentially clinically important improvement in depressive symptoms favouring the intervention group in the short term (Pooled SMD = −0.27; 95% CI: −0.53, −0.01; *p* = 0.04) (Flett, 2019; Sun, 2022) (Figure 8). Pooled results were statistically homogeneous (*I* ^2^ = 0%; *χ* ^2^ *p* = 0.35; *τ* ^2^ = 0.00) and GRADE certainty of evidence was very low.

**Analysis 1.4:**
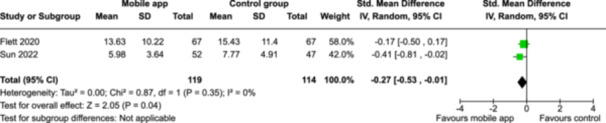
Comparison 1: Mean effect of mobile apps on depressive symptoms, Outcome 4: Depression: Mindfulness‐based mobile apps versus active controls.

**Figure 9:**
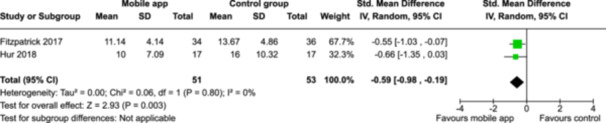
Analysis 1.5 for the outcome of depression; Cognitive Behavioral Therapy (CBT)‐based mobile apps versus active controls.

Three studies compared mobile apps utilizing CBT to active controls (Fitzpatrick, 2017; Hur, 2018; Liu, 2022). A meta‐analysis of two studies (Analysis 1.5) found statistically significant and potentially clinically important improvements in depressive symptoms among participants receiving CBT‐based mobile apps in the short term (Pooled SMD = −0.59; 95% CI: −0.98, −0.19; *p* = 0.003) (Fitzpatrick, 2017; Hur, 2018) (Figure 9). Pooled results were statistically homogeneous (*I* ^2^ = 0%; *χ* ^2^ *p* = 0.8; *τ* ^2^ = 0.00), with a very low GRADE certainty of evidence. An additional study compared depressive symptoms in the medium term (i.e., 4 months) and found that participants using a chatbot designed on the principles of CBT showed a statistically significant improvements in depression compared to those using bibliotherapy (Evidence not sufficient to synthesize: ANCOVA *F* = 22.89, *p* < 0.01) (Liu, 2022).

**Analysis 1.5:**
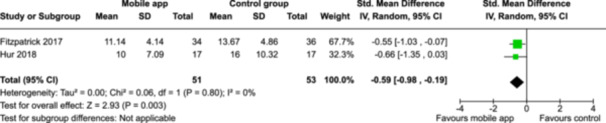
Comparison 1: Mean effect of mobile apps on depressive symptoms, Outcome 5: Depression: Cognitive Behavioral Therapy (CBT)‐based mobile apps versus active controls.

Four additional studies (Five publications) compared the severity of depressive symptoms among participants receiving mobile apps to those receiving active control (Kauer, 2012; Reid, 2011; Teng, 2019; Torok, 2022; Visser, 2020), but the clinical heterogeneity arising from the variability of mental health therapy designs prevented pooling their results. The mobile apps used different techniques to manage depressive symptoms, including DBT (Torok, 2022), self‐monitoring of mental health symptoms (Kauer, 2012; Reid, 2011), or cognitive bias modification or positive stimulation (Teng, 2019; Visser, 2020). The four studies reported no statistically significant differences at follow‐up between the intervention and the control groups (Kauer, 2012; Reid, 2011; Teng, 2019; Torok, 2022; Visser, 2020).

##### Mean effect of mobile apps on anxiety symptoms

A total of 19 studies reported the impact of mobile apps on the severity of anxiety symptoms among youth (Bruehlman‐Senecal, 2020; Egilsson, 2021; Fitzpatrick, 2017; Flett, 2019; Hur, 2018; Kageyama, 2021; Lee, 2018; Levin, 2020; Liu, 2022; McCloud, 2020; Newman, 2021; Orosa‐Duarte, 2021; Ponzo, 2020; Reid, 2011; Sun, 2022; Teng, 2019; Thabrew, 2022; Torok, 2022; Yang, 2022).

###### Mobile apps compared to withheld controls (i.e., no intervention, waitlisting)

**Figure 10:**
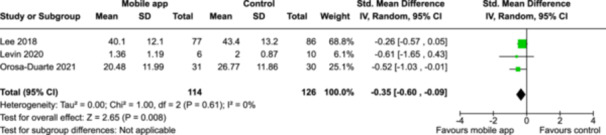
Analysis 2.1 for the outcome of anxiety; Mindfulness‐based mobile apps versus withheld controls.

Three studies provided youth with mindfulness‐based mobile apps and compared their impact to withheld controls (Lee, 2018; Levin, 2020; Orosa‐Duarte, 2021). A meta‐analysis of the three studies (Analysis 2.1) found statistically significant improvements in anxiety symptoms favouring participants in the intervention group compared to those in the control group in the short term (Pooled SMD = −0.35; 95% CI: −0.60, −0.09; *p* = 0.008) (Lee, 2018; Levin, 2020; Orosa‐Duarte, 2021) (Figure 10). Pooled results were statistically homogeneous (*I* ^2^ = 0%; *χ* ^2^ *p* = 0.61; *τ* ^2^ = 0.00) and GRADE certainty of evidence was very low.

**Analysis 2.1:**
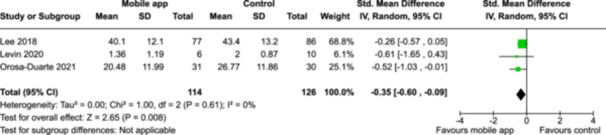
Comparison 2: Mean effect of mobile apps on anxiety symptoms, Outcome 1: Anxiety: Mindfulness‐based mobile apps versus withheld controls.

**Figure 11:**
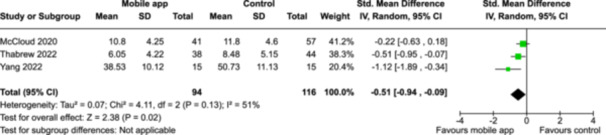
Analysis 2.2 for the outcome of anxiety; Cognitive Behavioral Therapy (CBT)‐based mobile apps versus withheld controls.

**Figure 12:**
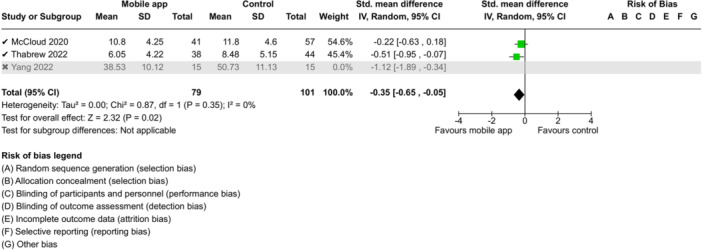
Sensitivity analysis of: 2.2 Anxiety: Cognitive Behavioral Therapy (CBT)‐based mobile apps versus withheld controls).

Three additional studies provided youth participants with CBT‐based mobile apps and compared their impact to withheld controls (McCloud, 2020; Thabrew, 2022; Yang, 2022). A meta‐analysis of the three studies (Analysis 2.2) found statistically significant improvements in anxiety symptoms among participants in the intervention group compared to those in the control group in the short term (Pooled SMD = −0.51; 95% CI: −0.94, −0.09; *p* = 0.02) (McCloud, 2020; Thabrew, 2022; Yang, 2022) (Figure 11). There was a moderate degree of statistical heterogeneity (*I* ^2^ = 51%; *χ* ^2^ *p* = 0.13; *τ* ^2^ = 0.07) and GRADE certainty of evidence was very low. As such we conducted a sensitivity analysis to explore the source of this heterogeneity, by visually examining the forest plot and removing data from the study with a confidence interval not overlapping with those of the remaining studies (Yang, 2022). Our sensitivity analysis corrected the statistical heterogeneity (*χ* ^2^ *p* = 0.35; *I* ^2^ = 0%; *τ* ^2^ = 0.00) and while the pooled result decreased, it remained statistically significant (Pooled SMD = −0.35; 95% CI: −0.65, −0.05; *p* = 0.002) (McCloud, 2020; Thabrew, 2022) (Figure 12). Certainty of evidence continued to be very low.

**Analysis 2.2:**
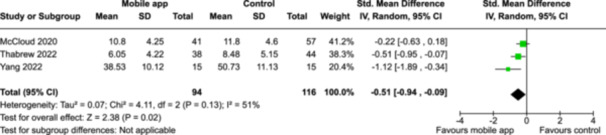
Comparison 2: Mean effect of mobile apps on anxiety symptoms, Outcome 2: Anxiety: Cognitive Behavioral Therapy (CBT)‐based mobile apps versus withheld controls.

**Figure 13:**
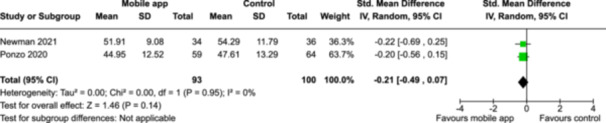
Analysis 2.3 for the outcome of anxiety; Combination therapy (CBT and mindfulness) mobile apps versus withheld controls.

Furthermore, two studies used both CBT and mindfulness as primary frameworks for designing their mobile apps and compared these interventions to withheld controls (Newman, 2021; Ponzo, 2020). A meta‐analysis of these two studies (Analysis 2.3) showed improvements in anxiety symptoms that approached statistical significance among participants in the intervention group compared to those in the control group in the short term (Pooled SMD = −0.21; 95% CI: −0.49, 0.07; *p* = 0.14) (Newman, 2021; Ponzo, 2020) (Figure 13). Results were statistically homogeneous (*I* ^2^ = 0%; *χ* ^2^ *p* = 0.95; *τ* ^2^ = 0.00) and GRADE certainty of evidence was very low.

**Analysis 2.3:**
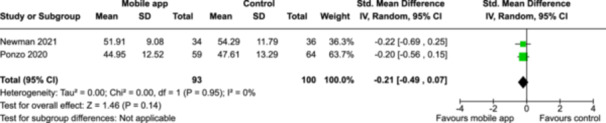
Comparison 2: Mean effect of mobile apps on anxiety symptoms, Outcome 3: Anxiety: Combination therapy (CBT and mindfulness) mobile apps versus withheld controls.

Three additional studies compared the severity of anxiety symptoms among participants receiving mobile apps to those receiving withheld controls (Bruehlman‐Senecal, 2020; Egilsson, 2021; Kageyama, 2021), but the clinical heterogeneity arising from the variability of mental health therapy designs prevented the pooling of results. The mobile apps used different techniques to manage anxiety symptoms, including cognitive bias modification or positive stimulation (Kageyama, 2021), gamified health promotion (Egilsson, 2021), or social skill building to address loneliness (Bruehlman‐Senecal, 2020). Results showed no statistically significant differences in anxiety symptoms at follow‐up between the intervention and the control groups (Bruehlman‐Senecal, 2020; Egilsson, 2021; Kageyama, 2021).

###### Mobile apps compared to active controls (i.e., controlled or sham mobile app)

**Figure 14:**
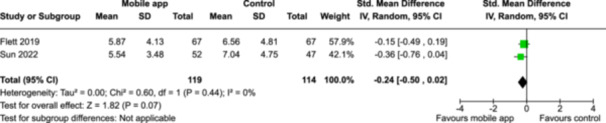
Analysis 2.4 for the outcome of anxiety; Mindfulness‐based mobile apps versus active controls.

Two studies compared mindfulness‐based apps to active controls (Flett, 2019; Sun, 2022). A meta‐analysis of the two studies (Analysis 2.4) found improvements in anxiety symptoms that approached statistical significance among participants in the intervention group compared to those in the control group in the short term (Pooled SMD = −0.24; 95% CI: −0.50, 0.02; *p* = 0.07) (Flett, 2019; Sun, 2022) (Figure 14). Pooled results were statistically homogeneous (*I* ^2^ = 0%; *χ* ^2^ *p* = 0.44; *τ* ^2^ = 0.00) and GRADE certainty of evidence was very low.

**Analysis 2.4:**
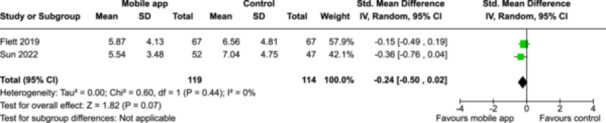
Comparison 2: Mean effect of mobile apps on anxiety symptoms, Outcome 4: Anxiety: Mindfulness‐based mobile apps versus active controls.

**Figure 15:**
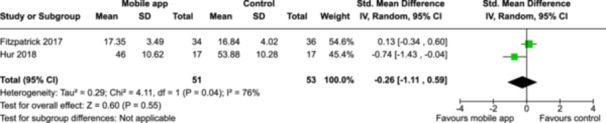
Analysis 2.5 for the outcome of anxiety; Cognitive Behavioral Therapy (CBT)‐based apps versus active controls.

Three additional studies provided youth participants with CBT‐based mobile apps and compared their impact to active controls (Fitzpatrick, 2017; Hur, 2018; Liu, 2022). A meta‐analysis of two studies (Analysis 2.5) found that improvements in anxiety symptoms did not reach statistical significance among participants in the intervention group compared to those in the control group in the short term (Pooled SMD = −0.26; 95% CI: −1.11, 0.59; *p* = 0.55) (Fitzpatrick, 2017; Hur, 2018) (Figure 15). Results were largely statistically heterogeneous (*I* ^2^ = 76%; *χ* ^2^ *p* = 0.04; *τ* ^2^ = 0.29), but the small number of included studies in the meta‐analysis (*n* = 2) prevented conducting a sensitivity analysis. GRADE certainty of evidence was very low. An additional study compared anxiety symptoms in the medium term (i.e., 4 months) and found that participants using a chatbot designed on the principles of CBT showed a statistically significant improvements in anxiety symptoms compared to those using bibliotherapy (Evidence not sufficient to synthesize: ANCOVA *F* = 5.37, *p* = 0.02) (Liu, 2022).

**Analysis 2.5:**
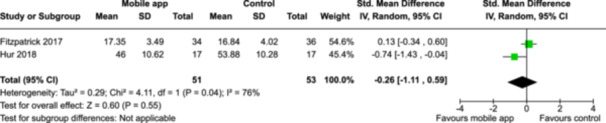
Comparison 2: Mean effect of mobile apps on anxiety symptoms, Outcome 5: Anxiety: Cognitive Behavioral Therapy (CBT)‐based mobile apps versus active controls.

Three additional studies compared the severity of anxiety symptoms among participants receiving mobile apps to those receiving active controls (Reid, 2011; Teng, 2019; Torok, 2022), but the clinical heterogeneity arising from the variability of mental health therapy designs prevented the pooling of results. The mobile apps used different techniques to manage anxiety symptoms, including self‐monitoring of mental health symptoms (Reid, 2011), cognitive bias modification or positive stimulation (Teng, 2019), or DBT (Torok, 2022). One study of an attention bias modification mobile app showed a statistically significant improvement in trait (Evidence not sufficient to synthesize: *F*_8, 316_ = 2.06; *p* < 0.05) but not state anxiety (*p* = 0.59) (Teng, 2019). The remaining interventions showed no statistically significant differences in anxiety symptoms at follow‐up between the intervention and the control groups (Reid, 2011; Torok, 2022).

##### Mean effect of mobile apps on psychological stress

A total of 13 studies reported the impact of mobile apps on psychological stress and wellbeing among youth (Flett, 2019, 2020; Hides, 2019; Huberty, 2019; Kageyama, 2021; Kenny, 2020; Lee, 2018; Levin, 2020; Newman, 2021; Ponzo, 2020; Reid, 2011; Thabrew, 2022; Torok, 2022).

###### Mobile apps compared to withheld controls (i.e., no intervention, waitlisting)

**Figure 16:**
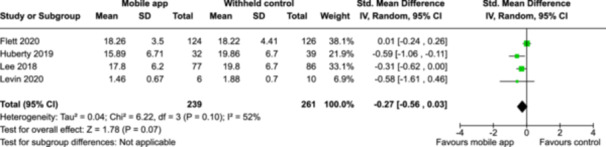
Analysis 3.1 for the outcome of psychological stress; Mindfulness‐based mobile apps versus withheld controls.

**Figure 17:**
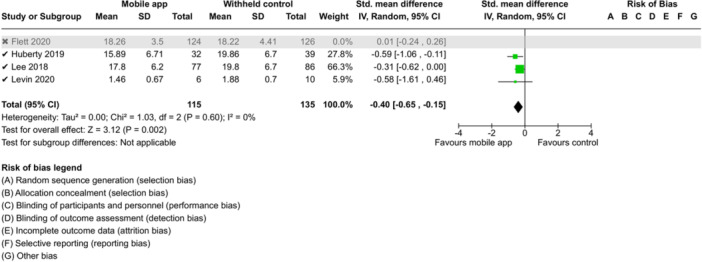
Sensitivity analysis of: 3.1 Psychological stress: Mindfulness‐based mobile apps versus withheld controls).

Four studies provided youth participants with mindfulness‐based mobile apps and compared their impact to withheld controls (Flett, 2019; Huberty, 2019; Lee, 2018; Levin, 2020). A meta‐analysis of the four studies (Analysis 3.1) showed improvements in psychological stress that approached statistical significance and clinical importance among participants receiving the mindfulness mobile apps compared to those in the withheld control (Pooled SMD = −0.27; 95% CI: −0.56, 0.03; *p* = 0.07) (Flett, 2020; Huberty, 2019; Lee, 2018; Levin, 2020) (Figure 16). Results were moderately statistically heterogeneous (*χ* ^2^ *p* = 0.1; *I* ^2^ = 52%; *τ* ^2^ = 0.04) and GRADE certainty of evidence was very low. As such we conducted a sensitivity analysis to explore the source of this heterogeneity, by visually examining the forest plot and removing data from the study with a confidence interval not overlapping with those of the remaining studies (Flett, 2020). Our sensitivity analysis corrected the statistical heterogeneity (*χ* ^2^ *p* = 0.6; *I* ^2^ = 0%; *τ* ^2^ = 0.00) and rendered the pooled result statistically significant (Pooled SMD = −0.40; 95% CI: −0.65, −0.15; *p* = 0.002) (Huberty, 2019; Lee, 2018; Levin, 2020) (Figure 17). Certainty of evidence continued to be very low.

**Analysis 3.1:**
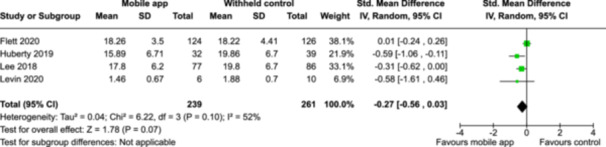
Comparison 3: Mean effect of mobile apps on psychological stress, Outcome 1: Psychological stress: Mindfulness‐based mobile apps versus withheld controls.

Only one study examined CBT‐based mobile apps and found statistically significant improvements in psychological stress compared to a withheld control (MD = −3.85; 95% CI: −6.77, 0.91; *p* = 0.01) (Thabrew, 2022). Furthermore, two studies used a combination of CBT and mindfulness elements in the therapy design of their mobile apps and compared them to withheld controls (Newman, 2021; Ponzo, 2020). While one study found a statistically significant increase in psychological wellbeing (MD = 5.57; regression coefficient B = −3.65; 95% CI: −6.1, −1.18; *p* = 0.004) (Ponzo, 2020), another failed to find a statistically significant decrease in psychological stress (Newman, 2021).

Three additional studies compared perceived psychological stress among participants receiving mobile apps to those receiving withheld controls (Hides, 2019; Kageyama, 2021; Kenny, 2020), but the clinical heterogeneity arising from the variability of mental health therapy designs prevented pooling their results. The mobile apps used different techniques to manage stress, including music therapy (Hides, 2019), self‐monitoring of mental health symptoms and emotions (Kenny, 2020), or positive stimulation (Kageyama, 2021). Studies showed no statistically significant differences in psychological stress at follow‐up between the intervention and the control groups (Hides, 2019; Kageyama, 2021; Kenny, 2020).

###### Mobile apps compared to active controls (i.e., controlled or sham mobile app)

One study compared a mindfulness‐based mobile app to an active control (Flett, 2019). There were no statistically significant improvements in psychological stress among participants in the intervention group compared to those in the control group (Flett, 2019).

Two additional studies compared perceived psychological stress among participants receiving mobile apps to those receiving active controls (Reid, 2011; Torok, 2022), but the clinical heterogeneity arising from the variability of mental health therapy designs prevented pooling their results. The mobile apps used different techniques to manage stress, including self‐monitoring of mental health symptoms and emotions (Reid, 2011), or DBT (Torok, 2022). Studies showed no statistically significant differences in psychological stress at follow‐up between the intervention and the control groups (Reid, 2011; Torok, 2022).

##### Mean effect of mobile apps on alcohol use

A total of 9 studies reported the impact of mobile apps on alcohol use among youth (Boyle, 2017; Cordova, 2020; Earle, 2018; Gajecki, 2014; Hides, 2018; Huberty, 2019; Kazemi, 2020; O'Donnel, 2019; Thompson, 2020), but clinical heterogeneity arising from mental health therapy design, outcome measurement, and time of follow‐up prevented pooling any of the results. The impact of mobile apps on reducing alcohol use is, thus, reported narratively.

Two studies provided youth participants with mobile apps that allowed them to self‐monitor blood alcohol levels or risky alcohol use behaviors before promoting behavioral changes (Gajecki, 2014; Thompson, 2020). One study measured alcohol drinking behaviors before engaging in sexual acts and found statistically significant lower odds of drinking alcohol among participants receiving the mobile app compared to those in the control group in the short term (OR = 0.14; 95% CI: 0.03, 0.64; *p* = 0.01) (Thompson, 2020). The other study measured the number of weekly binge drinking episodes and found no statistically significant differences between participants in the intervention group and those in the control group in the short term (Gajecki, 2014).

Two studies compared a gamified personalized normative feedback mobile app to an attention‐controlled app (Earle, 2018) or a web‐based version of the intervention (Boyle, 2017). In one study, and relative to an attention‐controlled app, pairwise comparisons showed a statistically significant reduction in the number of times that participants had “partied” during the past week in the short term (MD = −0.28; 95% CI: −0.47, −0.08; *p* = 0.005) (Earle, 2018). In the other study, and relative to the web‐based version of the intervention, regression results showed a statistically significant effect of intervention assignment favouring the mobile app group on alcohol consumption in the short term (Regression coefficient B = −0.1; SE = 0.03; *p* < 0.001) (Boyle, 2017).

Two studies of mobile apps that used brief motivational interviewing (IM) as the framework for mental health therapy design compared their impact on problematic alcohol use to withheld controls using the Alcohol Use Disorders Identification Test (AUDIT) (Hides, 2018; Kazemi, 2020). However, clinical heterogeneity arising from time of follow‐up prevented pooling results. One study measured the outcome in the short term (6 weeks follow‐up) and found reduction in AUDIT scores among participants in the intervention group that was statistically significant (*p* = 0.01), whereas changes among participants in the control group was not (Kazemi, 2020). The other study measured the outcome in the medium term (6 months follow‐up) and found that any reduction in AUDIT scores were not statistically significant (*F*_1, 182_ = 1.16; time × group interaction *p* = 0.28) (Hides, 2018).

Three additional studies delivered mobile apps that used different therapy designs to manage alcohol use, such as empowerment‐based interactive content (Cordova, 2020), ecological momentary intervention (EMI) (O'Donnel, 2019), and mindfulness (Huberty, 2019). One study measured past 30‐day alcohol use and found improvement favouring the intervention group compared to the control group that was not statistically significant in the short term (*p* = 0.14) (Cordova, 2020). Two studies, using two different therapy designs, measured risk single‐point alcohol use (i.e., binge drinking) and did not find any statistically significant improvements relating to this outcome in the short term (Huberty, 2019; O'Donnel, 2019).

## 7 DISCUSSION

### 7.1 Summary of main results

This systematic review synthesizes the effects of 36 randomized controlled trials conducted among youth from 15 countries. We investigated the effectiveness of mobile apps on the management of depressive symptoms, anxiety symptoms, psychological distress, and alcohol use. Our results demonstrate that apps with different therapy designs may improve mental health outcomes, but the evidence base is very uncertain. Meta‐analyses showed that apps which used mindfulness or CBT designs reduced symptoms of anxiety and depression. Apps which combined CBT and mindfulness reduced psychological distress. The evidence on the effects on alcohol use were variable and inconsistent. Additional mental health therapy designs which considered empowerment, gamification and motivational strategies showed some promise to reduce alcohol use. Across all trials, only a small number reported effects of clinical importance, with reductions in symptoms reaching a minimal clinically important threshold (Kounali, 2020; Button, 2015; Kroenke, 2001). Despite limited clinical importance, no harms or adverse events associated with app use were identified.

A minimal clinical threshold should be viewed as an *estimate* of clinical importance, but it does allow us to better compare our results to in‐person or virtual mindfulness and CBT interventions. Without a doubt, traditional talk therapy, mindfulness programs (Maynard, 2017) and CBT therapy (Hofmann, 2012) show more significant effects on clinical outcomes. This contextualization suggests mobile apps have only slight clinical relevance at this time, and may be best viewed as adjunctive therapy while waiting for less accessible, but more effective, talk therapies. Therefore, mobile apps could represent the first step in a stepped‐care approach to addressing youth mental health.

A major contribution of this review is the analysis of effectiveness outcomes based on their underlying psychological theory and treatment approach (which we term “mental health therapy design”). This approach to meta‐analysis is unique in that it contributes a scientific foundation that allows a comparison of mobile apps with other evidence based psychological interventions. Specifically, our analytic approach allows scientists and clinicians to compare delivery mechanisms of CBT and mindfulness. For example, the findings of this review could be compared to in‐person CBT or other mindfulness training, or to apps which include coaching or linkage to therapy (Graham, 2020).

### 7.2 Overall completeness and applicability of evidence

Our systematic review represents an assessment of apps to manage depression, anxiety and alcohol use among youth aged 15–24. However, several limitations of the evidence base limit the applicability of our findings. First, we only included evidence from randomized trials, and it is therefore possible that other evidence pertaining to the effectiveness of mental health apps for youth may have been excluded. Additionally, we only searched for evidence in bibliographic databases and did not search the gray literature. Future reviews should consider additional sources of evidence to provide a more comprehensive assessment of the available evidence. Second, most trials were conducted among small samples of post‐secondary school students in high income countries. These cohorts typically represent well‐educated and high socioeconomic status populations. Future research should consider out‐of‐school youth and populations residing in low‐ and middle‐income countries. Third, our interventions of interest were apps designed to help manage existing symptoms of depression and anxiety, but not prevention. As such, we excluded apps which aimed to prevent mental health or otherwise focused on overall wellness. Given youths' avoidance of mental health interventions, this possibly represents a body of interventions that youth may access to indirectly help with mental health concerns. Finally, all studies were short‐term and we therefore have no indication of the long‐term potential of these apps to improve mental health. Future trials should be conducted with larger and more diverse populations of youth and assess effects at 6 months, 12 months, and beyond.

### 7.3 Quality of the evidence

The results of this review should be interpreted with caution. Across all outcomes and for all different intervention designs, we assessed a very low certainty of evidence. Future research is likely to change both the size and direction of the estimate of effect for depression, anxiety, psychological distress, and alcohol outcomes. Additionally, estimates are based on meta‐analyses using very few studies (*n* = 2–4 per analysis). These studies had small sample sizes which impacted the precision and power of the estimates. Further, most trials included in this review (26/36) had a high risk of bias, and only two trials were determined to be of low risk using the Cochrane Risk of Bias 2.0 tool. Collectively, these issues reduce the certainty in the review findings.

### 7.4 Potential biases in the review process

We conducted a search in 5 bibliographic databases relevant to the health sciences up to July 1, 2022. All citations were screened in teams by two independent screeners from the review team, and one review author (AS) assessed all included studies against inclusion criteria. However, we did not conduct a gray literature search. Therefore, it is possible that additional evidence exists that was not identified for inclusion in our review. We were unable to comment on the possibility of publication bias as very few studies were included in each meta‐analysis. Thus, we cannot rule out that there may be some studies reporting negative results that were not published or made public. Where we had sufficient data, we investigated statistically significant heterogeneity.

### 7.5 Agreements and disagreements with other studies or reviews

This systematic review complements and expands the evidence base in this area. Existing systematic reviews and meta analyses on the effectiveness of apps for students and youth have reported small statistically significant reductions in depressive and anxiety symptoms (Leech). Recent systematic reviews and meta‐analyses on apps for the general population also suggest small reductions (Zielasek, 2022; Wang, 2018; Miralles, 2020; Weisel, 2019; Wu, 2021). One overview of reviews on mental health apps for depressive and anxiety symptoms reports on 7 meta analyses (Lecomte, 2020). This review reports overall poor quality studies and suggests that many reviews have pooled heterogenous data.

Overall our findings are consistent with past reviews, but bring together more trials and provide more precision in our PICO interpretation. In summary, our review is able to report potentially important reductions in common mental illness symptoms. These findings generally align with the Lecomte review that suggest standalone apps are promising as standalone interventions or adjunctive treatments, but our review is unique in interpreting results as providing small clinically important differences.

Our unique ability to pool and analyze studies based on therapy design allows us to more easily compare the magnitude and certainly of our app delivery findings. In person delivered mindfulness interventions for adults (de Vibe, 2017), and group interventions for youth (Maynard, 2017) showed efficacy with small effect sizes. Individual and group CBT and self‐guided CBT intervention meta analyses also mirrored our findings, but with larger effect sizes and we note that self guided CBT approaches were considered less acceptable for patients (Cuijpers, 2019).

Our design analytic approach demonstrated marginal improvements from previous reviews. Many studies and reviews have also shown barriers to standalone apps (Borghouts, 2021) and the limited participant diversity in our studies suggest these barriers have yet to be breached in trials. One recent review highlights the importance of engagement (Zielasek, 2022) and we agree that this continues to be a key concern for clinical important outcomes.

## 8 AUTHORS' CONCLUSIONS

### 8.1 Implications for practice

Evidence from the literature shows that mobile apps offer a potentially scalable intervention that may be accessible and acceptable to young people (Montagni, 2020). Vulnerable groups, such as youth, will need tools that are effective, robust, and trustworthy and that improve long‐term mental health (The Lancet Digital Health). Future research should shed light on whether mobile apps may also play a supportive role in mental health psychoeducation for youth, and whether such apps can offer an adjunctive role to in‐person mental health care, thus redefining stepped mental health care and alleviating workload on mental health care professionals. Indeed some designs include early psychological therapy, but can also provide blended care and link to supportive coaching programs (Canadian Mental Health Association) and to in‐person clinical support. Digital therapeutics may therefore be redefining stepped mental health care (Cornish, 2017). CBT and mindfulness apps may reduce depressive and anxiety symptoms better than no intervention at all. However, small effects, high attrition rates and uncertain effects in educationally and culturally diverse populations continue to raise questions for large scale implementation. There is an urgent need for more evidence on benefits and harms in relation to existing mental health education, community and clinical practice.

Digital therapeutics have large potential policy implications. Scalability alone is worth highlighting, however to truly understand long‐term effectiveness, real‐word longitudinal data is needed. It is important to note, however, it is unclear whether standalone apps, even those using evidence based psychological designs, could have the same clinical impact as therapist‐guided interventions and whether these apps comply with existing privacy health legislation or health insurance coverage policies. It is also important to recognize the many potential barriers to uptake such as poor access to technology, disease severity, and education, cultural and linguistic diversity. Several potential evaluation challenges remain to be addressed, such as the evolving nature of app design, long‐term consistent outcome reporting and qualitative user experience data.

### 8.2 Implications for research

Mobile app interventions are emerging as accessible and usable resources that are showing some small statistically significant mental health differences for youth. The clinical importance of the digital therapeutics remains uncertain at this time, and this highlights the need for more research on specific psychological designs, intensity of interventions, duration of interventions, long‐term outcomes, adverse effects including opportunity costs and accessibility in the context of cultural diversity. In addition, apps may offer effective linkage to scare and this approach to stepped or blended mental health care deserves more research.

## DATA AND ANALYSES

Comparison 1

Mean effect of mobile apps on depressive symptoms

**Table:** 

Outcome or subgroup title	No. of studies	No. of participants	Statistical method	Effect size
1.1 Depression: Mindfulness‐based mobile apps versus withheld controls	3	251	Std. Mean Difference (IV, Random, 95% CI)	−0.36 [−0.63, −0.10]
1.2 Depression: Cognitive Behavioral Therapy (CBT)‐based mobile apps versus withheld controls	2	180	Std. Mean Difference (IV, Random, 95% CI)	−0.40 [−0.80, 0.01]
1.3 Depression: Combination therapy (CBT and mindfulness) mobile apps versus withheld controls	2	341	Std. Mean Difference (IV, Random, 95% CI)	−0.20 [−0.42, 0.02]
1.4 Depression: Mindfulness‐based mobile apps versus active controls	2	233	Std. Mean Difference (IV, Random, 95% CI)	−0.27 [−0.53, −0.01]
1.5 Depression: Cognitive Behavioral Therapy (CBT)‐based mobile apps versus active controls	2	104	Std. Mean Difference (IV, Random, 95% CI)	−0.59 [−0.98, −0.19]

Comparison 2

Mean effect of mobile apps on anxiety symptoms

**Table:** 

Outcome or subgroup title	No. of studies	No. of participants	Statistical method	Effect size
2.1 Anxiety: Mindfulness‐based mobile apps versus withheld controls	3	240	Std. Mean Difference (IV, Random, 95% CI)	−0.35 [−0.60, −0.09]
2.2 Anxiety: Cognitive Behavioral Therapy (CBT)‐based mobile apps versus withheld controls	3	210	Std. Mean Difference (IV, Random, 95% CI)	−0.51 [−0.94, −0.09]
2.3 Anxiety: Combination therapy (CBT and mindfulness) mobile apps versus withheld controls	2	193	Std. Mean Difference (IV, Random, 95% CI)	−0.21 [−0.49, 0.07]
2.4 Anxiety: Mindfulness‐based mobile apps versus active controls	2	233	Std. Mean Difference (IV, Random, 95% CI)	−0.24 [−0.50, 0.02]
2.5 Anxiety: Cognitive Behavioral Therapy (CBT)‐based mobile apps versus active controls	2	104	Std. Mean Difference (IV, Random, 95% CI)	−0.26 [−1.11, 0.59]

Comparison 3

Mean effect of mobile apps on psychological stress

**Table:** 

Outcome or subgroup title	No. of studies	No. of participants	Statistical method	Effect size
3.1 Psychological stress: Mindfulness‐based mobile apps versus withheld controls	4	500	Std. Mean Difference (IV, Random, 95% CI)	−0.27 [−0.56, 0.03]

## CONTRIBUTIONS OF AUTHORS

Content: Kevin Pottie, Dominique Ranger, Rinila Haridas, Olivia Magwood

Systematic review methods: Kevin Pottie, Olivia Magwood, Ammar Saad

Statistical analysis: Ammar Saad, Shahab Sayfi

Information retrieval: Olivia Magwood, Kate Volpini, Franklin Rukikamirera, Yvonne Tan, Jeremie Alexander, Ammar Saad, Shahab Sayfi

## DECLARATIONS OF INTEREST

The authors declare no potential conflicts of interest.

### Preliminary timeframe

Approximate date for submission of the systematic review: December 10, 2023.

### Plans for updating this review

Kevin Pottie will be responsible for updating this review.

## SOURCES OF SUPPORT

### Internal sources

Not applicable, Other

Not applicable

### External sources

College of Family Physicians of Canada, Canada

2019 Daniel Glazier Award in Adolescent Mental Health and Substance Abuse

## DIFFERENCES BETWEEN PROTOCOL AND REVIEW

We acknowledge the following differences between our published protocol (Magwood, 2022) and the current review:

1. Our protocol did not specify intervention design categories. This categorization was selected after reviewing the various interventions which met our eligibility criteria. This decision was made through discussion with our stakeholders.
2. Our protocol specified primary and secondary outcomes of interest. We have removed this hierarchy and treat all outcomes equivalently.
3. We did not meet our goal kappa statistic for inter‐rater agreement at the title/abstract screening stage. We introduced two measures to address this: We held screening training sessions for all reviewers and held weekly discussions to resolve discrepancies.
4. We did not prioritize RRs over ORs in our analyses, and present these values as reported in the source study.
5. There was insufficient data to support subgroup analyses by gender/sex, race and socio‐economic status.

## Supporting information

Supporting information.

Supporting information.
