# Supplementary material for: Mobile apps to reduce depressive symptoms and alcohol use in youth: A systematic review and meta‐analysis: A systematic review
Source: Campbell Syst Rev. 2024 Apr 26;20(2):e1398. doi: 10.1002/cl2.1398 (PMC11047135; doi:10.1002/cl2.1398)
Supplement: Supplementary file 1 — Supporting information. [file CL2-20-e1398-s002.docx]

Characteristics of studies

Characteristics of included studies [ordered by study ID]

[Boyle 2017](#STD-Boyle-2017)

| ***Study characteristics*** | |
| --- | --- |
| Methods | Design: RCT  Objective: to evaluate the ability of alcohol PNF delivered by a pilot version of CampusGANDR,which includes points, chance, and simulates the generation of norms by visible, Facebook-connected peers, to correct drinking norms and reduce alcohol use relative to PNF delivered by the standard online survey format currently used on college campuses  Data collection: Recruitment materials invited potential participants to be “beta testers” for a new Facebook application, called CampusGANDR.  Baseline, 2-week follow up |
| Participants | Setting: Loyola Marymount University, west coast, US, psychology department  Eligibility Criteria: To be eligible for participation, students had to be between 18 and 24 years old, have a valid Facebook account, and be available during two specific 72 h periods to complete sessions 1 and 2 of the study on a computer connected to the internet.  Characteristics: the majority (75.5%) of the sample were females, and 51.1% were in their freshman year. 48% of the sample were white (Caucasian), whereas 18.5% were Asians, and 16% were of hispanic/latino backgrounds. |
| Interventions | CampusGANDR Application: The app delivers the same components of PNF, but in a more engaging way. Session topics focused on alcohol, social media, and TV. The app is informed by both gamification and alcohol intervention research. |
| Outcomes | Perceived Descriptive Drinking Norms and Drinking Behavior: self report of the maximum drinks consumed on a single night so far during the semester, number of times participants had partied during the past week and number of drinks consumed during the previous weekend |
| Notes | Funding: Manuscript preparation was supported in part by grants R21AA021870-01 and R21AA022942-02 from the National Institute on Alcohol Abuse and Alcoholism.  Conflict of interest: All authors declare that they have no conflicts of interest |

[Bruehlman-Senecal 2020](#STD-Bruehlman_x002d_Senecal-2020)

| ***Study characteristics*** | |
| --- | --- |
| Methods | Design: Randomized Controlled Trial  Objective: To examine the initial efficacy, feasibility, and desirability of a smartphone app, Nod, designed to deliver cognitive and behavioral skill-building exercises to reduce loneliness during the transition to college  Data collection:(including timepoints for analysis)  Participants were recruited from July to September of 2019, in collaboration with the university’s first-year orientation program. All incoming students indicated whether they would like to receive information about a study examining the college transition via a question embedded within a longer orientation survey. Interested participants (N=2226) were sent additional information, and linked to a brief online screening survey containing questions to assess eligibility as well as an 8-item version of the UCLA loneliness questionnaire (UCLA-8)  Baseline, week 2, week 4, week 8 |
| Participants | Setting: in the northwestern United States  Eligibility Criteria: (a) entering their first year of undergraduate education, (b) aged 18 to 25 years, (c) English-literate, and (d) not residing with parents/guardians. Students also needed to have a smartphone with an operating system capable of supporting Nod (ie, Mac iOS 9-12 or Android OS 8-10), which 97.9% (806/823) of students who met the four eligibility criteria had.  Characteristics:  Total sample (n= 221)  For total:  Mean Age: 18.68  Gender: 59.3% female, 36.7% male  Ethnicity: 52.9% White, 13.6% Latino, 9.5% Asian, 3.6% Black, 0.9% Native American, 0.9% Hawaiian or Pacific Islander, 18.6% two or more races/ethnicities). |
| Interventions | Nod Application: a. Mobile app that addresses loneliness in first year college students by using positive psychology, mindfulness-based self-compassion, and cognitive behavioral skill-building exercises. |
| Outcomes | Loneliness: 8-item version of the UCLA loneliness 182 questionnaire  Anxiety and Depression symptoms: measured with 7-item Generalized Anxiety Disorder Scale and the Patient Health Questionnaire  Social Anxiety Symptoms: measured with 3-item Mini Social Phobia Inventory  Sleep Quality: measured with Pittsburgh sleep quality index  Perceived Social Support: measured with 3-item support subscale of the Comprehensive Inventory for Thriving |
| Notes | Funding: N/A Conflict of interest: Nod is a mobile app co-developed by Grit Digital Health and Hopelab. While both organizations own rights to the Nod product, Grit Digital Health is solely responsible for the commercial operation and distribution of Nod. As co-owner, Hopelab receives a limited portion of Nod sale net proceeds. The research reported here as well as the development of Nod were supported by the nonprofit Hopelab Foundation. Hopelab develops behavioral interventions to improve the health and well-being of young people. The design, conduct, analysis, and reporting of this study represent a scientific collaboration between Hopelab, JP, and BD at the University of Oregon, and KD at the University of California, San Francisco and Weill Institute for Neurosciences. EB-S, CJH, CF, JH, and DER are employed by Hopelab Foundation. The study sponsor was involved in the study design, collection, analysis, and interpretation of data; writing of the article; and decision to submit it for publication. |

[Bruhns 2021](#STD-Bruhns-2021)

| ***Study characteristics*** | |
| --- | --- |
| Methods |  |
| Participants |  |
| Interventions | "MCT & More": a smartphone app, primarily intended for individuals with depressive symptoms. The basic package comprises 57 short exercises based on group metacognitive training (MCT). Topics include: cognitive strategies, communication and interaction, positive activities, mindfulness and imagination, gambling, and meta-cognitive training.  In addition, the app contains gamification elements. Depending on the number of exercises completed, users can collect bronze, silver, or gold medals and obtain an open umbrella as a symbol for long-term protection. |
| Outcomes | Depression Module: (PHQ-9)  9-items on a 4-point rating scale. Total score between 0 and 27..  Self-esteem: (RSE) Rated on a 4-point rating scale, total score ranges from 10 to 40 points.  Quality of Life: (WHOQOL-BREF)  26 items (4 domains) Rated on a 5-point scale. Only the first question was used.  Online Intervention Attitude: (APOI) Consists of 4 dimensions, 16 items and can be rated on a 5-point rating scale. Total ranges from 16 to 80.  Therapy Expectation: (PATHEV) 10 Items, cover 3 subscale, 5-point rating scale, total scale ranges from 11 to 55.  Patient Satisfaction: (ZUF-8)  8 items, rated on a 4-point scale, a total score of 8-32..  Negative Effects of Psychotherapy: (INEP)  21 items, consists of 2 scales. on a 7-point scale, items 16-21 were excluded from the assessment. |
| Notes |  |

[Cordova 2020](#STD-Cordova-2020)

| ***Study characteristics*** | |
| --- | --- |
| Methods | Design: small scale pilot RCT  Objective: to determine the feasibility of S4E, relative to enhanced usual practice and evaluate changes in the potential mechanisms of change, namely clinician-youth risk communication, prevention knowledge, and self-efficacy over time  Data collection: Youth recruitment occurred during the clinic’s health appointment reminder phone calls.  Clinician recruitment occurred during weekly clinic staff meetings  Both youths and clinicians who expressed interest in participating in the study were contacted by the study team to screen for eligibility, to enroll, and to complete study consent protocols  At baseline, immediately postintervention, and 30 days postbaseline |
| Participants | Setting: youth centered community health clinic located in Southeast Michigan, US  Eligibility Criteria: To be eligible for this study, youth had to (1) be aged between 13 and 21 years, (2) live in Southeast Michigan without plans to move out of the area during the study period, (3) have a scheduled appointment with a participating clinician, and (4) report no prior history of psychiatric hospitalization  all clinicians at the health clinic were eligible to participate if they (1) worked in Southeast Michigan and (2) worked with our target population  Characteristics: Of the 50 youths: 41 (82%) female, 4 (8%) males, and 4 (8%) transmales, and 1 refused to respond. The mean age of the youths was 18.82 years (SD 2.1, range 13-21). 23 (46%) non-Hispanic white, 21 (42%) black, 1 (2%) Native American, 4 (8%) ascribing to more than one race, and 1 (2%) selecting Other. 36% (18/50) of youths reported having completed some college, 30% (15/50) reported having completed high school. 34% (17/50) of youths reported having completed a grade between 7th and 11th. |
| Interventions | S4E Application: Youth would receive targeted, tailored prevention content based on their responses to the S4E risk behavior assessment. This assessment is intended to identify the youths’ specific risk behaviors based on the past year and lifetime reports of substance use, sexual risk behaviors, and past 6-month STI/HIV testing practices. |
| Outcomes | Substance Use Refusal Skills: assessed through 2 separate items on a 4-point scale  Substance Use Prevention Knowledge: assessed through 2 separate items. Responses were on a 4-point agreement scale  Substance Use Behaviors: assessed using items adapted from the Monitoring the Future study (dichotomous yes/no) |
| Notes | Funding: Supported by pilot funding from the University of Michigan Rogel Cancer Center to David Cordova. Preparation of this manuscript was supported, in part, by grants from the National Institute of Mental Health to Torsten B Neilands (R25 MH067127), and the National Institute on Drug Abuse (R03DA04189101A1) to David Cordova.  Conflict of interest: None declared |

[Earle 2018](#STD-Earle-2018)

| ***Study characteristics*** | |
| --- | --- |
| Methods | Design: RCT  Objective: Aim 1a. Investigate the degree to which un-incentivized first-year students will self-select into the 6-week disguised intervention after minimal campus advertising  Aim 1b. Determine whether the proportion of alcohol and other drug related questions submitted by students during the game is high enough for this intervention approach to be feasible in the real world  Aim 2a. Measure the impact of PNF on descriptive same-sex drinking norms in this gamified format on students' perceived drinking norms and alcohol use 2 months later, relative to PNF on control topics only  Aim 2b. Examine whether supplementing this descriptive PNF with reflective opposite-sex feedback on participants' ownbehavior will promote greater reductions in alcohol use 2 months later  Aim 3. Test students' baseline drinking behavior as a moderator of the conditional effects of the intervention  Data collection:  Recruitment remained open for one week, during which 356 students visited the app’s website, consented to participate in the study, created a profile, and played the first round  Initial assessment (week 2, week 3, week 4) and 2 month follow-up |
| Participants | Setting: Loyola Marymount University, west coast, US, psychology department  Eligibility Criteria: (1) First year university student (2) 18yrs and older 3) with problematic alcohol consumption behaviours  Characteristics: The majority of participants were female (55%) and Caucasian (47%). Twenty percent of participants described themselves as Hispanic/Latino, 14% were Asian, 12% were African American, and 7% were Multiracial or Other. All participants were students. |
| Interventions | CampusGANDR (v1) Application: a gamified app that tests students’ perceptions of various college life topics with a system of points. Each question contained two parts. First, participants estimated how the average same-sex student in their class would answer. Next, students reported their own answer. Participants would later have access to a feedback module, that would allow participants’ misperceptions to be corrected and participants were shown how their behavior compared to that of their peers. |
| Outcomes | Self-Report questions on: (1) the maximum drinks consumed on a single night so far during the semester (2) number of times participants had partied during the past week (3) number of drinks consumed during the previous weekend. |
| Notes | Funding: Support for this research was provided by Grants R21AA024853 and R21AA022942 from the National Institute on Alcohol Abuse and Alcoholism. NIAAA had no role in the study design, collection, analysis or interpretation of the data, writing the manuscript, or the decision to submit the paper for publication.  Conflict of interest: None declared.  Other: Mainly a feasibility study with a small sample size |

[Egilsson 2021](#STD-Egilsson-2021)

| ***Study characteristics*** | |
| --- | --- |
| Methods | Design: A single-center randomized controlled public school pilot trial  Objective: The purpose of this study was to assess time-specific attrition rates in an adolescent mHealth intervention, as well as to describe usage and the intervention’s feasibility in relation to self-efficacy levels and participants’ emotional and physical health.  Data collection: Research specifications and mobile app introduction were sent to parents and legal guardians of all eligible participants through school officials by email, including a confirmative survey link along with parental information about possible exclusion criteria. Participation in the online survey was regarded as consent. The study was approved by the National Bioethics Committee |
| Participants | Setting: Elementary schools in Reykjavík, Iceland  Eligibility Criteria: All children born in 2001 attending a participating public school in Iceland’s capital area were eligible participants.  Characteristics: Participants were 41 individuals, including 17 girls and 24 boys, between 15 and 16 years of age attending a public school in the greater capital area of Iceland. The average age at baseline was 15.6 years (SD 0.26). All participants were native Icelandic speakers and owned smartphones at baseline; 56% (n=23) of participants had smartphones operating on iOS and 44% (n=18) had those operating on Android devices.  Among all invited participants in the intervention group, 70% (male:female ratio 8:6) began the intervention. Participants’ descriptive characteristics at baseline are summarized in Table 1. Retention after 6 weeks of the intervention was 65% among those who began the intervention (male:female ratio 5:4) |
| Interventions | SidekickHealth Application: an app centered on helping the user set goals and create health-related missions (gamification of tasks in three main categories: food and drink, physical activity, and mental health. |
| Outcomes | Self-efficacy: measured with 10-item General Self Efficacy Scale  BMI: measured with BMI index reference values for Swedish children adjusted for age and sex  Depressive symptoms: measured with Children’s Depression Inventory  Anxiety Symptoms: measured with Multidimensional Anxiety Scale  Sleep Problems: measured wtih BEARS sleep  screening algorithm  The amount, frequency, and time of daily physical activity:  measured through in-app activity  Self-Reported Stress Levels |
| Notes | Funding: The study was partially funded by a research grant from the Icelandic Research Fund (IRF 141381051).  Conflict of interest: EE is a minority shareholder and former employee of SidekickHealth AB. The other authors have no conflicts of interest to declare. |

[Fish 2019](#STD-Fish-2019)

| ***Study characteristics*** | |
| --- | --- |
| Methods | Design: Randomized Controlled Trial  Objective: To assess if the prescription of a gamified mindfulness meditation application would decrease college students’ reported symptoms of depression.  Data collection: We used a variety of IRB approved emails, flyers, and social media posts to recruit college students enrolled in a North Carolina university. The recruitment materials disbursed to potential participants included a Qualtrics survey link, which connected students to the IRB approved study webpage that detailed the purpose of the study, requirements, instructions for enrollment, and screened those wishing to participate for inclusion to the study.  Study participants completed two virtual sessions, separated by a two-week intervention period. During the initial session, participants were provided the informed consent and baseline assessments to gather demographics and depression severity scores.  Participants who met the inclusion criteria and consented, advanced to the next study page to begin the study. Participants first completed the demographics section, the PHQ-9, and were then randomly assigned to an experimental or control group.  Time 1 = baseline assessment & Time 2 = post 2-week study period. |
| Participants | Setting: in a North Carolina university  Eligibility Criteria: : (a) at least 18 years of age, (b) currently enrolled as a university student, (c) spoke English for a minimum of five years, and (d) not actively practicing meditation.  Characteristics: We recruited and randomly assigned 72 college students to an experimental group (n = 33) or a control group (n = 39).. Demographic data revealed the study sample to be 96% female (Mage = 21 years, age range: 18-48 years), an undergraduate student (96%), and reported moderately severe depression as evidenced by the PHQ-9. Specifically, the experimental group (n = 33) was mostly female (94%), an undergraduate student (94%), and experiencing moderately severe depression (MPHQ-9 = 15.33, range: 10-26); likewise, the control group (n = 39) was mostly female (97%), an undergraduate student (95%), and experiencing moderately severe depression (MPHQ-9 = 16.36, range: 9-28). The experimental group screenshot submissions from the Headspace application revealed that sessions ranged from three to 10 minutes with an average session lasting seven minutes. |
| Interventions | Headspace Application: an app that educates users how to perform mindfulness meditation, provides guided meditations, and provides free access to 10 minute meditation sessions over 14 days. |
| Outcomes | Depression Severity: 9-item 4-point scale Patient Health Questionnaire |
| Notes | Funding: The authors received no financial support for the research, authorship, and/or publication of this article.  Conflict of interest: The authors declared no potential conflicts of interest with respect to the research, authorship, and/or publication of this article |

[Fitzpatrick 2017](#STD-Fitzpatrick-2017)

| ***Study characteristics*** | |
| --- | --- |
| Methods | Design: RCT  Objective: Aim: to assess the feasibility of delivering CBT in a conversational interface via an automated bot in a way that facilitates engagement and reduction in symptoms  Hypotheses: We hypothesized that conversation with a therapeutic process- oriented conversational agent would lead to greater improvement in symptoms relative to the information control group  We also hypothesized that receiving psychoeducational material in a conversational manner would be more acceptable to those who received it  Data collection: Potential participants were recruited using a flyer posted on social media websites targeting a US university community for students who self-identified as experiencing symptoms of depression and anxiety  Baseline and 2 weeks (T2) |
| Participants | Setting: Unnamed university, New York, USA  Eligibility Criteria: self-identify as having symptoms of anxiety and  Depression; Inclusion criteria included age 18 and over (screened at the first level via checkbox confirmation) and able to read English (implied)  Characteristics: Participants were an average of 22.2 years old (SD 2.33) and over two-thirds female. Participants were mostly non-Hispanic (93%, 54/58), 79% Caucasian (46/58), with 7% (4/58) Asian, 9% (5/58) more than one race, 2% (2/58) African American, and 2% (2/58) Native American/Alaskan Native  In terms of baseline characteristics, nearly half (46%, 32/69) of the sample was in the moderately-severe or severe range of depression at baseline as measured by the PHQ-9, while three-quarters (74%, 52/70) were in the severe range for anxiety |
| Interventions | Woebot Application: an automated conversational agent designed to deliver CBT in the format of brief, daily conversations and mood tracking. Woebot is used within an instant messenger app that is platform agnostic and can be used either on a desktop or mobile device. |
| Outcomes | Frequency and severity of depressive symptomatology: The Patient Health Questionnaire PHQ-9 (score from 0 to 20 with higher scores indicating more severity)  Frequency and severity of anxious thoughts and behaviours: The Generalized Anxiety Disorder GAD-7 (score from 0 to 21 with higher scores indicating more severity)  Positive and negative effects (emotions): The Positive and Negative Affect Schedule PANAS (score from 10 to 50 with higher scores indicating more extreme affects)  Acceptability (mixed methods): a 5-point Likert scale assessing overall satisfaction, satisfaction with content, extent of emotional awareness, and learning experience, as well as comments about experiences with the application |
| Notes | Funding: NR  Conflict of interest: The second author (AMD) is the founder of a commercial entity Woebot Labs Inc. (formerly, the Life Ninja Project) that created the intervention (Woebot) that is the subject of this trial and therefore has financial interest in that company. Woebot Labs Inc. covered the cost of participant incentives, though Stanford made the payments  Other: Feasibility study, thus their effectiveness findings are preliminary |

[Flett 2019](#STD-Flett-2019)

| ***Study characteristics*** | |
| --- | --- |
| Methods | Design: Pre-registered three-arm RCT  Objective: To test the effect of two mobile mindfulness apps (Headspace and Smiling Mind) on changes in mental health, relative to a control app (Evernote)  Data collection:  Participants were recruited from April–August 2015 through the Department of Psychology’s online psychology research participation pool where research participation could be applied to a small component of their undergraduate Psychology course grade.  At baseline, 10-days post intervention, and 30-days post interventions |
| Participants | Setting: Psychology Laboratory in University of Otago, Dunedin, New Zealand  Eligibility Criteria: Participants need to be undergraduate students at the University of Otago between ages 18-49 2. Registered a recruitment target of 80 participants per condition  Characteristics: Gender (Female): Overall n=146 (70.2%), HS n=48 (66.7%), SM n=45 (71.4%), control n=53 (72.6%). Age: Overall M=20.08 (SD 2.88), HS M=20.19 (SD 3.05), SM M=20.24 (SD 3.95), control M=19.82 (SD 1.09). Ethnicity (Caucasian): Overall n=162 (77.9%), HS n=59 (81.9%), SM n=48 (76.2%), control n=55 (75.3%). |
| Interventions | 1) Headspace Application; Headspace is a mindfulness meditation application that provides guided and unguided mindfulness meditations. Headspace uses a variety of formal meditation practices such as, mindful breathing, body scan, sitting meditation, practice of non-judgment of thoughts and emotions, and other guided meditations that vary in orientation.  2) Smiling Mind Application: app offers guided and unguided mindfulness meditation practices |
| Outcomes | Depressive symptoms: assessed using the 20-item Center for Epidemiological Studies Depression Scale  Anxiety: assessed using the Hospital Anxiety and Depression Scale–Anxiety Subscale  Stress: assessed using the 10-item Perceived Stress Scale |
| Notes | Funding: This study was approved by the University of Otago Human Ethics Committee  Conflict of interest: The authors declare that they have no conflict of interest. |

[Flett 2020](#STD-Flett-2020)

| ***Study characteristics*** | |
| --- | --- |
| Methods | Design: Two-arm pragmatic, randomized, waitlist-controlled trial; Pragmatic RWCT  Objective: Primary objective: To examine whether access to a mindfulness meditation app in semester 1 would be associated with improvements in psychological distress at the beginning of semester 2, as compared to a waitlist.  Secondary objective: To examine whether there would be a dose-response relationship between app use and psycholo  Data collection:  Participants were recruited by email during March 2017. 2. Two residential colleges provided the contact details of their residents after the researcher (JF) described the study to students for two minutes at a start of year assembly.  At baseline i.e beginning of semester 1 (Time 1), At the beginning of semester (Time 2), and at the end of the academic year (Time 3) |
| Participants | Setting: College dormitories, University of  Otago, New Zealand  Eligibility Criteria: 1. Participants had to be first-year students residing in one of two residential colleges and had to be at least part-time University of Otago students.  2. Participants also had to own an iOS or android smartphone or be willing to use the Headspace desktop version on their personal computers or laptops.  Characteristics: The sample's overall mean age was 17.87 (SD 0.47); intervention M=17.89 (SD 0.48), control M=17.86 (SD 0.47). 67 participants in the intervention group (73.6%), and 71 participants in the control group (68.3%) were females. 103 participants in the intervention group (83.1%) and 99 participants in the control group (78.6%) were of European "New Zealandan" ethnicity. |
| Interventions | Headspace Application; Headspace is a mindfulness meditation application that provides guided and unguided mindfulness meditations. Headspace uses a variety of formal meditation practices such as, mindful breathing, body scan, sitting meditation, practice of non-judgment of thoughts and emotions, and other guided meditations that vary in orientation. |
| Outcomes | Psychological distress: assessed using the 10-item Kessler Psychological Distress Scale  College adjustment; assessed using the 19-item College Adjustment Test |
| Notes | Funding: This research was funded by the Office of the Vice-Chancellor, University of Otago [no grant number; 2015].  Conflict of interest: The authors declare that there are no conflicts of interest with respect to the authorship or the publication of this article. |

[Franklin 2016](#STD-Franklin-2016)

| ***Study characteristics*** | |
| --- | --- |
| Methods | Design: RCT  Objective: To test whether using a Therapeutic Evaluative Conditioning (TEC) app would display reductions in self-cutting and overall non-suicidal self injury (NSSI).  To examine the effect of active Therapeutic Evaluative Conditioning (TEC) dosage on self-injurious thoughts and behaviors (SITBs) and whether any treatment effects persisted during the month after TEC access ended  Data collection: Recruitment took place using online web forums primarily devoted to the discussion of topics related to self-injury or psychopathology. Investigators recruited from a total of nine forums.  at baseline, weekly during the treatment month, and at the end of the posttreatment month  at baseline, weekly during the treatment month, and at the end of the posttreatment month |
| Participants | Setting: Recruitment, allocation, and assessment took place online  Eligibility Criteria: To be eligible to participate, individuals had to meet the following criteria: (a) 18 years of age or older; (b) English fluency; and (c) two or more episodes of self-cutting in past month  Characteristics: The majority of participants were young adults (M = 22.91 years old; SD = 4.99) and the majority identified as Caucasian (83.21%), with the remaining identifying as Asian (5.34%), Hispanic (3.82%), Native American (1.53%), or Other (6.12%). Most participants were living in the United States (n = 107), with other participants living in Canada (n = 1), Europe (n = 19), Asia (n = 1), Africa (n = 1), and Australia (n = 2). |
| Interventions | TEC Application: a brief, game-like treatment that could be accessed by any device with an Internet connection. It takes 1 to 2 min to complete a single instance of TEC and TEC becomes more challenging as the trials progress. |
| Outcomes | Presence, frequency, and characteristics of self-injurious thoughts and behaviors: measured using the Self-Injurious Thoughts and Behaviors Interview  Emotional reactivity: measured using the Emotion Reactivity Scale  Psychological distress: measured using Brief Symptom Inventory (BSI)  Engagement in dysregulated behaviours: measured using the Index of Dysregulated Behaviors  Implicit aversion to Non-suicidal self injury behaviours: measured using the Affect Misattribution Procedure |
| Notes | Funding: Not reported  Conflict of interest: Not reported |

[Gajecki 2014](#STD-Gajecki-2014)

| ***Study characteristics*** | |
| --- | --- |
| Methods | Design: 3-arm RCT  Objective: To investigates the effects of two Swedish-language smartphone apps with real-time eBAC calculation and feedback among university students with established levels of risky drinking  Data collection: The student unions at Stockholm University and the Royal Institute of Technology in Stockholm, Sweden, provided the research group with e-mail addresses for their current members. Investigators e-mailed study information and a web page link to all addresses on the lists provided.  At baseline and 7 weeks post intervention |
| Participants | Setting: Recruitment, allocation and assessment took place online  Eligibility Criteria:1.Members of the student unions at Stockholm and the Royal Institute of Technology in Stockholm at the time  2.Having a smartphone running either iOS or Android  3. Participants with an AUDIT score indicating at least hazardous consumption (≥6 for women and ≥ 8 for men)  Characteristics: Gender (Females): Promillekoll n=309 (48.1%), PartyPlanner n=343 (53.7%), control n=346 (53.5%); (Males): Promillekol n=334 (51.9%), PartyPlanner n=296 (46.3%), control n=301 (46.5%). Age: Promillekol M=24.64 (SD 4.99), PartyPlanner M=24.82 (SD 4.63), control M=24.70 (SD 4.80). |
| Interventions | 1) Promillekoll Application: The user can register his/her alcohol consumption in real time, where the app displays the user's current eBAC. The application also offers a number of specific strategies to maintain alcohol consumption at a level that is not harmful—in this case, 0.06 percent BAC. The application warns the user if the drink entered will result in an eBAC over 0.06 percent and only displays values up to 0.08 percent. It also provides information  texts on alcohol and BAC.  2) Party planner Application; This app has a functionality of simulating or planning a drinking event beforehand and then comparing the simulation to the real-time event afterwards. The app user would then be able to pace his or her drinking based on a more realistic view of the amount of alcohol actually corresponding to a certain eBAC level. |
| Outcomes | Quantity and frequency of alcohol consumption: assessed using the Daily Drinking Questionnaire (DDQ)  Estimated blood alcohol concentration (eBAC): calculated based on the values from the Daily Drinking Questionnaire in conjunction with weight and gender for each individual.  Alcohol consumption and signs of harm and dependence in relation to alcohol: measured using the AUDIT scale |
| Notes | Funding: This study was funded by the Alcohol Research Council of the Systembolaget, the Swedish Research Council, and the Center for Psychiatric Research at Karolinska Institutet. One of the interventions in this study was developed by the Systembolaget and is publicly available free of charge in iPhone and Android versions Conflict of interest: None declared |

[Hammond 2015](#STD-Hammond-2015)

| ***Study characteristics*** | |
| --- | --- |
| Methods | Design: Open-label, randomized, parallel-group clinical  Trial; RCT  Objective: To determine if the use of an electronic medication reminder app, delivered through a smartphone device, was effective for increasing adherence to antidepressant medications in depressed college students  Data collection: Participants were recruited using campus based advertisements, flyers, and the university research recruitment system  At baseline and 25-35 days after completion of the initial visit |
| Participants | Setting: Unidentified state-funded institution in Ohio, US  Eligibility Criteria: English-speaking participants between the ages of 18 and 30 years who were prescribed  an antidepressant by a physician and had an Android or iPhone smartphone.  Exclusion criteria  Known cognitive impairment due to traumatic brain injury, stroke, or other condition that rendered them unable to read  and comprehend informed consent or the survey materials.  Characteristics: The majority of participants were white females, with an average age of 20.6 § 4.3 years and in their first or second year of college. Current smoking was reported by 26.7% of participants, and 78.9% of reported drinking alcohol. A diagnosis of depression was endorsed by 89.4% of participants taking antidepressants, 10.5% reported a diagnosis of anxiety, 1.8% reported a diagnosis of bipolar disorder, and 3.5% did not know their diagnosis. The average BDI score for participants upon entering the study was 19.8 +/-11.7, which corresponds to moderate depression severity |
| Interventions | Electronic medication reminder Application: Participants were required to use the medication |
| Outcomes | Depressive symptoms: assessed with the Beck Depression Inventory  Perceived Stress: assessed with the Perceived Stress Scale |
| Notes | Funding: This research was supported by the Initiative for Clinical  and Translational Research at Kent State University Conflict of interest: None declared |

[Hides 2018](#STD-Hides-2018)

| ***Study characteristics*** | |
| --- | --- |
| Methods | Design: RCT  Objective: To determine the 1-month efficacy and 2, 3 and 6 month outcomes of the Ray's Night Out app, which aims to increase alcohol knowledge and reduce alcohol use in young people  Data collection: Recruitment was via university student emails, youth relevant websites (e.g., reachout.com) and snowballing techniques  At baseline, 1,2,3 and 6 months |
| Participants | Setting: All baseline and follow-up surveys were completed online.  Eligibility Criteria: Australian residents aged 16–25 years who drank alcohol at least monthly and had an iPhone  Characteristics: Age: Intervention M=20.4 (SD 2.2), control M=20.5 (SD 2.5). Gender (Female): n=76 (79.2%), control n=77 (76.2%). English language fluency: Intervention n=76 (79.2%), control n=80 (79.2%). Education (Postgraduate): Intervention n=35 (36.5%), control n=37 (36.7%). Certificate or diploma: Intervention n=10 (10.4%), control n=15 (14.9%). High school: Intervention n=51 (53.1%), control n=49 (48.5%). |
| Interventions | Ray's Night Out Application: the app invites young people to take Ray, a red panda avatar, on a ‘relaxed’ ‘fun’ or ‘crazy’ virtual night out. It aims to provide users with the information, motivation and behavioral skills to set a drinking goal for the night by keeping Ray below a line for drinking. To increase motivation to take good care of Ray, users receive ‘good vibe’ points, which unlock photo booth rewards for using protective behavioral strategies. |
| Outcomes | Alcohol knowledge: measured using items adapted from CLIMATE Schools studies and the School Health and Alcohol Harm Reduction Project  ‘Patterns of Alcohol’ index  Frequency of risky single occasion drinking: measured using a standard questionnaire  Typical number of standard drinking units consumed on one occasion: measured using a standard questionnaire  Maximum quantity of standard drinking units consumed on one occasion: measured using a standard questionnaire  Frequency of alcohol-related problems: measured using the Rutgers Alcohol Problem Index  Harmful or problematic alcohol use: measured using the Alcohol Use Disorders Identification Test |
| Notes | Funding: The Young and Well Cooperative Research Centre (Young and Well CRC; 2011–2016) funded this project.  Conflict of interest: None declared |

[Hides 2019](#STD-Hides-2019)

| ***Study characteristics*** | |
| --- | --- |
| Methods | Design: RCT  Objective: To evaluate a new app called Music eScape, developed to assist young people with identifying, expressing, and managing emotions using music from their own music library  Data collection:(including timepoints for analysis)  Recruitment was via student emails and posters in 2 large universities and snowballing techniques. The advertisements invited young people (aged 16-25 years) who owned an iPhone and felt stressed to participate in a study testing a new mood management app. They did not include any mention about music in an attempt to avoid recruiting a selective sample of participants with a high affinity to music.  At baseline, 1, 2, 3, and 6 months |
| Participants | Setting: Two large Australia universities  Eligibility Criteria: Participants were Australian residents aged 16 to 25 years, who reported at least mild distress in the past month on the Kessler 10 Psychological Distress scale (K10>17) and had an iPhone.  Characteristics: Mean age 19.9 (SD 2.5), 134 individuals were females (79.3%), and majority were in high school (61.6%). 50.3% were in a relationship compared to 47.9% who reported being single. |
| Interventions | Music eScape Application: the app allows users to create a moodmap of the music stored on a user’s phone by tagging each song with a mood. Users then have the opportunity to review the tags assigned by the app. Music eScape then creates playlists for different moods and situations, such as “cheer up,” “wake up,” and “focus.” After completing their mood journey, users are asked to reflect on their current mood and rate the effectiveness of the playlist they just experienced |
| Outcomes | Emotion regulation: measured with the short-form of the Difficulties in Emotion Regulation Scale  Psychological distress: measured with The K10 scale  Mental well-being: measured with the Mental Health Continuum-Short Form |
| Notes | Funding: The Young and Well Cooperative Research Centre (Young and Well CRC; 2011-2016) funded this project. These funders had no role in the study design; collection, analysis, or interpretation of data; writing the manuscript; and the decision to submit the manuscript for publication.  Conflict of interest: none declared |

[Huberty 2019](#STD-Huberty-2019)

| ***Study characteristics*** | |
| --- | --- |
| Methods | Design: RCT with wait-list control  Objective: to test the efficacy of mindfulness meditation intervention delivered via a consumer-based mobile app (ie, Calm) as compared to a wait-list control group on stress in college students with elevated stress  Data collection:(including timepoints for analysis)  Participants were recruited between January and April 2018 via social media (ie, Facebook and Instagram), email listservs, and flyers and by emailing university professors; Used sample size calculation based on previous studies |
| Participants | Setting: Southwestern USA; Arizona State University  Eligibility Criteria:  Inclusion: (1)Current full-time undergraduate student attending a university in the Southwestern United States and (1) at least 18 years old, (2) scored ≥14 points on the PSS, (4) owned a smartphone, (5) were willing to download the Calm app, (6) were willing to be randomized, and (7) were able to read and understand English. Exclusion: (1)current mindfulness practice (ie, practice ≥15 min per day of meditation, yoga, and body scan within the past 6 months (2) were currently using the Calm app or another meditation app  Characteristics: Intervention: n= 41 (1)Age = 20.41 (2) Female = 36 (3) Freshman=10; Sophomore=12; Junior= 10; Senior=9 (4)Hispanic =13;Non-hispanic= 27; no response= 1 (5) Cuacasian = 25; Asian =6; Black =1; Biracial = 3; Other = 3; no response = 3 (6) Mental diagnosis = 12; No mental diagnosis = 29 (7) Medications =5; No medications =36 (8) Counselling=2; No counselling =39 Control: n= 47 (1)Age = 21.85 (2) Female = 43 (3) Freshman=17; Sophomore=10; Junior= 12; Senior=8 (4)Hispanic =7; Non-hispanic= 39; no response= 1 (5) Cuacasian = 23; Asian =9; Black =4; Biracial = 7; Other = 2; no response = 2 (6) Mental diagnosis = 11; No mental diagnosis = 36 (7) Medications =6; No medications =41 (8) Counselling=6; No counselling =41 |
| Interventions | Calm mobile Application: mindfulness meditation mobile app that offers a range of mindfulness meditation practice guide modules that vary in length, instruction, and content. Calm also integrates some CBT techniques into the meditation sessions on occasion. |
| Outcomes | Perceived stress: measured with PSS scale  Mindfulness: measured with Five Factor Mindfulness Questionnaire.  Self compassion: measured with Self-Compassion Survey Short-Form |
| Notes | Funding: Not included  Conflict of interest: JH (principal investigator) is currently the Director of Science at Calm (although she was engaged in this role almost 1 year after the design, collection  of data, and analysis of results of the study presented in this paper). |

[Hur 2018](#STD-Hur-2018)

| ***Study characteristics*** | |
| --- | --- |
| Methods | Design: RCT  Objective: to examine the hypothesis that this app-based CBT program will reduce clinical symptoms compared to a mood diary group; to assess whether this scenario-based platform results in modifying the users’ dysfunctional beliefs through reappraisal and re-constructing.  Data collection:(including timepoints for analysis)  Voluntary : Participants were initially recruited through web-based advertisements, posters, and outpatient psychiatric clinics.  At baseline and 3 weeks (post intervention) |
| Participants | Setting: 2 universities in Seoul, Korea  Eligibility Criteria:  Inclusion: (1) the DSM-5 diagnosis for Other Specified Depressive  Disorder, (2) fluent in Korean, (3) 12 or more years of formal  education, and (4) familiar with Internet and smartphone app  use familiarity.  Exclusion: (1) high risk of suicide attempt (2) diagnosis of psychosis or major de- pressive disorder based on the Structured Clinical Interview for the DSM-IV, Non-Patient Edition,(3) current psychiatric  drug use during at least a 4-week period before participating in the program, (4) and/or family history of psychiatric disor-der.  Characteristics:  Baseline Total: n = 34 (1) Age = 23.71 (2) Female =2 (3)Beck = 24.12 Intervention Group: n = 17 (1) Age = 24.76 (2) Female = 15 (3) QOL = 98.62 (4) Dysfunctional Attitude = 148.76 (5) BDI-II = 22.65 (6) Trait Anxiety = 57 (7) Self-esteem = 27.65 Control Group: n = 17 (1) Age = 22.65 (2) Female = 15 (3) QOL = 90 (4) Dysfunctional Attitude = 165.82 (5) BDI-II = 25.59 (6) Trait Anxiety = 60.82 (7) Self-esteem = 27.59 |
| Interventions | Todac Todac mobile cognitive behavioural therapy (CBT) Application: The app was developed with focus on targeting dysfunctional beliefs in individuals at high risk of psychiatric illness or with psychiatric disorders. Users were presented with scenarios following 3 steps: Step I: identifying, Step II: ‘‘Decatastrophizing’’, Step III: ‘‘Distancing’’. After each step, users can assess others’ responses and advice. |
| Outcomes | Dysfunctional attitude scale: measured with Dysfunctional Attitude Scale  Depressive symptoms: measured with Beck Depression Inventory Scale  State-trait anxiety: measured with STAI-X2 (State-Trait Anxiety Inventory)  Self-esteem: measured with Rosenberg Self-esteem Scale |
| Notes | Funding: Korea Health Technology R&D Project through the Korea Health Industry Development Institute (KHIDI), funded by the Ministry of Health & Welfare, Republic of Korea  Conflict of interest: no competing financial interests exist |

[Kageyama 2021](#STD-Kageyama-2021)

| ***Study characteristics*** | |
| --- | --- |
| Methods | Design: Randomized Controlled Trial  Objective: To assess the preliminary efficacy of the Subliminal Priming with Supraliminal Reward Stimulation,, a smart phone application intervention for people with subthreshold depression.  Data collection:(including timepoints for analysis)  Details on the recruitment of participants are published in the protocol paper (Kato et al., 2020). The participants were recruited through Kibi International University in Okayama Prefecture, Japan.  Baseline and post-intervention (5 weeks) |
| Participants | Setting: Kibi International University in  Okayama Prefecture, Japan  Eligibility Criteria:  1) 18 years and older 2) Center for Epidemiologic Studies Depression Scale (CES-D) score ≥ 16 (Shima, Shikano, Kitamura, & Asai, 1985) 3) Owns a smartphone with an iOS® operating system 4) Written informed consent prior to participation  Characteristics:  Total: n= 32  Mean Age: 20.1  Gender: 34.4% female, 65.6% male  Other Criteria: subthreshold depression |
| Interventions | SPSRS Application: an app designed to improve depressive symptoms in people with subthreshold depression by presenting positive word stimuli through videos. The app is also programmed to display videos that feature general confidence-boosting words. |
| Outcomes | Depressive Symptoms: measured with 20-item Center for Epidemiologic Studies Depression Scale  Psychological Distress: measured with 6-item Kessler Screening Scale for Psychological Distress |
| Notes | Funding: This work was supported by JSPS KAKENHI Grant Number 19K19724.  Conflict of interest: None of the authors have any conflicts of interest to declare. |

[Kauer 2012](#STD-Kauer-2012)

| ***Study characteristics*** | |
| --- | --- |
| Methods | Design: RCT  Objective: to investigate, the utility of the mobiletype  program as a first-step intervention program. The primary  hypothesis was that young people who completed the mobiletype  intervention program would have lower depressive symptoms  than those who completed the attention comparison program.  Data collection:(including timepoints for analysis)  Attention-controlled study with balanced (1:1) individual randomisation into parallel-groups; GPs were trained and recruited patients from 10 different GP clinics and screened their patients for eligibility and organized an appointment for interested participants with a research assistant using an online booking form, a faxed referral form, or by phone."  (1) pretest, (2) posttest, and (3) 6-week follow-up |
| Participants | Setting: Primary Care rural and urban areas; Goulburn Valley Region and Albury-Wodonga; Region + Melbourne; This study was  conducted in Victoria, Australia  Eligibility Criteria:  Inclusion: (1) be aged between 14 and  24 years, (2) speak proficient English, and (3) have a mild or  more severe emotional or mental health issue as assessed by  their GP or indicated by a score greater than 16 on the Kessler  Psychological Distress Scale  Exclusion: Participants were excluded  if their psychiatric or medical condition prevented them from  complying with either the requirements of informed consent or  the study protocol (ie, current psychosis or imminent  hospitalization).  Characteristics:  Intervention: n= 69 (1) Age = 18.5 (2) Male =15 (3) Ethnic identification = 10 (4) Employed =18; Unemployed= 9; Student = 41 (5)Ever had alcohol=59; Ever been drunk = 52; Ever had cigarette=38; Ever had marijuana= 33; Every used other drug=26 (6) Depression= 20.4; Anxiety= 14.1; Stress =20.3  Control: n =49 (1) Age = 17.4 (2) Male =17 (3) Ethnic identification = 4 (4) Employed =7; Unemployed= 4; Student = 35 (5)Ever had alcohol=38; Ever been drunk = 31; Ever had cigarette=25; Ever had marijuana= 18; Every used other drug=10 (6) Depression= 19.4; Anxiety= 11; Stress =16.9 |
| Interventions | Mobiletype Application: App allowed users to self-monitor 8 areas of functioning including current activities, location, companions, mood, recent stressful events, responses to stressful events, alcohol use, cannabis use, quality and quantity of sleep, quantity and type of exercise, and diet. |
| Outcomes | Depressive Symptoms: measured with Depression Anxiety Stress Scale  Emotional Self-awareness (ESA): measured with the ESA Scale, Self-reflection and Insight Scale, Ruminative Response Scale, and the Meta-Evaluation Scale. |
| Notes | Funding: Research grants from the Telstra Foundation and The Shepherd Foundation, and the Victorian Government’s Operational Infrastructure Support Program supported infrastructure.  Conflict of interest: Not mentioned |

[Kazemi 2020](#STD-Kazemi-2020)

| ***Study characteristics*** | |
| --- | --- |
| Methods | Design: Randomized Controlled Trial  Objective: To examine the efficacy of a theoretically based mHealth app for alcohol intervention.  Data collection: In both studies, participants attended the same large, public university in the southeastern United States. The University’s Institutional Review Board approved both studies. Prior to participation, participants signed a consent form and were provided a unique identifier to ensure confidentiality. The studies ran simultaneously from August 2015 through July 2018. |
| Participants | Setting: Large public University in the Southeastern USA  Eligibility Criteria:  Inclusion criteria were similar across both studies: at least 18 years of age, alcohol consumption in the prior month, ability to communicate in English, and owning an iPhone.  Characteristics: Study 1 included 141 participants who completed the baseline survey. Demographic information for Study 1 participants including age, first drinking age, gender, and race are summarized in Table 2. There were no significant gender differences between the two groups. The mean age was 18.94 (SD ¼ 0.80) for participants for the in-person BMI group and 19.13 (SD ¼ 2.55) for those in the app-based BMI þ SP group. The majority of the mandated participants in the in-person BMI group and the BMI þ SP app group were men (60% and 59%, respectively) and Caucasian (79% and 76%, respectively).  Study 2 included 157 participants in the AO control group. All participants finished the baseline survey, and 101 participants completed the 6-week survey. The BMI þ SP app group had 81 participants. All finished the baseline survey, and 69 participants completed the 6-week survey. The mean age of the two groups was 19.87 (SD ¼ 2.84) and 19.84 (SD ¼ 3.10), respectively. |
| Interventions | BMI + SP Application: the app incorporated many features aimed at motivating users to change drinking habits via interactive tools. The app collected data, tracked behavior, provided education, and offered incentives for behavioral changes. Key features included: My Coach, Personalized Feedback, Strategies, Know Your BAC, Daily Log, Learn More, and Where to Go. The app also sent messages to participants aimed at behavior change, provided personalized normative feedback, and offered strategies to promote healthy drinking behavior. |
| Outcomes | Alcohol consumption: measured with (1) alcohol use disorders identification test (AUDIT) 10-item questionnaire (2) daily drinking questionnaire (3) young adult alcohol consequences questionnaire  Motivation to Change: measured withreadiness to change questionnaire |
| Notes | Funding: This research was supported by the Agency for Healthcare Research and Quality (AHRQ) Grant 1R21 HS023875-01. The content is solely the responsibility of the authors and does not necessarily represent the official views of AHRQ, the National Institutes of Health, the U.S. Department of Veterans Affairs, or the United States government. Conflict of interest: The authors report no conflicts of interest. The authors alone are responsible for the content and writing of this article |

[Kenny 2020](#STD-Kenny-2020)

| ***Study characteristics*** | |
| --- | --- |
| Methods | Design: Multicentre cluster RCT  Objective: To test the effectiveness of CopeSmart in improving self-management through emotional self- monitoring and the use of positive coping strategies  Data collection:(including timepoints for analysis)  consent was received from students, no details as to how information of study was given  Baseline, Time 2: 4-5 weeks after baseline, Time 3: 8-10 weeks after Time 2 |
| Participants | Setting: Grades 4-6 in Ireland. Students used app in their personal environment; secondary schools  Eligibility Criteria:Inclusion: student in 4th to 6th year ; Exclusion: failure to return parental consent  Characteristics:N=560 n(int)= 385 ; n(control)=175  For the Overall Sample:  1. Age: mean=16.05; SD=0.76  2. Female: 349 (62%)  3. Ethnicity: 535 (96%)=white vs. 4% other  4. Nationality: 39 (7%) = Foreign national  5. School disadvantage status: DEIS (delivering equal opportunity school)=199 (36%)  6. Highest level of mother education: college (270; 48%) vs. leaving certificate, junior certificate  7. Highest level of father's education: college (239 ; 43%) vs. leaving certificate, junior certificate  other characteristics: currently seeing a healthcare professional; school gender mix |
| Interventions | CopeSmart Application: a mental health mobile app which promotes self-management through emotional self- monitoring and the use of positive coping strategies. Allows users to rate how happy, angry, sad, stressed or worried they have felt on a scale of 1–10. |
| Outcomes | Emotional Self Awareness (ESA): measured with The Emotional Self-Awareness Scale  Psychological Distress: measured with the Depression Anxiety and Stress Scale-21 |
| Notes | Funding: Irish Research Council Conflict of interest: N/A |

[Lahtinen 2020](#STD-Lahtinen-2020)

| ***Study characteristics*** | |
| --- | --- |
| Methods | Design: Randomized Controlled Trial  Objective: To evaluate the app, Welzen against. a control treatment on well being of university students  Data collection:(including timepoints for analysis)  Participants were recruited from among the faculty, staff, and students of the University of Turku. The University Wellbeing Services helped in the recruitment. The study was advertised via the university email lists, in the intranet, and with fliers distributed in the campus area.  Baseline and 4 weeks |
| Participants | Setting: University of Turku, Finland  Eligibility Criteria:  Every volunteer was accepted to participate provided they (1) owned a smartphone, (2) had sufficient English skills (the app was in English), (3) had not practiced mindfulness on a regular basis, and (4) was committed to regular practice for four weeks. Volunteers with a psychiatric diagnosis were instructed to consult their therapist/ doctor about the suitability for participation. If permitted by the therapist, they were allowed to take part in the study.  Characteristics: Faculty, staff and students from the university of Turku |
| Interventions | Welzen Application: an app that consists of a guided mindfulness program targeted at reducing symptoms of psychological stress, anxiety and depression as well as increasing mindfulness skills.The app focused on recognizing bodily signs of psychological stress, attention to breathing, enhancing patience and strengthening self-compassion via a loving-kindness meditation. |
| Outcomes | Perceived Stress: measured with Perceived Stress Scale 10 item questionnaire  Anxiety: measured with Generalized anxiety disorder questionnaire  Depressive Symptoms: measured with Beck Depression Inventory questionnaire |
| Notes | Funding: Open access funding provided by University of Turku (UTU) including Turku University Central Hospital. Conflict of interest: A4 developed the studied apps. Other authors declare no other potential conflicts of interest with respect to the research, authorship, and/or publication of this article. |

[Lee 2018](#STD-Lee-2018)

| ***Study characteristics*** | |
| --- | --- |
| Methods | Design: RCT  Objective: to evaluate a mindfulness-based app’s (“DeStressify”) efficacy on stress, anxiety, depressive symptomatology, sleep behavior, work or class absenteeism, work or school productivity, and quality of life (QoL) among university students.  Data collection:(including timepoints for analysis)  Participants were recruited through poster advertisements, in-class announcements, and emails to administrative assistants of various faculties across the University of British Columbia (UBC) Okanagan campus. Individuals interested in participating in the study emailed the researcher assistant and received a link to the Web-based eligibility survey, consent form, and baseline survey  baseline, 4 weeks after intervention |
| Participants | Setting:Faculty of Health and Social Development, University of British Columbia, Kelowna, BC, Canada  Eligibility Criteria:  1. smartphone and internet access 2.Eligibility criteria included (1) enrollment in full course load during the winter term at the UBC Okanagan campus in an undergraduate program, (2) ownership of a smartphone, (3) regular access to the Internet, and (4) fluent comprehension of the English language  Characteristics:  CONTROL:  Age 16-47 (mean 20.9)  67% female  71% White, 11% Chinese 6% South Asian  14% with a mental health diagnosis  12% with mental health service use  EXPERIMENTAL:  Age 18-27 (mean 20.3)  58% female  65% white, 16% chinese; 12% South Asian  22% with a mental health diagnosis  13% with mental health service use |
| Interventions | DeStressify Application: The app contains a core plan that delivers mindfulness-based exercises through audio, video, or text files. |
| Outcomes | Perceived Stress: measured with Perceived stress scale  Anxiety: measured with State-Trait Anxiety Inventory for adults  Depressive Symptoms: measured with The Quick Inventory of Depressive Symptomatology Self-Report  Sleep Quality: measured with Pittsburgh Sleep Quality Index  Work Productivity: measured with Work Productivity and Activity Impairment Questionnaire |
| Notes | Funding: Not declared  Conflict of interest: N/A |

[Levin 2020](#STD-Levin-2020)

| ***Study characteristics*** | |
| --- | --- |
| Methods | Design: RCT  Objective: to  evaluate the feasibility and acceptability of a popular mindfulness meditation app (Stop, Breathe & Think) for students  on a college counseling center waitlist  Data collection:(including timepoints for analysis)  Voluntary sampling: Participants  were primarily recruited through flyers provided at CAPS by  counseling center staff  baseline, 2 weeks, 4 weeks after baseline |
| Participants | Setting: University students on the waitlist for the Counseling and Psychological Services center (CAPS) at a university in the Mountain West region of the United States  Eligibility Criteria: Inclusion Criteria: 1) 18 years of  age or older, 2) owning a smartphone (Android or iPhone),  3) current student at the university, and 4) currently seeking  treatment at CAPS with an expected wait time of at least  two weeks for services.  Characteristics: N=23 ; n(int)=10; n(control)=13  Age: 18 to 25 (M ¼ 20.43 years,  SD 2.46 years)  Sex: 100% female  Ethnicity: 87%  non-Hispanic, White (with 9% Hispanic, White and 4%  American Indian and White) |
| Interventions | Stop, Breathe & Think Application: The app provides guided meditations and mindfulness procedures. |
| Outcomes | Mental Health Symptoms: measured with Counseling center assessment of psychological symptoms- 34 item version  Mindfulness: measured with Five facet mindfulness questionnaire |
| Notes | Funding: N/A  Conflict of interest: N/A |

[Liu 2022](#STD-Liu-2022)

| ***Study characteristics*** | |
| --- | --- |
| Methods | Design: Unblinded Randomized Controlled Trial  Objective: To examine the superiority of a newly developed chatbot-delivered self-help depression intervention to a minimal level of bibliotherapy regarding (1) efficacy on depression symptoms reduction, (2) adherence, and (3) therapeutic alliance.  Data collection:(including timepoints for analysis)  Participants were recruited using an online poster from three different universities in China, respectively in the city of Harbin, Wuhan, and Guangzhou.  (baseline = T1, 4 weeks = T2, 8 weeks = T3, 12 weeks = T4, 16 weeks = T5) |
| Participants | Setting: the cities of Harbin, Wuhan, and Guangazhou, China  Eligibility Criteria: aged 18 years or older, being full-time university students, able to communicate in Chinese without difficulty, have skillful use of smartphones, have a PHQ-9 score of nine or higher, not currently undergoing any form of mental health intervention. The eligibility criterion of PHQ-9 score equal to or greater than nine was decided according to the average inclusion PHQ-9 score in previous depression trials  Characteristics: The recruitment started on February 11th and finished on April 15th, 2021. A total number of 187 responses were received, among which 83 participants (46 females) were considered eligible for the trial. All of the participants were university students (undergraduate students (n = 31) or postgraduate students (n = 52)). They were between 19 and 28 years old (mean = 23.08, SD12=1.76). All of them were native Chinese speakers. The trial started on April 17th, 2021, and lasted for 16 weeks, during which period participants were required to provide follow-up data every four weeks (baseline = T1, 4 weeks = T2, 8 weeks = T3, 12 weeks = T4, 16 weeks = T5). At T5, client satisfaction and working alliance were evaluated with CSQ-8 and WAI-SR. |
| Interventions | “XiaoNan” via WeChat:  A therapy chatbot (XiaoNan) was developed and deployed through the smartphone app “WeChat”. XiaoNan is a pipeline-based chatbot powered by the open-source conversational AI “RASA”. Therapeutic content and conversations were based on machine learning models. Chatbot responses were created according to the principles of CBT. |
| Outcomes | Depression: (PHQ-9) 9-items on a 4-point rating scale. Total score between 0 and 27.  Anxiety: (GAD-7) 7-item on a 0 to 3 point scale. Total score between 0 and 21.  Positive and Negative Affect Symptoms: (PANAS)  Two 10-item scales rated on a 5-point scale |
| Notes | Funding: This work did not receive any funding.  Conflict of interest:The authors declare that they have no known competing financial interests or personal relationships that could have appeared to influence the work reported in this paper |

[McCloud 2020](#STD-McCloud-2020)

| ***Study characteristics*** | |
| --- | --- |
| Methods | Design: A web-based randomized controlled trial  Objective: This study aims to evaluate for the first time the effectiveness of a self-guided mobile app, Feel Stress Free, for the treatment of depression and anxiety symptoms in students.  Data collection: A total of 4 universities that partnered with Thrive Therapeutic Software Limited agreed to take part: University College London (UCL), School of Oriental and African Studies University of London, University of Buckingham, and University of Roehampton. Students were recruited between March and June 2016 through their university student union or student welfare services via email, poster and social media advertisements, and university welfare staff recommendations. The recruited participants were directed to the Thrive website, where they could enroll by entering their university email address. Participant IDs were then provided via an email to this address, with a link to the web-based information sheet and consent form |
| Participants | Setting: Universities in England including: University College London,  School of Oriental and African Studies University of London, University of Buckingham and University of Roehampton, UK  Eligibility Criteria: Eligible participants were aged 18 years or over; scored 8 or above on one or both subscales of the Hospital Anxiety and Depression Scale (HADS), indicating at least a possible case of depression and/or anxiety [27]; were currently a student at 1 of the 4 partnered universities; had access to an Apple or Android phone or tablet or a computer with Firefox, Safari, or Chrome installed; and were computer and internet literate.  Characteristics: The mean age of all 168 participants was 24.3 years (SD 6.71; range 18-54 years), 82.7% (139/168) of the participants were female, and 61.9% (104/168) were undergraduate students. At baseline, the mean score on HADS-A was 13.7 (SD 3.33) and on HADS-D was 8.31 (SD 3.96). |
| Interventions | The Feel Stress Free Application: the app, uses CBT-based activities and comprises 4 behavioral relaxation activities: calm breathing, mindfulness-style meditation, deep muscle relaxation, and self-hypnosis; one cognitive activity, incorporating both mood tracking and thought challenging; a relaxing minigame; and a feature for positive messages in a bottle. |
| Outcomes | Depressive symptoms: HADS-Depression Subscale  Anxiety: HADS-Anxiety Subscale |
| Notes | Funding: TM is funded by the Economic and Social Research Council and the Medical Research Council.  Conflict of interest: Dr Andres Fonseca is a consultant psychiatrist and Co-Founder of Thrive Therapeutic Software Ltd, the company that developed the app. This trial was designed in collaboration with Dr Fonseca, and Thrive provided the app to participants for free, assisted with technical difficulties and contacting universities, and covered recruitment costs. Preparation of this manuscript was completed by the authors independently of Thrive, and Thrive did not have any involvement in review or approval of the final manuscript. The authors report no other conflicts of interest. |

[Newman 2021](#STD-Newman-2021)

| ***Study characteristics*** | |
| --- | --- |
| Methods | Design: The study used a randomized, no-treatment controlled design.  Objective: The goal of the current study was to conduct a pilot test of a smartphone-based guided self-help intervention for GAD  Data collection:(including timepoints for analysis)  Participants were recruited from flyers posted around Penn State University and Stanford University campuses and online bulletin boards for recruitment. The flyers invited individuals experiencing excessive worry, anxiety, or stress to participate in a study on a phone application and included links and QR codes to access a Qualtrics screening survey.  Baseline, Post treatment (3 months after baseline), Follow-up (6 months following post-treatment) |
| Participants | Setting: Penn State University and Stanford University in the USA  Eligibility Criteria: Meet diagnostic criteria for GAD  Characteristics: One-hundred undergraduate participants with selfreported GAD were randomized to either the guided self-help intervention (N = 50, 82% Female, Mage = 21.62, Age range = 18–42, 68% Caucasian/ White, 4% Arab/Middle Eastern/Arab American, 16% Hispanic/Latino, 20% Asian/Asian American, 2% Asian Indian, 2% Pacific Islander) or no treatment (N = 50, 72% Female, Mage = 21.18, Age range = 18–37, 62% Caucasian/White, 12% African American/Black, 2% American Indian/Alaska Native, 2% Arab/Middle Eastern/Arab American, 10% Hispanic/Latino, 10% Asian/Asian American, 8% Asian Indian, 2% Other). Note that, although all participants met full criteria for GAD at the prescreen, eight participants (N = 3 in intervention; N = 5 in control) no longer met full criteria by the baseline assessment. Because these participants still endorsed partial GAD criteria, they were retained in the study and all analyses unless otherwise noted. |
| Interventions | CBT intervention Application: the app had units that covered an introduction to anxiety, automatic thoughts, cognitive reframing, introduction to behavior change, imaginal exposure, situational exposure, mindfulness, and habit formation. Each session included psychoeducational lessons (e.g., information about logical errors), tools for skill practice (e.g., identifying one’s own logical errors), and regular anxiety check-ins.  Participants also had access to coaches, whose role included supporting and enhancing user motivation, monitoring progress, facilitating goal setting and offering accountability, providing feedback on technique usage. Coach messaging was done via a web-based “dashboard,” and delivered to users within the mobile application. |
| Outcomes | Anxiety: Generalized Anxiety Disorder Questionnaire for DSM-IV; The State-Trait Anxiety Inventory-Trait Version; Penn State Worry Questionnaire  Depression, Anxiety and Stress: Depression, Anxiety and Stress Scales-Short  Form |
| Notes | Funding: This work was supported by National Institute of Mental Health: [Grant Number 1R01MH115128- 01A1]; Stanford University School of Medicine’s Behavioral Medicine Lab: [Gift funds].  Conflict of interest: The fourth author of this manuscript was employed by Lantern (the guided self-help program used here) when this study was conducted but has no current financial ties to the company as she left her employment there. She was not involved in consenting participants, collection of outcome data, or data analysis of any sort. The first and last author were unpaid consultants for Lantern. After data collection was completed, Lantern ceased operations and thus nobody involved in the study has any financial interest in Lantern. |

[Orosa-Duarte 2021](#STD-Orosa_x002d_Duarte-2021)

| ***Study characteristics*** | |
| --- | --- |
| Methods | Design: single-blind, randomised controlled trial  Objective: To compare the effect of a mindfulness-based mobile application versus an in-person mindfulness-based training program in terms of reducing anxiety and increasing empathy, selfcompassion, and mindfulness in a population of healthcare students.  Data collection:(including timepoints for analysis)  Voluntary participants were recruited from the Autonomous University of Madrid. We informed the students during lecture time and via e-mail, and we offered course credits for completion of the training.  baseline and postintervention (8 weeks) |
| Participants | Setting: Autonomous University of Madrid, Madrid, Spain  Eligibility Criteria:  1. Students of Medicine, Psychology, Nursing, or Nutrition 2. Sign informed consent document  Characteristics: Of the remaining 84 students analysed, 71 (85%) were women, with a mean age of 23 years (SD ¼ 4.16). Most students were in their fourth year (n ¼ 24), followed by their second (n ¼ 19), sixth (n ¼ 17), fifth (n ¼ 14), third (n ¼ 8), and first (n ¼ 2) academic year. Most students were in years with clinical practices at health facilities (79%). Most (65%) participants were medical students, 15 (18%) were students of psychology, 11 (13%) came from the nursing school, and 3 (4%) were studying nutrition. |
| Interventions | ‘REM Volver a casa’ (‘Mindfulness-Based Emotion Regulation-Going Home’) Application: App is a training program with eight stages divided into three sections called Listening, Practicing, and Integration into everyday life. The app provides short videos with explanations about the fundamentals of mindfulness, self-compassion, and the physiological stress reaction, as well as audio segments that guide practices of mindfulness. |
| Outcomes | Anxiety: State-Trait Anxiety Inventory  Empathy: Jefferson Scale of Physician Empathy  Self-Compassion: Self-compassion scale  Mindfulness: Five Facet Mindfulness Questionnaire |
| Notes | Funding: This study was priced as a Teaching Innovation Project and an "IMPLANTA" Project of the Autonomous University of Madrid and was granted with a research assistant during the first year of the study. Conflict of interest: The authors report no conflicts of interest. The authors alone are responsible for the content and writing of the article. |

[O’Donnel 2019](#STD-O_x2019_Donnel-2019)

| ***Study characteristics*** | |
| --- | --- |
| Methods | Design: a single-blind, randomized controlled design  Objective: to evaluate the benefits and feasibility of a personalized alcohol harmminimization intervention delivered via smartphones.  Data collection:(including timepoints for analysis)  Participants were recruited via invitations on social media (e.g., Facebook), and from advertisements placed within a large metropolitan university campus.  Baseline and immediate post-intervention |
| Participants | Setting: Large metropolitan university campus in Australia  Eligibility Criteria:  Participants were eligible for the trial if they answered yes to the following criteria in the baseline survey: (a) aged 18–35 years, (b) access to an Int.J. Behav. Med. (2019) 26:401–414 403 iPhone, (c) reported being motivated to reduce alcohol use, and (d) consume alcohol, on average, at least once a week.  Characteristics:  Intervention :A total of 25 individuals aged between 18 and 35 years (18 females; Mage = 21.36 years, SDage = 4.15 years) completed the baseline assessment and were randomized to download the Minimise app (see Fig. 1). After downloading the app, three participants were lost to follow-up. This reduced the sample of participants who completed all phases of the intervention study (i.e., baseline and follow-up) to 22.  Control: A total of 20 individuals aged between 18 and 32 years (18 females; Mage = 22.75; SDage = 4.41) completed the baseline assessment and were randomized to download the InstantSurvey app (see Fig. 1). After downloading the app, four participants were lost to follow-up. This reduced the sample of participants who completed all phases of the control study (i.e., baseline and follow-up) to 16. |
| Interventions | ‘Minimise’ Application: App delivers protective behavioral strategies tailored to the users’ goals and drinking context |
| Outcomes | Alcohol consumption: measured with scales (1) examining consumption of alcohol (2) difficulties with work and/or study due to drinking (3) interpersonal difficulties due to drinking, and (4) physical health related to drinking |
| Notes | Funding: N/A  Conflict of interest: The authors declare that they have no conflict of interest. |

[Ponzo 2020](#STD-Ponzo-2020)

| ***Study characteristics*** | |
| --- | --- |
| Methods | Design: randomized, waitlist-controlled trial  Objective: To test the efficacy and sustained effects of using a mobile app (BioBase) and paired wearable device on anxiety and well-being in university students with elevated levels of anxiety and stress.  The study also examined sustained effects (at 6 weeks from baseline) of the intervention on anxiety and well-being.  Data collection: including timepoints for analysis  Participants were recruited using institutional participant pools at different UK universities as well as via social media, mailing lists, and flyers and through university staff. Recruitment took place between October and November 2019, and potential participants were screened for eligibility via a Qualtrics survey.  T0= baseline, T1= Week 2, T2= Week 4, T3= Week 6 |
| Participants | Setting: Universities in the United Kingdom  Eligibility Criteria: (1) being aged between 18 and 25 years, (2) having scored >14 points on the Depression, Anxiety and Stress Scale-21 items (DASS-21 [39]) stress subscale or >7 points on the DASS-21 anxiety subscale, (3) owning an iPhone 6 or above, (4) not having any previous psychiatric or neurological conditions, (5) not being pregnant at the time of testing, and (6) being able to read and understand English.  Characteristics:  Intervention (n=72):  Females= 45  Males=27  Age= 19.9 (1.83)  Dass, Anxiety= 15.39 (6.86)  Dass, Stress= 21.08 (7.02)  Waitlist control (n=74):  Females= 47  Males=27  Age= 19.84 (1.76)  Dass, Anxiety= 14.46 (7.23)  Dass, Stress= 19.86 (7.66) |
| Interventions | The BioBase Application: an app comprising psychoeducational content on mental health and well-being, mood tracking (via an ecological momentary assessment), and in-the-moment exercises (eg, deep breathing and relaxation techniques). |
| Outcomes | Anxiety: State-Trait Anxiety Inventory; Depression, Anxiety and Stress Scale-21 items  Well-being and psychological functioning: Warwick-Edinburgh Mental Well-Being Scale  Depression: DASS-21, Patient Health Questionnaire |
| Notes | Funding: N/A  Conflict of interest: DP was the CEO of BioBeats, the provider of the BioBase program. DM was the CTO of BioBeats, the provider of the BioBase program. SP, NH, JK, and GB were employees of BioBeats, the provider of the BioBase program. The company owners were not involved in the analysis, which was conducted by SP and reviewed by JK. |

[Reid 2011](#STD-Reid-2011)

| ***Study characteristics*** | |
| --- | --- |
| Methods | Design: Multi-centre, multi-regional, region stratified, single blind, attention-controlled trial  Objective: to investigate a number of suggested benefits of the mobiletype program.  Data collection:(including timepoints for analysis)  Recruitment of GPs: All general practitioners in the Goulburn Valley Region  and Albury/Wodonga Regions were invited to participate  in the study ; GPs in Melbourne were recruited via ( GPs screened their patients for eligibility and organized an appointment for interested participants with a research assistant using an online booking form, a faxed referral form, or by phone.)  the local Divisions of General Practice -- appears voluntary & purposeful; Recruitment of Youths: GPs screened their  patients for eligibility to the study, following this the youth met with a RA to go through consent, pre-test questionnaires etc. Parental consent was only sought if parent was present at initial appointment.  pre-test (baseline); post-test (2-4 weeks after mobiletype intervention) ; 6 weeks and 6 months (6 months data not included in this article) |
| Participants | Setting: 26 different practices  in the three recruitment areas: 12 in greater Melbourne, 7  in Albury/Wodonga and 7 in the Goulburn Valley (Primary Care, Australia)  Eligibility Criteria: Practitioners: All clinics in the Goulburn Valley Region and Albury/Wodonga Regions were invited. Clinics that listed an interest in adolescent health on the Melbourne General Practice Network were targeted  Patients (1) aged 14 to 24 years, (2) speak proficient English and (3) have a mild or more severe motional/mental health issue as assessed by their GP, or indicated by a K10 Symptom score greater than 16. Participants were excluded if they had a severe psychiatric or medical condition that prevented them from complying with either the requirements of informed consent or study protocol  Characteristics: 22.1% of the intervention group were males compared to 37%. The mean age for the intervention group was 18.5 (SD3.2) compared to the comparison group 17.4 years (SD3.2). Of n=111, 10 in the intervention group (22.7%) and 4 in the comparison group (9.1%) identified as coming from ethnic background. 60.3% of the intervention group and 76.1% of the comparison group were students, compared to 26.5% in the intervention group and 15.2% in the comparison group who reported employment. Table 2 reported substance use, with the majority of the study population reporting ever using alcohol, getting drunk, having a cigarette, trying marijuana. |
| Interventions | Mobiletype (V4) Application: the mobiletype app monitors a young person’s mood, stress, coping strategies and daily activities a number of times per day, and their eating, sleeping, exercise patterns, and alcohol and cannabis use once per day. This information is then uploaded to General Physicians, via a secure website and displayed in summary reports for review. |
| Outcomes | Depression, Anxiety, and Stress: measured with Depression, Anxiety, Stress Scale  Emotional Self Awareness: measured by adapting the 20-item self reflection and Insight Scale, the 10-item Ruminative Response Scale, and the 12-item Meta-Evaluation Scale. |
| Notes | Funding: This study was supported by research grants from the Telstra Foundation and The Shepherd Foundation, and was supported by the Victorian Government’s Operational Infrastructure Support Program.  Conflict of interest: N/A |

[Sun 2022](#STD-Sun-2022)

| ***Study characteristics*** | |
| --- | --- |
| Methods | Design: Randomized Controlled Trial  Objective: The aims of this randomized controlled trial were threefold. First, the primary aim was to examine the effectiveness of a mindfulnessbased mHealth intervention in reducing symptoms of anxiety and depression for young adults in quarantine compared to a rigorous active control (social support mHealth).  Secondly, we aimed to examine emotional suppression, a culturally relevant emotion regulation strategy, as a potential intervention mediator.  The third aim was to evaluate the feasibility and acceptability of the mindfulness mHealth in comparison to social support mHealth  Data collection:(including timepoints for analysis)  We recruited 114 Chinese university students during the emerging COVID-19 pandemic in March and April 2020. Recruitment took place online via WeChat-based flyers and websites targeting college students. Potentially interested participants completed a brief online screening survey to determine eligibility.  Baseline, Post-intervention (1 month), follow-up (2 months- post baseline) |
| Participants | Setting: Universities in China  Eligibility Criteria: (a) identify as Chinese; (b) currently enrolled as an undergraduate or graduate-level university student; (c) age 18 or older; (d) can read, speak, and write Mandarin Chinese; (e) self-report as currently in quarantine due to the pandemic without physically attending school; (f) have daily personal internet access; (g) have access to a smartphone or device that allows for Zoom video conferencing and WeChat, a widely used social media app in China; (h) experiencing elevated psychological distress, such that their depression or anxiety symptoms at or above the mild cutoff on the Patient Health Questionnaire-9 (PHQ-9; Chinese version; Kroenke et al., 2001; Wang et al., 2014) and the 7-item Generalized Anxiety Disorder Scale (GAD-7; Chinese version; Spitzer et al., 2006; Zeng et al., 2013).  Characteristics: Participants were 22.21 years old (SD = 2.67) on average. The majority were female (73.7%). Participants resided in 27 various provinces out of the total 34 provinces in China (79.4%), representing a wide geographical reach. The majority were undergraduate (67.5%). Most participants (59.6%) reported they perceived their family income to be at a similar level compared to their peers, whereas 28.9% reported family income as lower and 11.4% reported family income to be higher than their peers. |
| Interventions | A 4-week mindfulness-based mobile health intervention, “Mindfulness for Growth and Resilience,” was developed for this study |
| Outcomes | Anxiety: measured with Generalized Anxiety Disorder-7 (GAD-7; Chinese version)  Depression: Patient Health Questionnaire-9 (PHQ-9; Chinese version)  Mindfulness: Mindful Attention Awareness Scale (MAAS; Chinese version)  Perceived Social Support: Multidimensional Scale of Perceived Social Support (MSPSS; Chinese version) |
| Notes | Funding: This research project was supported by the Fighting COVID-19 Research Fund by Beijing Normal University awarded to Danhua Lin. The funders had no role in study design, data collection and analysis, decision to publish, or preparation of the manuscript  Conflict of interest: None of the authors have any conflict of interest to report. |

[Teng 2019](#STD-Teng-2019)

| ***Study characteristics*** | |
| --- | --- |
| Methods | Design: A randomized, controlled multi-session experiment  Objective: Assess the effect of home-delivered attentional bias modification app on symptoms in patients with generalized anxiety disorder  Data collection:(including timepoints for analysis)  The worriers (20–35 years old) were required to complete an  Internet-administered questionnaire (Penn State Worry Questionnaire,  PSWQ) for worry-severity screening. Qualified applicants (PSWQ > 60)  were then invited to a laboratory to undergo a structured interview  conducted by a research assistant. The GAD subscale of the DIS-IV was used to verify that the applicants were qualified. A total of 121 applicants expressed interest in participating in this study, and 93 of them were qualified in the interview phase.  At baseline, week 2, week 3, week 4; 1 month follow up (i.e. 1 month after the 4-week treatment ended, or 2 months after the start of the study) |
| Participants | Setting: National Chung-Cheng University, Chia-Yi, Taiwan  Eligibility Criteria:Participants needed to meet the criteria for GAD (generalized anxiety disorder)  Penn State Worry Questionnaire result > 60 ("the worriers"). The GAD subscale of the DIS-IV was used to verify that the applicants were qualified."1. To be included in this study participants were required to complete an  Internet-administered questionnaire (Penn State Worry Questionnaire, PSWQ) for worry-severity  screening and obtain PSWQ score of more than 60 points.  2. Participants were then invited to a laboratory to undergo a structured interview conducted by  a research assistant using the GAD subscale of the DIS-IV was to verify that the applicants were qualified."  Characteristics: Age:  HD-ABM 21.46(mean) 2.18 (SD)  Placebo 21.50 (mean) 1.57 (SD)  Waiting 21.50 (mean) 1.59 (SD)  Gender:  82 participants remained at the conclusion of this study: 30 in the HD-ABM group (24 women), 30 in the placebo group (23 women), and 22 in the waiting-list group (14 women).  No reporting of gender among the original 93 randomized participants.  Outcomes also measured at baseline |
| Interventions | Home-delivered attention bias modification Application: the app would display a fixation cross, after which a pair of stimulus words written in traditional Chinese characters (i.e., a threatening word and a natural word) were presented on the left and right sides of the now-missing fixation.. A target probe (displayed with an E) then replaced one of the word pairs after it disappeared. The probe was set to replace the neutral stimulus word. The participants were instructed to tap the location of the target probe (E) on the screen with their thumbs |
| Outcomes | Depressive Symptoms: measured with Beck Depression Inventory  Anxiety: measured with Beck Anxiety Inventory and Spielberger State-Trait Anxiety Inventory  Worry: measured with.Penn State Worry Questionnaire |
| Notes | Funding: This study was supported by a grant from:  (1) Ditmanson Medical Foundation Chia-Yi Christian Hospital  Research Program (R105-003)  (2) The Ministry of Science and Technology (Taiwan) (Most: 104-  2410-H-194-036)  Conflict of interest: All authors declare that they have no potential conflict of interest  pertaining to this submission to Journal of Affective Disorders |

[Thabrew 2022](#STD-Thabrew-2022)

| ***Study characteristics*** | |
| --- | --- |
| Methods | Design: randomised controlled trial of Whitu against waitlist control,  Objective: To evaluate the efficacy and acceptability of ‘Whitu: seven ways in seven days’, a well-being application (app) for young people.  Data collection:(including timepoints for analysis)  To optimise recruitment of New Zealand Māori and Pacific young people, the study was initially promoted to these groups via social media, and later opened up to individuals of any ethnicity. Participants (1) read study information, (2) completed informed consent procedures and baseline questionnaires and (3) were randomised to either the intervention group (Whitu app) or waitlist control group via REDCap, a secure web application designed to capture data for clinical research and projects that includes a randomisation module.  Baseline, 4 weeks, 3 months |
| Participants | Setting: New Zealand residents aged between 16 and 30  Eligibility Criteria: New Zealand residents aged between 16 and 30 years who had reliable access to Wi-Fi, owned either an iPhone or Android mobile phone, were considered ‘healthy volunteers’ and not currently receiving mental health treatment, and could read and understand enough English to use the app via an online social media advertising campaign were recruited for the study  Characteristics: Participants ranged between 16 and 30 years, with a mean age of 23.8 years (SD 3.8). The majority of participants were female (n=79; 87.8%) and were students (n=59; 69.6%)  total:  New-Zealand European: 27.8%  Maori: 43.3%  Pacific: 12.2%  Asian: 10%  Other: 6.7% |
| Interventions | Whitu: seven ways in seven days is a free mobile application (app) that is currently available to New Zealand users. It contains seven positive psychology, CBT and psychoeducation-based modules that can be completedwithin a week. |
| Outcomes | Emotional well-being was measured using the 5-item  WHO Well-Being Index (WHO-5)  Mental well-being was measured by the 7-item Short Warwick-Edinburgh  Mental Well-Being Scale.  Depression was measured by  the 20-item Center for Epidemiological Studies Depres-  sion Scale (CES-D).  Anxiety was measured  by the Generalised Anxiety Disorder 7-item Scale.36  Self-compassion was measured by the Self-Compassion Scale-Short Form.  Stress was measured by the  10-item Perceived Stress Scale (PSS-10).  Sleep quality was measured by the single-item Sleep Quality Scale (SQS). |
| Notes | Funding: This study was generously funded by the Starship Foundation (SF 1562) and Auckland Medical Research Foundation (grant no 1720008), New Zealand. Funders did not have any direct involvement in the design or conduct of the study, data analysis or preparation of results. AS and HT came up with the concept for developing the Whitu well-being app. The IP for the app is owned by the University of Auckland and is not-for-profit.  Conflict of interest: None declared |

[Thompson 2020](#STD-Thompson-2020)

| ***Study characteristics*** | |
| --- | --- |
| Methods | Design: A randomized controlled pilot trial  Objective: To determine whether the targeted population would be engaged by OnTrack and use it on a daily basis and if such use would result in decreased alcohol and marijuana use and sexual risk behaviors  Data collection:(including timepoints for analysis)  "A sample of eligible homeless young adults was provided a complete oral and written description of the study and invited to participate  No further sampling or recruitment strategies described"  All participants were assessed at baseline and 2 weeks, 4 weeks, and 6 weeks after baseline (outcomes of alcohol use, marijuana use, and sexual risk behaviors were assessed with the TLFB at Sessions 1–3 and  post assessment) |
| Participants | Setting: inner-city crisis shelter for homeless young adults (aged 18 –21 years)  Urban, northeastern crisis shelter  Eligibility Criteria: Young adults from an urban, northeastern crisis shelter were  eligible for the study if they were;  -Homeless  -Were 18 –21 years old  -Engaged in unprotected vaginal, anal, or oral sex one or more times  per week in the past month  -Binge drank (four or more drinks on one occasion; National Institute  on Alcohol Abuse and Alcohol ism, 2005) in the past month  -Used marijuana 4 or more days per week in the past month.  Characteristics: The final sample had an average age of 19.2 (SD 0.84; range 18 –21) years, 75% were male, 51.7% were Hispanic, 66.7% were Black, 10.0% were White, and 23.3% were of other race/ethnicity. |
| Interventions | On Track Application: the app allows for self-monitoring of substance use and sexual risk behaviors, in addition to Brief Motivational Interviewing |
| Outcomes | Number of Alcohol Drinks: measured by self-report  Use of Marjuana: measured by self-report  Unprotected Sex: measured by self-report  Alcohol or Drug Use before sex: measured by self-report |
| Notes | Funding: . This research was supported in part by a grant from the  National Institutes of Health (K23DA032323) to Ronald G. Thompson Jr  Conflict of interest: N/A |

[Torok 2022](#STD-Torok-2022)

| ***Study characteristics*** | |
| --- | --- |
| Methods | Design: 2-arm parallel, double-blind, randomized controlled trial  Objective: The primary objective of this study was to investigate the efficacy of the Lifebuoy smartphone application in reducing the severity of suicidal thoughts when compared with an attention-matched smartphone application (LifeBuoy-C). A secondary objective was to examine these effects for broader mental health outcomes of depression, anxiety, distress, and well-being.  Data collection:(including timepoints for analysis) |
| Participants | Setting: Australia  Eligibility Criteria: Eligible participants were between 18 and 25 years of age, in the community (nonclinical sample), residing in Australia at the time of registration, and who responded in the positive to the question “have you experienced suicidal thoughts in the past 12 months?” Eligible individuals also had to own a smartphone (versions Android 5 and iOS 9 or higher) and be fluent in English  Characteristics: The participant sample was primarily female (n = 384, 84.4%) with a mean age of 21.5 years (SD: 2.18). Nearly one-third had completed a tertiary qualification (n = 135, 29.7%). Mental health issues were prevalent, with 88.6% (n = 403) having ever received a mental health diagnosis and 88.1% (n = 401) have ever received mental health treatment. |
| Interventions | 7-module, self-guided DBT smart-phone application (“LifeBuoy”) designed to improve emotional regulation and increase distress tolerance skills. |
| Outcomes | SIDAS is a validated, 5 item, 11-point scale measure (Cronbach’s α = 0.62), which assesses the frequency of ideation, controllability, severity, and impact.  Recent depression symptoms (in past 2 weeks) were measured by the Patient Health Questionnaire-9 (PHQ-9).  Anxiety symptoms were measured using the Generalized Anxiety Disorder-7 (GAD-7)  Well-being was measured using the 7-item Short Warwick–Edinburgh Mental Well-Being Scale  Psychological distress was assessed using the Distress Questionnaire-5 (DQ5) |
| Notes | Funding: This trial of the Lifebuoy smartphone application was funded by the Australian Rotary Health Mental Health of Young Australians Research Grant Scheme (RG192840) and a National Health and Medical Research Council Early Career Fellowship, which was awarded to Michelle Torok (RG1138710). The development the LifeBuoy smartphone application was funded by a philanthropic grant awarded by the S & C Roth Family Foundation (RG20210528). The views and opinions expressed in this article are those of the authors and do not necessarily reflect those of the funders. The funders had no role in study design, data collection and analysis, decision to publish, or preparation of the manuscript.  Conflict of interest: The authors have declared that no competing interests exist. |

[Visser 2020](#STD-Visser-2020)

| ***Study characteristics*** | |
| --- | --- |
| Methods | Design: RCT  Objective: to test the effect of the training  on the emotional valence and strength of spontaneous autobiographical memories—our Manipulation check - and depressive symptoms.  We also explored whether the modification of memory bias would transfer further to self-referential but non-autobiographical explicit memory bias using the self-referential encoding task  Data collection:(including timepoints for analysis)  Participants were acquired via the Participation System (SONA) of the Radboud University  No further recruitment or sampling procedures are described. The authors declared that they did not calculate a sample size.  Day 1 and Day 5 (Prior to and after the MBM training) |
| Participants | Setting: Radboud University  in Nijmegen Social Sciences faculty, The Netherlands  Eligibility Criteria:Participants had to be fluent in Dutch and were selected based on age (18–70)  Characteristics: Reported in Table 2, page 66  Mean age total group: 22.8 +/- 6.4  Total group: 76% female; 24% male  The mean BDI-II score was 7.7±7.7 (range 0–49). At baseline, 13.7% (n =21) of all participants scored≥14 on the BDI-II indicating a mild depression or higher, there was no significant difference between groups χ2 (2, n=153)=2.083, p=0.353. The mean MRSI was 23.3±6.6 (range 8–39), the total mean RRS score was 40.1±11.0 (range 22–75) and the mean PANAS negative affect score was 13.84±4.3 (range 10–29).  PROGRESS+ Characteristics  - Ethnicity (%European): 96% positive group, 94% negative group, 100% control  - Native language (% Dutch): 98% positive group, 98% negative group, 98% control |
| Interventions | Memory bias modification (MBM) Application: the app was designed to to habituate the process of autobiographical information retrieval with either a positive or negative emotional valence, depending on participant’s group.  Positive Group was prompted to think of the most pleasant or positive event since the previous prompt. How was the event experienced? Describe this event in three words  Negative Group was prompted to think of the most unpleasant or negative event since the previous prompt. Describe this event in three words |
| Outcomes | Depressive Symptoms: measured with The Beck Depression Inventory |
| Notes | Funding: N/A  Conflict of interest: Damian A. Visser, Indira Tendolkar, Aart H.  Schene, Livia van de Kraats, Henricus G. Ruhe, Janna N. Vrijsen declares that he has no conflict of interest |

[Yang 2022](#STD-Yang-2022)

| ***Study characteristics*** | |
| --- | --- |
| Methods |  |
| Participants |  |
| Interventions | The participants in the intervention group were given the HARU ASD3 program, an app-based CBT program for reducing anxiety in persons with ASD |
| Outcomes | Anxiety: State-Trait Anxiety Inventory (STAI)  Dysfunctional automatic thoughts: Automatic Thought Questionnaire-Negative (ATQ-N)  Emotional State: Positive and Negative Affect Schedule (PANAS)  Aberrant behaviours: Aberrant Behavior Checklist (ABC)  Anxiety-related behaviours: Direct behavior observation |
| Notes |  |

Appendices

Appendix 1. Search Strategies (all databases)

| Medline | Embase | PsycINFO | CENTRAL | CINAHL |
| --- | --- | --- | --- | --- |
| 1. (teen* or youth* or adolescen* or juvenile* or (young adj2 (adult* or person* or individual* or people* or population* or man or men or wom#n)) or youngster* or highschool* or college* or ((secondary or high* or univ*) adj2 (school* or education or student))).ti,ab,kf. or adolescent/ or young adult/ | (teen* OR youth* OR adolescen* OR juvenile* OR (young ADJ2 (adult* OR person* OR individual* OR people* OR population* OR man OR men OR wom#n)) OR youngster* OR highschool* OR college* OR ((secondary OR high*) ADJ2 (school* OR education))).ti,ab. OR exp adolescent/ OR exp adolescence/ OR young adult/ | (teen* OR youth* OR adolescen* OR juvenile* OR (young ADJ2 (adult* OR person* OR individual* OR people* OR population* OR man OR men OR wom#n)) OR youngster* OR highschool* OR college* OR ((secondary OR high*) ADJ2 (school* OR education))).tw or exp early adolescence/ | (teen* OR youth* OR adolescen* OR juvenile* OR (young ADJ2 (adult* OR person* OR individual* OR people* OR population* OR man OR men OR wom#n)) OR youngster* OR highschool* OR college* OR ((secondary OR high*) ADJ2 (school* OR education))).tw or adolescent/ or young adult/ | (TI “teen*” or “youth” or “adolescen*” or “juvenile” or (“young” N2 (“adult*” or “person*” or “individual*” or “people” or “population*” or “man” or “woman”)) or “youngster” or ((“secondary” or “high”) N2 (“school*” or “education”))) or (AB “teen*” or “youth” or “adolescen*” or “juvenile” or (“young” N2 (“adult*” or “person*” or “individual*” or “people” or “population*” or “man” or “woman”)) or “youngster” or ((“secondary” or “high”) N2 (“school*” or “education”))) or (MH “Adolescence+”) or (MH “Young Adult”) |
| 2. Mental Health/ or Depression/ or Anxiety/ or exp Mental Disorders/ or exp suicide/ or suicidal ideation/ or suicide, attempted/ or exp Sleep Wake Disorders/ or exp Substance-Related Disorders/ | Mental Health/ or Depression/ or Anxiety/ or exp Mental disease/ or exp suicide/ or suicide attempt/ or exp Sleep disorder/ or exp Substance abuse | Exp mental health/ or exp “Depression (Emotion)”/ or exp anxiety/ or exp/ anxiety disorders/ or exp mental disorders/ or exp suicide/ or Attempted suicide/ or Suicidal ideation/ or Suicide Prevention/ or exp Self-injurious behaviour/ or exp Sleep Wake Disorders/ or exp Substance Related and Addictive Disorders/ | Mental health/ or exp Mental disorders/ or Depression/ or Anxiety/ or Anxiety disorders/ or exp Suicide/ or exp Self-Injurious Behaviour/ or exp Sleep Disorders/ or exp Substance-Related Disorders | (MH “Mental Health”) or (MH “Research, Mental Health”) or (MH “Mental Disorders+”) or (MH “Depression”) or (MH “Anxiety Disorders+”) or (MH “Anxiety”) or (MH “Suicide+”) or (MH “Suicidal Ideation”) or (MH “Self-Injurious Behaviour”) or (MH “Sleep Disorders+”) or (MH “Substance Use Disorders+”) |
| 3. (mental health* or wellbeing or well-being or anxiet* or anxious or depress* or ptsd or posttrauma* or post-trauma* or suicid* or bipolar* or psycho* or sleep disorder* or ahdh).ti,ab,kf. | (mental health* or wellbeing or well-being or anxiet* or anxious or depress* or ptsd or posttrauma* or post-trauma* or suicid* or bipolar* or psycho* or sleep disorder* or ahdh).tw,kw | (mental health* or wellbeing or well-being or anxiet* or anxious or depress* or ptsd or posttrauma* or post-trauma* or suicid* or bipolar* or psycho* or sleep disorder* or ahdh).tw | (mental health* or wellbeing or well-being or anxiet* or anxious or depress* or ptsd or posttrauma* or post-trauma* or suicid* or bipolar* or psycho* or sleep disorder* or ahdh).tw | (TI “mental health*” or “wellbeing” or “well-being” or “anxiet*” or “anxious” or “depress*” or “ptsd” or “posttrauma*” or “post-trauma*” or “suicide*” or “bipolar*” or “psycho*” or “sleep disorder*” or “adhd”) or (AB “mental health*” or “wellbeing” or “well-being” or “anxiet*” or “anxious” or “depress*” or “ptsd” or “posttrauma*” or “post-trauma*” or “suicide*” or “bipolar*” or “psycho*” or “sleep disorder*” or “adhd”) |
| 4. ((attention or hyperactiv*) adj3 (deficit or disorder)).ti,ab,kf. | ((attention or hyperactiv*) adj3 (deficit or disorder)).tw,kw | ((attention or hyperactiv*) adj3 (deficit or disorder)).tw | ((attention or hyperactiv*) adj3 (deficit or disorder)).tw | (TI (“attention” or “hyperactive*) N3 (“deficit” or “disorder”)) or (AB (“attention” or “hyperactive*) N3 (“deficit” or “disorder”)) |
| 5. ((substance* or opioid* or marijuana or cannabis or cannabinoid* or alcohol) adj3 (abus* or misus* or disorder*)).ti,ab,kf. | ((substance* or opioid* or marijuana or cannabis or cannabinoid* or alcohol) adj3 (abus* or misus* or disorder*)).tw,kw | ((substance* or opioid* or marijuana or cannabis or cannabinoid* or alcohol) adj3 (abus* or misus* or disorder*)).tw | ((substance* or opioid* or marijuana or cannabis or cannabinoid* or alcohol) adj3 (abus* or misus* or disorder*)).tw | (TI (“substance*” or “opioid*” or “marijuana” or “cannabis” or “cannabinoid*” or “alcohol”) N3 (“abus* or “misus* or “disorder*”)) or (AB ((“substance*” or “opioid*” or “marijuana” or “cannabis” or “cannabinoid*” or “alcohol”) N3 (“abus* or “misus* or “disorder*”)) |
| 6. ((eating or feed* or food*) adj2 disorder).ti,ab,kf. | ((eating or feed* or food*) adj2 disorder).tw,kw | ((eating or feed* or food*) adj2 disorder).tw | ((eating or feed* or food*) adj2 disorder).tw | (TI (“eating” or “feed*” or “food*”) N2 “disorder”) or (AB (“eating” or “feed*” or “food*”) N2 “disorder”) |
| 7. (self adj2 (harm* or injur* or mutilat*)).ti,ab,kf. | (self adj2 (harm* or injur* or mutilat*)).tw,kw | (self adj2 (harm* or injur* or mutilat*)).tw | (self adj2 (harm* or injur* or mutilat*)).tw | (TI “self” N2 (“harm*” or “injur*” or “mutilate*”)) or (AB “self” N2 (“harm*” or “injur*” or “mutilate*”)) |
| 8. 2 or 3 or 4 or 5 or 6 or 7 | 2 or 3 or 4 or 5 or 6 or 7 | 2 or 3 or 4 or 5 or 6 or 7 | 2 or 3 or 4 or 5 or 6 or 7 | S2 or S3 or S4 or S5 or S6 or S7 |
| 9. Mobile applications/ or Cell Phone/ | Exp mobile application/ or exp mobile phone/ | Exp Mobile Health/ or exp Mobile Applications/ or exp Mobile Phones | Mobile Applications/ or Cellular Phone/ | (MH “Mobile Applications”) or (MH “Cellular Phone+”) |
| 10. (Mhealth* or m-health* or app or apps or mobile or portable or (cell adj2 phone) or smartphone).ti,ab,kf. | (Mhealth* or m-health* or app or apps or mobile or portable or (cell adj2 phone) or smartphone).tw,kw | (Mhealth* or m-health* or app or apps or mobile or portable or (cell adj2 phone) or smartphone).tw | (Mhealth* or m-health* or app or apps or mobile or portable or (cell adj2 phone) or smartphone).tw | (TI “mhealth*” or “m-health” or “app” or “apps” or “mobile” or “portable or (“cell N2 “phone”) or “smartphone”) or (AB “mhealth*” or “m-health” or “app” or “apps” or “mobile” or “portable or (“cell N2 “phone”) or “smartphone”) |
| 11. 9 or 10 | 9 or 10 | 9 or 10 | 9 or 10 | S9 or S10 |
| 12. Randomized controlled trials as Topic/ or Randomized controlled trial/ or Random allocation/ or Double blind method/ or Single blind method/ or Clinical trial/ or exp Clinical Trials as Topic/ | Clinical trial/ or Randomized controlled trial/ or Randomization/ or Single blind procedure/ or Double blind procedure/ or Crossover procedure/ or Placebo/ or Prospective study/ | Clinical trials/ or exp Randomized Controlled trials/ or Placebo/ |  | ( (MH "Random Assignment") or (MH "Random Sample+") or (MH "Crossover Design") or (MH "Clinical Trials+") or (MH "Comparative Studies") or (MH "Control (Research)+") or (MH "Control Group") or (MH "Factorial Design") or (MH "Quasi-Experimental Studies+") or (MH "Placebos") or (MH "Meta Analysis") or (MH "Sample Size") or (MH "Research, Nursing") or (MH "Research Question") or (MH "Research Methodology+") or (MH "Evaluation Research+") or (MH "Concurrent Prospective Studies") or (MH "Prospective Studies") or (MH "Nursing Practice, Research-Based") or (MH "Solomon Four-Group Design") or (MH "One-Shot Case Study") or (MH "Pretest-Posttest Design+") or (MH "Static Group Comparison") or (MH "Study Design") or (MH "Clinical Research+") ) |
| 13. ((clinic$ adj trial$1) or ((singl$ or doubl$ or tripl$) adj (blind$3 or mask$3)) or randomly allocated or placebo* or (allocated adj2 random) or randomized controlled trial or controlled clinical trial).tw. | (Randomi?ed controlled trial$ or rct or Random allocation or Randomly allocated or Allocated randomly or Single blind$ or Double blind$ or Placebo$ or (allocated adj2 random) or ((treble or triple) adj (blind$))).tw | (Randomi?ed controlled trial$ or rct or Random allocation or Randomly allocated or Allocated randomly or Single blind$ or Double blind$ or Placebo$ or (allocated adj2 random) or ((treble or triple) adj (blind$))).tw |  | ( TX “clinical nursing research” or “random*” or “cross?over” or “placebo*” or “control*” or “factorial” or “sham*” or “blind*” or “mask*” or “trial*” ) |
| 14. 12 or 13 | 12 or 13 | 12 or 13 |  | S12 or S13 |
| 15. 1 and 8 and 11 and 14 | 1 and 8 and 11 and 14 | 1 and 8 and 11 and 14 | 1 and 8 and 11 | S1 and S8 and s11 and S14 |
| 16. Animals/ not Humans/ | Animal/ not Human/ | Animal/ not Human/ | Animal/ not Human/ |  |
| 17. 15 not 16 | 15 not 16 | 15 not 16 | 12 not 13 |  |
| 18. limit 17 to yr="2008 -Current" | limit 17 to yr="2008 -Current" | limit 17 to yr="2008 -Current" | Limit 14 to yr="2008 -Current" | S15 (Limiters – Published Date:20080101) |

Appendix 2. Ongoing studies

| Covidence # | Authors or trial registry | Title (Up to date: 08 July; 97 exclusions) | Status |  |
| --- | --- | --- | --- | --- |
| #1153 | Lunkenheimer | Effectiveness and cost-effectiveness of guided Internet- and mobile-based CBT for adolescents and young adults with chronic somatic conditions and comorbid depression and anxiety symptoms (youthCOACHCD): study protocol for a multicentre randomized controlled trial | Ongoing |  |
| #1286 | Nct, | The SEAMLESS Study: smartphone App-based Mindfulness for Cancer Survivors | Ongoing |  |
| #1297 | Nct, | SME(Sharing, Mind & Enjoyment) App for Adolescents | Complete | No published results available |
| #1303 | Nct, | The Effectiveness of the Mobile-based Youth COMPASS Program to Promote Adolescent Well-being and Life-control | Published | [Puolakanaho, A., Lappalainen, R., Lappalainen, P., Muotka, J. S., Hirvonen, R., Eklund, K. M., Ahonen, T., & Kiuru, N. (2019). Reducing Stress and Enhancing Academic Buoyancy among Adolescents Using a Brief Web-based Program Based on Acceptance and Commitment Therapy: A Randomized Controlled Trial. Journal of youth and adolescence, 48(2), 287–305. https://doi.org/10.1007/s10964-018-0973-8](https://doi.org/10.1007/s10964-018-0973-8) |
| #1312 | Nct, | The Efficacy of a Mobile Application for Treating Depression and Anxiety Symptoms | Unclear | No published results available |
| #1316 | Nct, | The Mobile PTSD Coach App in Acute Injury Survivors | Published | Pacella‐LaBarbara, M. L., Suffoletto, B. P., Kuhn, E., Germain, A., Jaramillo, S., Repine, M., & Callaway, C. W. (2020). A Pilot Randomized Controlled Trial of the PTSD Coach App Following Motor Vehicle Crash‐Related Injury. Academic Emergency Medicine. |
| #1368 | Nct, | Youth Mayo Clinic Anxiety Coach Pilot Study | Ongoing |  |
| #1371 | Nct, | CopeSmart: using Mobile Technology to Promote Positive Mental Health in Young People | Published | Kenny, R., Fitzgerald, A., Segurado, R., & Dooley, B. (2019). Is there an app for that? A cluster randomised controlled trial of a mobile app–based mental health intervention. Health informatics journal, 1460458219884195. |
| #1391 | Nct, | A Real-time, Contextual Intervention Using Mobile Technology to Reduce Marijuana Use in Youth | Published | Shrier, L. A., Burke, P. J., Kells, M., Scherer, E. A., Sarda, V., Jonestrask, C., ... & Harris, S. K. (2018). Pilot randomized trial of MOMENT, a motivational counseling-plus-ecological momentary intervention to reduce marijuana use in youth. Mhealth, 4. |
| #1415 | Nct, | A Mobile Phone Self-Monitoring Tool to Increase Emotional Self-Awareness and Reduce Depression in Young People | Published | Reid SC, Kauer SD, Hearps SJ, Crooke AH, Khor AS, Sanci LA, Patton GC. A mobile phone application for the assessment and management of youth mental health problems in primary care: health service outcomes from a randomised controlled trial of mobiletype. BMC Fam Pract. 2013 Jun 19;14:84. doi: 10.1186/1471-2296-14-84. Kauer SD, Reid SC, Crooke AH, Khor A, Hearps SJ, Jorm AF, Sanci L, Patton G. Self-monitoring using mobile phones in the early stages of adolescent depression: randomized controlled trial. J Med Internet Res. 2012 Jun 25;14(3):e67. doi: 10.2196/jmir.1858. Reid SC, Kauer SD, Hearps SJ, Crooke AH, Khor AS, Sanci LA, Patton GC. A mobile phone application for the assessment and management of youth mental health problems in primary care: a randomised controlled trial. BMC Fam Pract. 2011 Nov 29;12:131. doi: 10.1186/1471-2296-12-131. |
| #1681 | Nct, | Using Information and Communication Technologies to Prevent Suicide in Chile | Unclear | No published results available |
| #1683 | Nct, | The BEACON Study: smartphone-Assisted Problem-Solving Therapy in Men Presenting to the ED With Self-Harm (Protocol A) | Unknown | [Hatcher S, Whittaker R, Patton M, Miles WS, Ralph N, Kercher K, Sharon C. Web-based Therapy Plus Support by a Coach in Depressed Patients Referred to Secondary Mental Health Care: Randomized Controlled Trial JMIR Mental Health, 5(1):E5. Available at: http://mental.jmir.org/2018/1/e5/](http://mental.jmir.org/2018/1/e5/) |
| #1940 | Isrctn, | Can adolescent emotion regulation be improved with an app-based training? | Ongoing |  |
| #1942 | Isrctn, | Understanding engagement with an app targeting harmful drinking: development and evaluation of the BRANCH smartphone app | Complete | No published results available |
| #1951 | Isrctn, | A mobile-based serious game for young adults with disordered eating | Complete | No published results available |
| #1953 | Isrctn, | Developing an app to help young people self-manage when feeling overwhelmed during lessons | Complete | Edridge C, Deighton J, Wolpert M, Edbrooke-Childs J. The Implementation of an mHealth Intervention (ReZone) for the Self-Management of Overwhelming Feelings Among Young People. JMIR Form Res. 2019;3(2):e11958. Published 2019 May 2. doi:10.2196/11958 |
| #1968 | Irct20190520043645N, | Evaluation the impact of psycho education on anxiety, depression and mania in bipolar disorder patients based on mobile application | Ongoing |  |
| #2190 | Golchert, Johannes; | HELP@APP: development and evaluation of a self-help app for traumatized Syrian refugees in Germany - a study protocol of a randomized controlled trial | Ongoing |  |
| #2523 | Actrn, | Evaluation of the effectiveness of a youth mobile help-seeking ToolKit | Ongoing |  |
| #2525 | Actrn, | App-based brain training for young people with depression | Ongoing |  |
| #2526 | Actrn, | Peer Tree: a smartphone application for young people (aged 16-25) with psychosis and other mental health conditions | Ongoing |  |
| #2527 | Actrn, | The Future Proofing Study: a School-Based Depression Prevention Trial | Ongoing |  |
| #2528 | Actrn, | A trial of a smartphone-based youth suicide prevention application | Ongoing |  |
| #2529 | Actrn, | The Dream On Study: a randomised controlled trial of efficacy and acceptability of a smart phone application in improving sleep in young people | Ongoing |  |
| #2532 | Actrn, | WeClick: evaluating a mobile app to improve young people's relationships | Ongoing |  |
| #2533 | Actrn, | Evaluation of an app to support emotional wellbeing of adolescents experiencing depression and/or anxiety | Ongoing |  |
| #2537 | Actrn, | Evaluating a smartphone app focussed on helping people reduce their drinking | Ongoing |  |
| #2543 | Actrn, | The effect of mobile mindfulness meditation on distress in two cohorts: incoming university students and university staff | Complete | No published results available |
| #2545 | Actrn, | MindExpressTM: trial of a depression prevention program for young people vulnerable to depressive disorders | Ongoing |  |
| #2548 | Actrn, | Test of a mobile app to aid mindfulness and improve wellbeing in young people | Complete | No published results available |
| #2552 | Actrn, | The Horyzons trial: moderated Online Social Therapy for Maintenance of Treatment Effects from Specialised First Episode Psychosis Services | Complete | No published results available |
| #2553 | Actrn, | Using an app for suicide prevention amongst young Indigenous people: a randomised controlled trial | Ongoing |  |
| #892 | Nct, | Tracking Our Lives Study | Complete | No published results available |
| #899 | Nct, | BRITEPath, Component 3 of iCHART (Integrated Care to Help At-Risk Teens) | Ongoing |  |
| #900 | Nct, | Wiring Adolescents With Social Anxiety Via Behavioral Interventions | Ongoing |  |
| #908 | Nct, | Technology Enhanced Family Treatment | Ongoing |  |
| #950 | Nct, | The Norwegian "iCanCope With Pain" App | Complete | Not published yet |
| #954 | Nct, | The Pain Squad+ Smartphone App To Support Real-Time Pain Management for Adolescents With Cancer | Ongoing |  |
| #36 | Wiljer 2016 | Enhancing Self-Efficacy for Help-Seeking Among Transition-Aged Youth in Postsecondary Settings With Mental Health and/or Substance Use Concerns, Using Crowd-Sourced Online and Mobile Technologies: The Thought Spot Protocol | Complete | Sanci L, Kauer S, Thuraisingam S, Davidson S, Duncan A, Chondros P, Mihalopoulos C, Buhagiar K. Effectiveness of a Mental Health Service Navigation Website (Link) for Young Adults: Randomized Controlled Trial. JMIR Mental Health 2019;6(10):e13189 |
| #60 | Werner-Seidler 2020 | A trial protocol for the effectiveness of digital interventions for preventing depression in adolescents: The Future Proofing Study | Ongoing |  |
| #485 | Shand 2013 | The effectiveness of a suicide prevention app for indigenous Australian youths: study protocol for a randomized controlled trial | Complete | Tighe J, Shand F, Ridani R, et al. Ibobbly mobile health intervention for suicide prevention in Australian Indigenous youth: a pilot randomised controlled trial BMJ Open 2017;7:e013518. doi: 10.1136/bmjopen-2016-013518 |
| #487 | Shand 2019 | The iBobbly Aboriginal and Torres Strait Islander app project: Study protocol for a randomised controlled trial | Complete | No results published |
| #488 | Shand 2014 | A randomised controlled trial to evaluate the effect of a self-managed acceptance and commitment therapy based app for Indigenous youths with suicidal ideation compared against wait list control | Complete | Tighe J, Shand F, Ridani R, et al. Ibobbly mobile health intervention for suicide prevention in Australian Indigenous youth: a pilot randomised controlled trial BMJ Open 2017;7:e013518. doi: 10.1136/bmjopen-2016-013518 |
| #511 | Schweizer 2019 | Protocol for an app-based affective control training for adolescents: proof-of-principle double-blind randomized controlled trial | Ongoing |  |
| #577 | Ruegg 2018 | An internet-based intervention for people with psychosis (EviBaS): study protocol for a randomized controlled trial | Complete | No published results found |
| #618 | Ridani 2013 | A controlled pilot trial to evaluate the effect on suicidal ideation and mental health of a self-managed acceptance and commitment therapy based app delivered on tablets for youths with suicidal ideation compared against wait list control | Complete | No published results found |
| #826 | Ntr, | Een nieuwe smartphone gestuurde zelfhulp interventie om zelfbeeld te verhogen in jongeren die tijdens de jeugd ingrijpende gebeurtenissen hebben meegemaakt | Ongoing |  |
| #828 | Ntr, | Traumatized Youths in Residential Care: exploring the Dysregulation of Biological Stress Systems and Testing a Gameful Relaxation Intervention to Normalize These Stress Systems | Ongoing |  |
| #829 | Ntr, | A gamfied "goal-setting" app for adolescents in mental healthcare | Ongoing |  |
| #834 | Noone 2016 | A protocol for a randomised active-controlled trial to evaluate the effects of an online mindfulness intervention on executive control, critical thinking and key thinking dispositions in a university student sample | Complete | Noone C, Hogan MJ. A randomised active-controlled trial to examine the effects of an online mindfulness intervention on executive control, critical thinking and key thinking dispositions in a university student sample. BMC Psychol. 2018;6(1):13. Published 2018 Apr 5. doi:10.1186/s40359-018-0226-3 |
| #838 | Nl, | StayFine RCT: app-based anxiety and depression relapse prevention in adolescents | Ongoing |  |
| #865 | Nct, | Shadows Edge Mobile Developing Resilience in Adolescent and Young Adult Cancer Survivors | Ongoing |  |
| #871 | Nct, | Establishing Efficacy of an Inpatient Intervention and Phone App to Reduce Suicidal Risk | Ongoing |  |
| #873 | Nct, | NettOpp: the Development and Evaluation of an App-based Selective Intervention for Adolescents Exposed to Cyberbullying | Ongoing |  |
| #875 | Nct, | Harnessing Mobile Technology to Reduce Mental Health Disorders in College Populations | Ongoing |  |
| #876 | Nct, | App-based Mental Health Promotion in Young European Adults | Ongoing |  |
| #881 | Nct, | A Mobile-App Training to Reduce Body Image Disorder Symptoms and Associated Features in Female University Students | Ongoing |  |
| #882 | Nct, | Depression, Trauma, and Health: efficacy of an mHealth App for Symptom Self-Management in College Students | Ongoing |  |
| #883 | Nct, | A Mobile Health and Social Media Physical Activity Intervention Among Adolescent and Young Adult Childhood Cancer Survivors | Ongoing |  |
| #884 | Nct, | Examining the Efficacy of Acceptance and Commitment Therapy (ACT) Microinterventions for Distressed First-Generation College Students | Ongoing |  |
| #890 | Nct, | Study for the Use Smartphone Application to Prevent Suicidal Relapse Among 15-35 Years-old With Previous Suicide Attempted | Ongoing |  |
| #902 | Nct, | Developing and Testing Motherly App: a Smartphone Application to Promote Mothers' Mental Health | Ongoing |  |
| #905 | Nct, | Peer i-Coaching for Activated Self-Management Optimization in Adolescents and Young Adults With Chronic Conditions | Ongoing |  |
| #911 | Nct, | Project IntERact Study | Ongoing |  |
| #912 | Nct, | Examining the Feasibility of the Ask RoSE Mobile Mental Health Application | Ongoing |  |
| #918 | Nct, | Alcohol & Mobile Phone Study to Reduce High-risk Alcohol Use and Consequences | Ongoing |  |
| #923 | Nct, | Evaluation of the Treatment Approach ROBIN | Ongoing |  |
| #926 | Nct, | Effects of Mindfulness Training on the Emotional Experience and (Non-) Acceptance of Emotions in Adolescents | Ongoing |  |
| #943 | Nct, | Mobile Intervention for Young Opioid Users | Unknown | No published results available |
| #949 | Nct, | Evaluating a Mobile App for Students Seeking Care for Depression and Anxiety at Harvard University Health Services | Ongoing |  |
| #951 | Nct, | Automated Assessment Using Facial Coding | Ongoing |  |
| #989 | Merry 2018 | Therapy Apps: what They Can and Can't Do | Exclude | This is a presentation on guidelines to use therapy apps |
| #1273 | Nct, | Smartphone-enabled Health Coaching Intervention for Youth Diagnosed With Major Depressive Disorders | Ongoing |  |
| #1294 | Nct, | Impact of Mobile App on Purpose and Well-Being Among College Students | Complete | No published results available |
| #1295 | Nct, | Prevention of Comorbid Depression and Obesity in Attention-deficit/ Hyperactivity Disorder | Unknown | No published results available |
| #1301 | Nct, | Smartphone Addiction Recovery Coach for Adolescents (SARC-A) Experiment | Ongoing |  |
| #1306 | Nct, | Flourishing App: an Evaluation With High School Students | Complete | No published results available |
| #1308 | Nct, | Effects of Community-based Caring Contact on Post-discharge Young Adults With Self-harm | Ongoing |  |
| #1313 | Nct, | A Pilot Randomized Control Trial to Help Youth Smokers to Quit Smoking | Ongoing |  |
| #1315 | Nct, | Phone App for Grounding | Ongoing |  |
| #1321 | Nct, | Impact of a Mobile Application (Pacifica) on Stress, Anxiety, and Depression | Complete | Moberg, C., Niles, A., & Beermann, D. (2019). Guided self-help works: randomized waitlist controlled trial of Pacifica, a mobile app integrating cognitive behavioral therapy and mindfulness for stress, anxiety, and depression. Journal of medical Internet research, 21(6), e12556. |
| #1348 | Nct, | MYPLAN - Effectiveness of a Safety Plan App to Manage Crisis of Persons at Risk of Suicide | Unknown | No published results available |
| #1359 | Nct, | Development and Testing of a Smartphone Application to Reduce Substance Use and Sexual Risk Among Homeless Young Adults | Complete | Thompson RG, Aivadyan C, Stohl M, Aharonovich E, Hasin DS. Smartphone application plus brief motivational intervention reduces substance use and sexual risk behaviors among homeless young adults: Results from a randomized controlled trial [published online ahead of print, 2020 Mar 16]. Psychol Addict Behav. 2020;10.1037/adb0000570. doi:10.1037/adb0000570 |
| #1360 | Nct, | Optimizing a Smartphone Application for Individuals With Eating Disorders | Complete | Kim JP, Sadeh-Sharvit S, Darcy AM, et al. The Utility and Acceptability of a Self-Help Smartphone Application for Eating Disorder Behaviors. J Technol Behav Sci. 2018;3(3):161-164. |
| #1369 | Nct, | Mobile App of CBT for Anxiety and Cancer | Complete | Joseph Greer, Jamie M. Jacobs, Nicole Pensak, James MacDonald, Charn-Xin Fuh, Giselle Katiria Perez, Alina Ward, Anne Holt, Colleen Tallen, Alona Muzikansky, Lara N. Traeger, Frank J. Penedo, Steven Safren, William F. Pirl, and Jennifer S. Temel. Randomized trial of a cognitive-behavioral therapy mobile app for anxiety in patients with incurable cancer. Journal of Clinical Oncology 2017 35:15_suppl, 10022-10022 |
| #1374 | Nct, | Mobile Phone App for Depression and Anxiety in Young Men Who Are Attracted to Men | Withdrawn |  |
| #1377 | Nct, | Brief Intervention for Suicide Risk Reduction in High Risk Adolescents | Complete | Kennard BD, Goldstein T, Foxwell AA, et al. As Safe as Possible (ASAP): A Brief App-Supported Inpatient Intervention to Prevent Postdischarge Suicidal Behavior in Hospitalized, Suicidal Adolescents [published correction appears in Am J Psychiatry. 2019 Sep 1;176(9):764]. Am J Psychiatry. 2018;175(9):864-872. doi:10.1176/appi.ajp.2018.17101151 |
| #1385 | Nct, | An Exercise App to Reduce Young Adults' MJ Use | Complete | No published results available |
| #1409 | Nct, | Cell Phone-supported Cognitive Behavioural Therapy | Unknown | No published results available |
| #1496 | Kolar 2017 | Smartphone-Enhanced Low-Threshold Intervention for adolescents with Anorexia Nervosa (SELTIAN) waiting for outpatient psychotherapy: Study protocol of a randomised controlled trial | Complete | No published results available |
| #1686 | Nct, | Bridging the Gap - Tools for Finding Health, Mental Health and Wellness Resources for University and College Students | Complete | No published results available |
| #1721 | Drks, | Effectiveness and cost-effectiveness of guided internet-and mobile-based CBT for adolescents and young adults with chronic somatic conditions and comorbid depression and anxiety symptoms (youthCOACHcd): a multicentre randomized controlled trial with a 12-month follow-up | Ongoing |  |
| #1728 | Drks, | Smartphone-based intervention (S.M.I.L.E.R.S.) to reduce mild to moderate depressive symptoms in Arabic-speaking clients | Ongoing |  |
| #1730 | Drks, | Treatment program including a smartphone app addressing adolescents with problems in academic achievement and mental health problems in Cologne | Ongoing |  |
| #1734 | Drks, | A smartphone-enhanced low-threshold intervention for adolescents with Anorexia nervosa waiting for outpatient psychotherapy | Ongoing |  |
| #2432 | Bolinski 2018 | Effectiveness of a transdiagnostic individually tailored Internet-based and mobile-supported intervention for the indicated prevention of depression and anxiety (ICare Prevent) in Dutch college students: Study protocol for a randomised controlled trial | Complete | Weisel KK, Zarski AC, Berger T, et al. Efficacy and cost-effectiveness of guided and unguided internet- and mobile-based indicated transdiagnostic prevention of depression and anxiety (ICare Prevent): A three-armed randomized controlled trial in four European countries. Internet Interv. 2018;16:52-64. Published 2018 Apr 15. doi:10.1016/j.invent.2018.04.002 |
| #2642 | Clarke 2015 | A mobile phone and web-based intervention for improving mental well-being in young people with type 1 diabetes: design of a randomized controlled trial | Stopped |  |

| Covidence # | Authors | Title | Status | (Ongoing, published, unclear) If published, link here |
| --- | --- | --- | --- | --- |
| 5701 | ACTRN12614000686606 | Using an app for suicide prevention amongst young Indigenous people: a randomised controlled trial | Ongoing |  |
| 5707 | ACTRN12616000365460 | The use of mobile 'apps' as treatment for social anxiety in university students: a randomised controlled trial | Ongoing |  |
| 5680 | ACTRN12616000634471 | Mobile Mindfulness Meditation as an Adjunct to Treatment-as-usual: a Randomised Controlled Trial | Ongoing |  |
| 5090 | ACTRN12616000651482 | Test of a mobile app to aid mindfulness and improve wellbeing in young people | Unclear |  |
| 5690 | ACTRN12616001626459 | MindExpressTM: trial of a depression prevention program for young people vulnerable to depressive disorders | Ongoing |  |
| 5089 | ACTRN12617000300370 | The effect of mobile mindfulness meditation on distress in two cohorts: incoming university students and university staff | Unclear |  |
| 4679 | ACTRN12618001604291 | A randomised control trial of the Uprise online program for university students | Unclear |  |
| 4650 | ACTRN12618001877279 | Evaluation of an app to support emotional wellbeing of adolescents experiencing depression and/or anxiety | Ongoing |  |
| 4647 | ACTRN12618001982202 | WeClick: evaluating a mobile app to improve young people's relationships | Ongoing |  |
| 5086 | ACTRN12618002010279 | Evaluating a mindfulness mobile phone app in a University student population | Unclear |  |
| 4625 | ACTRN12619000236190 | Peer Tree: a smartphone application for young people (aged 16-25) with psychosis and other mental health conditions | Ongoing |  |
| 4614 | ACTRN12619000350123 | Peer Tree: a smartphone application for tertiary students | Ongoing |  |
| 4619 | ACTRN12619000377134 | Evaluation of the effectiveness of a youth mobile help-seeking ToolKit | Ongoing |  |
| 4621 | ACTRN12619000712101 | App-based brain training for young people with depression | Ongoing |  |
| 4627 | ACTRN12619000855123 | The Future Proofing Study: a School-Based Depression Prevention Trial | Ongoing |  |
| 5076 | ACTRN12619001302145 | I bet there's an app for that: using mental health apps to manage anxiety and depression | Ongoing |  |
| 5073 | ACTRN12619001671156 | A trial of a smartphone-based youth suicide prevention application | Ongoing |  |
| 3973 | Azam 2019 | Effects of a 12-Minute Smartphone-Based Mindful Breathing Task on Heart Rate Variability for Students With Clinically Relevant Chronic Pain, Depression, and Anxiety: Protocol for a Randomized Controlled Trial | Ongoing |  |
| 2997 | Berman 2015 | Mobile Phone Apps for University Students With Hazardous Alcohol Use: Study Protocol for Two Consecutive Randomized Controlled Trials | Published | Gajecki M, Andersson C, Rosendahl I, Sinadinovic K, Fredriksson M, Berman AH. Skills Training via Smartphone App for University Students with Excessive Alcohol Consumption: a Randomized Controlled Trial. Int J Behav Med. 2017 Oct;24(5):778-788. doi: 10.1007/s12529-016-9629-9.   Berman AH, Gajecki M, Fredriksson M, Sinadinovic K, Andersson C. Mobile Phone Apps for University Students With Hazardous Alcohol Use: Study Protocol for Two Consecutive Randomized Controlled Trials. JMIR Res Protoc. 2015 Dec 22;4(4):e139. doi: 10.2196/resprot.4894. |
| 3503 | Bertholet 2020 | Smartphone-based secondary prevention intervention for university students with unhealthy alcohol use identified by screening: study protocol of a parallel group randomized controlled trial | Ongoing |  |
| 3676 | Boucher 2021 | Effects of a Digital Mental Health Program on Perceived Stress in Adolescents Aged 13-17 Years: Protocol for a Randomized Controlled Trial. | Ongoing |  |
| 3681 | Boumparis 2021 | A Mobile Intervention to Promote Low-Risk Drinking Habits in Young Adults: Protocol for a Randomized Controlled Trial. | Ongoing |  |
| 5122 | ChiCTR1900026506 | Efficacy of a Mindfulness-Based Mobile Application for Students in Tertiary Education: a Randomised Controlled Trial | Ongoing |  |
| 5686 | DRKS00008946 | A smartphone-enhanced low-threshold intervention for adolescents with Anorexia nervosa waiting for outpatient psychotherapy | Ongoing |  |
| 5692 | DRKS00009862 | Treatment program including a smartphone app addressing adolescents with problems in academic achievement and mental health problems in Cologne | Unclear |  |
| 4729 | DRKS00020941 | Application of a self-help smartphone app for students with depressive symptoms: a randomized controlled trial | Ongoing |  |
| 4753 | DRKS00022328 | Prevention of psychotropic substance use and internet-related disorders among vocational students | Ongoing |  |
| 5311 | DRKS00023478 | iCHIMPS - an internet- and mobile-based intervention for children and adolescents of mentally ill parents: a randomized controlled pilot study | Ongoing |  |
| 3686 | Fitzsimmons-Craft 2021 | Harnessing mobile technology to reduce mental health disorders in college populations: A randomized controlled trial study protocol | Unclear |  |
| 3671 | Fucito 2021 | A Multimodal Mobile Sleep Intervention for Young Adults Engaged in Risky Drinking: Protocol for a Randomized Controlled Trial | Ongoing |  |
| 3589 | Han 2020 | A Mobile Health Intervention (LifeBuoy App) to Help Young People Manage Suicidal Thoughts: Protocol for a Mixed-Methods Randomized Controlled Trial | Ongoing |  |
| 3246 | Harrer 2019 | Internet- and App-Based Stress Intervention for Distance-Learning Students With Depressive Symptoms: Protocol of a Randomized Controlled Trial. | Published | Harrer, M., Apolinário-Hagen, J., Fritsche, L., Salewski, C., Zarski, A. C., Lehr, D., Baumeister, H., Cuijpers, P., & Ebert, D. D. (2021). Effect of an internet- and app-based stress intervention compared to online psychoeducation in university students with depressive symptoms: Results of a randomized controlled trial. Internet interventions, 24, 100374. https://doi.org/10.1016/j.invent.2021.100374 |
| 6766 | Haug 2020 | Efficacy of a smartphone-based coaching program for addiction prevention among apprentices: study protocol of a cluster-randomised controlled trial | Ongoing |  |
| 6153 | Hui 2020 | Treating depression with a smartphone-delivered self-help cognitive behavioral therapy for insomnia: Study protocol for a parallel group randomized controlled trial | Ongoing |  |
| 5681 | ISRCTN13425994 | Developing an app to help young people self-manage when feeling overwhelmed during lessons | Published | Edridge C, Deighton J, Wolpert M, Edbrooke-Childs J. The Implementation of an mHealth Intervention (ReZone) for the Self-Management of Overwhelming Feelings Among Young People. JMIR Form Res. 2019 May 2;3(2):e11958. doi: 10.2196/11958. PMID: 31045499; PMCID: PMC6521198. |
| 5682 | ISRCTN15315334 | Positive Psychology Smartphone Application | Ongoing |  |
| 5087 | ISRCTN17156687 | Study of a smartphone-delivered, therapist-supported mindfulness-based therapy program for depression in Finnish university students | Published | Raevuori A, Vahlberg T, Korhonen T, Hilgert O, Aittakumpu-Hyden R, Forman-Hoffman V. A therapist-guided smartphone app for major depression in young adults: A randomized clinical trial. Journal of Affective Disorders. 2021 May 1;286:228-38. |
| 4648 | ISRCTN17213032 | Can adolescent emotion regulation be improved with an app-based training? | Ongoing |  |
| 5678 | ISRCTN19383681 | Assessing the efficacy of an intervention to improve mind-mindedness in teenage mothers | Published | Larkin F, Oostenbroek J, Lee Y, Hayward E, Meins E. Proof of concept of a smartphone app to support delivery of an intervention to facilitate mothers' mind-mindedness. PLoS One. 2019 Aug 22;14(8):e0220948. doi: 10.1371/journal.pone.0220948. PMID: 31437173; PMCID: PMC6705768. |
| 5308 | ISRCTN46697028 | How can I influence my future? A study of using an app to strengthen mental health among young adults who are not in education or employment | Ongoing |  |
| 5694 | ISRCTN55102899 | Comparing counseling alone versus counseling supplemented with a well-being mobile phone app for university students with anxiety or depression | Published | Broglia E, Millings A, Barkham M. Counseling With Guided Use of a Mobile Well-Being App for Students Experiencing Anxiety or Depression: Clinical Outcomes of a Feasibility Trial Embedded in a Student Counseling Service. JMIR Mhealth Uhealth. 2019 Aug 15;7(8):e14318. doi: 10.2196/14318. PMID: 31418424; PMCID: PMC6714497. |
| 5735 | ISRCTN70907354 | A mobile-based serious game for young adults with disordered eating | Unclear |  |
| 4688 | ISRCTN70980706 | Understanding engagement with an app targeting harmful drinking: development and evaluation of the BRANCH smartphone app | Published | Milward J, Deluca P, Drummond C, Kimergård A. Developing Typologies of User Engagement With the BRANCH Alcohol-Harm Reduction Smartphone App: Qualitative Study. JMIR Mhealth Uhealth. 2018 Dec 13;6(12):e11692. doi: 10.2196/11692. PMID: 30545806; PMCID: PMC6315270. |
| 4704 | ISRCTN86142301 | Evaluating mobile prototypes utilization of individual meal recording | Published | Liu YC, Chen CH, Tsou YC, Lin YS, Chen HY, Yeh JY, Chiu SY. Evaluating Mobile Health Apps for Customized Dietary Recording for Young Adults and Seniors: Randomized Controlled Trial. JMIR Mhealth Uhealth. 2019 Feb 15;7(2):e10931. doi: 10.2196/10931. PMID: 30767906; PMCID: PMC6404641. |
| 5085 | ISRCTN94097629 | Assessing the effects of digital meditation on the cognitive function of adolescents with childhood trauma | Unclear |  |
| 3601 | Kroska 2020 | Optimizing an Acceptance and Commitment Therapy Microintervention Via a Mobile App With Two Cohorts: Protocol for Micro-Randomized Trials. | Published | Kroska EB, Hoel S, Victory A, Murphy SA, McInnis MG, Stowe ZN, Cochran A. Optimizing an Acceptance and Commitment Therapy Microintervention Via a Mobile App With Two Cohorts: Protocol for Micro-Randomized Trials. JMIR Res Protoc. 2020 Sep 23;9(9):e17086. doi: 10.2196/17086. |
| 3573 | Kuchler 2020 | StudiCare mindfulness-study protocol of a randomized controlled trial evaluating an internet- and mobile-based intervention for college students with no and "on demand" guidance | Unclear |  |
| 3511 | Lunkenheimer 2020 | Effectiveness and cost-effectiveness of guided Internet- and mobile-based CBT for adolescents and young adults with chronic somatic conditions and comorbid depression and anxiety symptoms (youthCOACHCD): study protocol for a multicentre randomized control | Ongoing |  |
| 3578 | Yokomitsu 2020 | Gamified Mobile Computerized Cognitive Behavioral Therapy for Japanese University Students With Depressive Symptoms: Protocol for a Randomized Controlled Trial | Unclear |  |
| 5042 | NCT00794222 | A Mobile Phone Self-Monitoring Tool to Increase Emotional Self-Awareness and Reduce Depression in Young People | Unclear |  |
| 5016 | NCT01958398 | Brief Alcohol Interventions With Mobile Phone Applications for University Students: a Randomized Controlled Trial | Published | <https://ascpjournal.biomedcentral.com/articles/10.1186/1940-0640-9-11> |
| 5034 | NCT02064998 | Two Consecutive Randomized Controlled Trials Using Mobile Phone Applications for Risky Alcohol Use | Unclear | Says completed but no links (links listed below but none that match study) |
| 4534 | NCT02072252 | Mobile Phone App for Depression and Anxiety in Young Men Who Are Attracted to Men | Withdrawn |  |
| 5857 | NCT02205177 | Youth Mayo Clinic Anxiety Coach Pilot Study | Unclear | [results only in NCT: https://clinicaltrials.gov/ct2/show/results/NCT02205177](https://clinicaltrials.gov/ct2/show/results/NCT02205177) |
| 4530 | NCT02265978 | CopeSmart: using Mobile Technology to Promote Positive Mental Health in Young People | Unclear | Says completed but no links (links listed below but none that match study) |
| 4527 | NCT02272179 | Brief Intervention for Suicide Risk Reduction in High Risk Adolescents | Published | [1. https://clinicaltrials.gov/ct2/bye/rQoPWwoRrXS9-i-wudNgpQDxudhWudNzlXNiZip9Ei7ym67VZR0BxgFwaRFwA6h9Ei4L3BUgWwNG0it. 2. https://clinicaltrials.gov/ct2/bye/rQoPWwoRrXS9-i-wudNgpQDxudhWudNzlXNiZip9Ei7ym67VZRFtER0jWgCwA6h9Ei4L3BUgWwNG0it.](https://clinicaltrials.gov/ct2/bye/rQoPWwoRrXS9-i-wudNgpQDxudhWudNzlXNiZip9Ei7ym67VZR0BxgFwaRFwA6h9Ei4L3BUgWwNG0it.) |
| 4526 | NCT02286466 | Mobile App of CBT for Anxiety and Cancer | Unclear | [results only in NCT: https://clinicaltrials.gov/ct2/show/results/NCT02286466?term=NCT02286466&draw=2&rank=1](https://clinicaltrials.gov/ct2/show/results/NCT02286466?term=NCT02286466&draw=2&rank=1) |
| 4580 | NCT02530645 | Development and Testing of a Smartphone Application to Reduce Substance Use and Sexual Risk Among Homeless Young Adults | Unclear | Says completed but no links |
| 4579 | NCT02567890 | Swedish Body Project for Prevention of Eating Disorders | Published | <https://content.apa.org/fulltext/2020-43103-003.html> |
| 5887 | NCT03032952 | The Efficacy of a Mobile Application for Treating Depression and Anxiety Symptoms | Published | <https://www.ncbi.nlm.nih.gov/pmc/articles/PMC7428915/> |
| 5880 | NCT03096171 | Flourishing App: an Evaluation With High School Students | Unclear | Says completed but no links |
| 5877 | NCT03274934 | The Effectiveness of the Mobile-based YouthCOMPASS Program to Promote Adolescent Well-being and Life-control | Unclear | Says completed but no links |
| 5874 | NCT03301012 | Smartphone Addiction Recovery Coach for Adolescents (SARC-A) Experiment | Ongoing |  |
| 5868 | NCT03361475 | SME(Sharing, Mind & Enjoyment) App for Adolescents | Unclear | Says completed but no links |
| 5866 | NCT03371810 | Prevention of Comorbid Depression and Obesity in Attention-deficit/ Hyperactivity Disorder | Ongoing |  |
| 5865 | NCT03378505 | Impact of Mobile App on Purpose and Well-Being Among College Students | Unclear | Says completed but no links |
| 4556 | NCT03406052 | Smartphone-enabled Health Coaching Intervention for Youth Diagnosed With Major Depressive Disorders | Suspended |  |
| 4555 | NCT03412461 | Bridging the Gap - Tools for Finding Health, Mental Health and Wellness Resources for University and College Students | Unclear | Says completed but no links (links listed below but none that match study) |
| 5859 | NCT03514004 | Using Information and Communication Technologies to Prevent Suicide in Chile | Published | <https://www.ncbi.nlm.nih.gov/pmc/articles/PMC5996076/> |
| 5765 | NCT03552900 | Evaluating a Mobile App for Students Seeking Care for Depression and Anxiety at Harvard University Health Services | Unclear | Says completed but no links (links listed below but none that match study) |
| 5757 | NCT03610672 | Mobile Intervention for Young Opioid Users | Unclear |  |
| 5768 | NCT03712319 | Efficacy of a Mobile Application vs MBSR in Healthcare Students | Unclear | Says completed but no links (links listed below but none that match study) |
| 4673 | NCT03783793 | Effects of a Mindfulness Meditation App on Subjective Well-Being in Undergraduate University Students | Published | <https://www.ncbi.nlm.nih.gov/pmc/articles/PMC6329416/> |
| 5743 | NCT03825588 | Establishing Efficacy of an Inpatient Intervention and Phone App to Reduce Suicidal Risk | Ongoing |  |
| 5018 | NCT03829527 | Evaluation of the Treatment Approach ROBIN | Ongoing |  |
| 4667 | NCT03900416 | Adolescent Mindfulness Mobile App Study (RCT) | Ongoing |  |
| 4665 | NCT03909685 | Examining the Feasibility of the Ask RoSE Mobile Mental Health Application | Ongoing |  |
| 4661 | NCT03913013 | Technology Enhanced Family Treatment | Ongoing |  |
| 4657 | NCT03940508 | Engaging Black Youth in Depression and Suicide Prevention Treatment Within Urban Schools | Ongoing |  |
| 4643 | NCT03990870 | Wiring Adolescents With Social Anxiety Via Behavioral Interventions | Ongoing |  |
| 4642 | NCT04000399 | BRITEPath, Component 3 of iCHART (Integrated Care to Help At-Risk Teens) | Ongoing |  |
| 4630 | NCT04052529 | Tracking Our Lives Study | Unclear | Says completed but no links |
| 4605 | NCT04081662 | Examining the Efficacy of Acceptance and Commitment Therapy (ACT) Microinterventions for Distressed First-Generation College Students | Ongoing |  |
| 4628 | NCT04089007 | Feasibility, Acceptability and Effectiveness of the SOmNI Mobile Phone App for Sleep Promotion in Adolescents | Unclear | Says completed but no links |
| 4602 | NCT04094792 | Depression, Trauma, and Health: efficacy of an mHealth App for Symptom Self-Management in College Students | suspended |  |
| 4601 | NCT04103606 | A Mobile-App Training to Reduce Body Image Disorder Symptoms and Associated Features in Female University Students | Ongoing |  |
| 4594 | NCT04148508 | App-based Mental Health Promotion in Young European Adults | Ongoing |  |
| 4589 | NCT04162847 | Harnessing Mobile Technology to Reduce Mental Health Disorders in College Populations | Ongoing |  |
| 4593 | NCT04164654 | A Mobile App to Address Student Loneliness | Ongoing |  |
| 5081 | NCT04169724 | Meditation for PA Students | Unclear | Says completed but no links |
| 4720 | NCT04213846 | Alcohol & Mobile Phone Study to Reduce High-risk Alcohol Use and Consequences | Ongoing |  |
| 4719 | NCT04218682 | Shadows Edge Mobile Developing Resilience in Adolescent and Young Adult Cancer Survivors | Ongoing |  |
| 5133 | NCT04463654 | Zero Self-Harm - a Mobile Phone Application to Reduce Non-suicidal Self-injury: a RandomizedClinical Superiority Trial | Ongoing |  |
| 5158 | NCT04519008 | Effectiveness and Security Testing of a Mobile App | Ongoing |  |
| 5168 | NCT04524598 | A CBT-based Mobile Intervention as First Line Treatment for Adolescent Depression During COVID-19 | Ongoing |  |
| 5153 | NCT04550286 | Study Smart! Effectiveness of a Smartphone Use Intervention on Students' Performance and Well-being | Ongoing |  |
| 5167 | NCT04558411 | Pilot Study of a Brief, Scaleable Intervention for Coronavirus (COVID-19) Mental Health Sequelae in College Students | Ongoing |  |
| 4760 | NCT04603053 | Determining Effectiveness of an mHealth Intervention to Provide Adolescent CBT | Ongoing |  |
| 4769 | NCT04636840 | Leveraging Social Media to Identify and Connect Teens With Eating Disorders to a Mobile Guided Self-Help Mobile Intervention | Ongoing |  |
| 4819 | NCT04672798 | BRITEPath- Phase 2 | Ongoing |  |
| 5252 | NCT04697966 | Mechanisms and Predictors of Change in App-Based Mindfulness Training for Adolescents | Ongoing |  |
| 5260 | NCT04719858 | Effect of #LIFEGOALS on Adolescents' Mental Health | Ongoing |  |
| 5247 | NCT04741529 | Massed and Spaced HMP App Dosage Study | Unclear | Says completed but no links |
| 5365 | NCT04861311 | Testing the Efficacy of a Mindfulness- and Acceptance-Based Smartphone App for PTSD: a Randomized Controlled Trial | Ongoing |  |
